# In Silico Characterization of ADAR1: Structure, Dynamics, and Functional Implications

**DOI:** 10.3390/cimb47110958

**Published:** 2025-11-18

**Authors:** Carolyn N. Ashley, Emmanuel Broni, ChaNyah M. Wood, Whelton A. Miller

**Affiliations:** 1 Department of Medicine, Loyola University Medical Center, Loyola University Chicago, Maywood, IL 60153, USA; cashley1@luc.edu (C.N.A.);; 2 Department of Molecular Pharmacology & Neuroscience, Loyola University Medical Center, Loyola University Chicago, Maywood, IL 60153, USA

**Keywords:** molecular dynamics simulations, principal component analysis, RNA editing, ADAR1 enzyme

## Abstract

Adenosine deaminase acting on RNA 1 (ADAR1) is an essential RNA-editing enzyme responsible for the hydrolytic deamination of adenosine to inosine (A-to-I) in double-stranded RNA. This editing mechanism plays a critical role in gene regulation, particularly in neural and immune contexts. Dysregulation of ADAR1 activity has been implicated in neurological disorders, cancer progression, and immune dysfunction, making ADAR1 an emerging therapeutic target. However, progress in therapeutic development has been hindered by the lack of structural insight into the full-length protein and how its dynamic behavior influences RNA-editing specificity and protein–protein interactions. In this study, we present computational models of the full-length ADAR1p150 isoform generated by homology modeling and further analyzed using molecular dynamics (MD) simulations and principal component analysis (PCA). Our analyses reveal that the dsRBD3 and CDD remain structurally stable, crucial for protein binding and catalytic function, whereas ZBDs and dsRBD1/2 exhibit extensive flexibility, particularly in inter-domain loops, facilitating RNA recognition indicative of conformational selection and fly-casting mechanisms. Free-energy landscape mapping identifies multiple low-energy conformations, highlighting conserved domain cores and flexible loop arrangements. Together, these findings underscore the importance of ADAR1’s dynamic architecture in regulating its function. By linking static structural information with dynamic behavior, the full-length models and dynamic insights presented here provide a valuable framework for future studies of ADAR1 complex formation, editing specificity, and therapeutic targeting.

## 1. Introduction

Adenosine deaminase acting on RNA 1 (ADAR1) catalyzes the conversion of adenosine to inosine (A-to-I) in RNA. This RNA editing occurs commonly in non-coding sequences such as untranslated regions (UTRs), introns, and repetitive elements, but can also occur in coding regions, modulating protein function and gene expression [1]. Millions of editing sites have been identified across the human transcriptome, particularly in the brain and immune-related tissues, where under normal physiological conditions, ADAR1 editing is necessary for proper neural development, immune homeostasis, and stress response [2,3,4,5]. Dysregulated RNA editing has been linked to various pathological states. Aberrant editing patterns can influence molecular pathways, altering receptor signaling in the brain, regulation of immune signaling, oncogenic pathways, or disturbing host–pathogen interactions [6,7,8]. Overactivity of ADAR1 can suppress type I interferon responses, contributing to resistance to immune checkpoint blockade therapies in cancer [9], while loss of ADAR1 activity can result in rampant interferon signaling and chronic inflammation in autoimmune contexts [10,11,12,13]. ADAR1 has two isoforms, ADAR1p150 and ADAR1p110. The ADAR1p150 isoform is primarily cytoplasmic and results from an interferon-inducible promotor, whereas ADAR1p110 is a dominantly nuclear isoform with constitutive expression. The ADAR1p150 isoform contains a Zα binding domain (ZαBD) in its N terminus, which is crucial for ADAR1′s role as an RNA surveillance protein, acting to distinguish self from non-self RNA and maintaining tight regulation of innate immune signaling [14].

ADAR1 is a therapeutic target; however, current therapeutics are limited by off-target RNA editing caused either by a lack of substrate specificity or by consequential unaccounted mechanisms [15,16]. ADAR1p150 has multiple functional domains, including two Z-DNA binding domains (ZBDs), Zα and Zβ, three double-stranded RNA-binding domains (dsRBDs), and an active catalytic domain (CDD). Experimental structures of ADAR1 domains have been captured by nuclear magnetic resonance (NMR) spectrometry, X-ray crystallography, and cryogenic electron microscopy (cryo-EM), revealing mechanisms influencing substrate recognition and dimerization. However, the full-length structure remains unresolved, limiting understanding of inter-domain arrangements and dynamic behaviors that impact RNA-binding and catalytic activity. Computational modeling and molecular dynamics (MD) simulations offer another variety of tools to construct and analyze the full-length conformations and domain motions, complementing static experimental data.

This study integrates homology modeling, MD simulations, and principal component analysis (PCA) to generate and refine full-length structural models of ADAR1p150. Homology modeling leverages available ADAR1 and ADAR2 structural templates to build models of the multidomain architecture. MD simulations enable the exploration of conformational flexibility, domain motions, and functional transitions, while PCA identifies the most significant motions. These computational approaches bridge static structural data with dynamic and functional insights, providing a foundation for rational structure-based drug design, selective inhibitor development, and interpretation of RNA-editing mechanisms.

## 2. Methods

In the absence of experimentally determined full-length models of the ADAR1 protein, computational modeling techniques were employed to generate reasonably accurate structural models. The models underwent MD refinement and PCA to evaluate stability and dynamics.

### 2.1. Sequence Retrieval

The protein sequence for ADAR1 in fasta format was obtained from UniProtKB with UniProtKB ID: P5526, title DSRAD_HUMAN [17]. The full ADAR1p150 sequence of 1226 amino acids was used, and for analysis of individual ADAR1 domain structures, the reported domain sequence data from P5526 was utilized.

### 2.2. Template Search and Obtaining Template Structures

Templates for ADAR1 were identified by searching with SWISS-MODEL (accessible via https://swissmodel.expasy.org/ accessed on 19 September 2023) and the National Center for Biotechnology Information (NCBI) Basic Local Alignment Search Tool (BLASTp) for protein data bank proteins [18]. Identified protein templates with structural data were retrieved from the Research Collaboratory for Structural Bioinformatics Protein Data Bank (RCSB PDB) [19]. A summary of templates is provided in Table S1 ([20,21,22,23,24,25,26,27,28,29]). Potential templates were accessed for the highest percent sequence identity, best residue coverage, and lowest resolution.

### 2.3. Homology Modeling

To generate full-length ADAR1 models, a combined strategy was employed to leverage the strengths of different modeling platforms. Rosetta contributed de novo fragments to improve predictions of flexible and poorly conserved looping regions, while inclusion of the AlphaFoldDB model (AF-P55265-F1) provided highly accurate well well-aligned templates for the ordered domains. Modeller10.5 was then used to integrate these inputs along with cryo-EM structures released after initial modeling, generating 5 models dubbed AlphaFold-included models.

#### 2.3.1. RosettaCM and Modeller

To initially predict the ADAR1 structure, a combination of comparative modeling approaches was utilized, including RosettaCM [30] and Modeller9.20 [31,32]. The ADAR1 sequence (P5526) was uploaded to the Robetta server (robetta.bakerlab.org), which has a maximum chain length of 1000 residues. Sequence chunks 1–250 amino acids (aa), 150–350 aa, 240–410 aa, 360–550 aa, 500–750 aa, 700–900 aa, and 1–886 aa were uploaded, and the comparative modeling option was selected. The sequence chunks spanned each of the domains with overlap on either side. For portions of the sequence that align with known PDB structures, these structures are built into the 3-D model [30]. Regions of the sequence without good template structural data are built through sampling torsion angles to define the protein backbone and side chain conformations [30]. The best selected model for each residue range was used as a template for model building. Five predicted models for each residue range of ADAR1 were generated and selected by confidence score, angstrom error estimate, and lowest RMSD values to solved structures. Sequences of the ADAR1 chunks and the sequences from the published ADAR1 structures were used to generate a multiple sequence alignment. The selected protein templates, along with the multiple sequence alignment, were then used via Modeller9.20 to generate five models. Other PDB templates included for comparative modeling with Modeller were 1XMK [23], 2GXB [22], 2MDR [27], and the homology model of the ADAR1 CDD [29]. At the time of initial model generation, PDBs: 7ZJ1 and 7ZLQ of the third dsRBD, 9B83, 9B84 of the CDD, and 9B89 with the CDD and 3dsRBD, had not been released [26,28].

#### 2.3.2. Improving Homology Modeling with AlphaFold

The initial homology modeling method utilized RosettaCM and Modeller9.20. These methods are useful for the resolution of looping regions and for modeling sequences with no homologous templates [30,32,33]. An AlphaFold Protein Structure Database (AlphaFoldDB, https://alphafold.ebi.ac.uk, last accessed on 31 July 2025) [34] model was predicted on UniProt as AF-P55265-F1-v6. Limitations of AlphaFold can include domain-swapping or errors in linking regions [34]. AlphaFold2 has been assessed for accuracy within looping regions and can do well with short loops, but accuracy decreases with larger loops [35]. Also, AlphaFold 2 can overpredict secondary structures, particularly helices [35]. To overcome the limitations of relying on a single modeling strategy, Modeller10.5 was utilized to incorporate the Robetta fragments that have strong predictive power for the looping regions and the AF-P55265-F1 model that had strong predictive power for the ordered domains, in addition, newly published cryo-EM structures were also included in the second round of modeling to generate five new models referred to as AlphaFold-included models.

### 2.4. Molecular Dynamics Simulations-Based Model Refinement

An initial 100 ns force field evaluation was performed using ADAR1p150 model 1 with AMBER99SB-ILDN [36], CHARMM36 [37], and OPLS-AA/L force fields [38,39]. In total, 1.1 µs MD simulations were performed on the predicted models with GROMACS version 2023.3 [40,41]. Protein MD simulations used pdb2gmx for modeling the hydrogen atoms onto the predicted models. Models were solvated, where AMBER and CHARMM36 forcefields used the tip3p water model, and OPLS used the tip4p water model in a cubic box with 2.0 nm of solvent padding distance between the protein and the edge of the box in all directions. Solvent molecules were replaced with Na and Cl ions to neutralize the system, and a salt concentration of 12 mM was set within the range of physiological intracellular sodium concentration [42]. For the parameters of the MD simulations, LINCS was used for h-bond constraints, and a Verlet cutoff scheme with a 1 nm cutoff was used for the short-range interactions, specifically van der Waals interactions and electrostatic interactions. Electrostatic interactions were computed in the long-range electrostatics using Particle Mesh Ewald (PME). Each system underwent energy minimization using steepest descent, where nsteps were 50,000, followed by equilibration first by NVT for 50,000 steps and then NPT for 50,000 steps. After equilibration, systems were submitted to production runs with a dt of 0.002 fs, and coordinates were saved every 10.0 ps. Each of the five models was run from the 100 ns initial run for an additional 800 ns with the CHARMM36 force field. This method was repeated using the second round of models that included the AlphaFoldDB and cryo-EM templates.

### 2.5. Evaluation of Structural Changes and Dynamics

Trajectory analyses using root mean square deviation (RMSD), radius of gyration (Rg), and root mean square fluctuation (RMSF) for the 5 protein models were evaluated after the first 100 ns and following 800 ns. RMSD of the backbone during simulation relative to the initial backbone at the start of each time point was evaluated. Local similarity to known PDB templates was also evaluated in the models by RMSD via the align function by TMalign (https://zhanggroup.org/TM-align/, accessed in 5 March 2025) [43]. Other simulation parameters, including Rg and RMSF, were used to evaluate the compactness and local flexibility within the models, respectively. For each of the models generated, the protein structure in PDB format is provided post-MD simulation in the supplementary materials. Principal component analysis (PCA) was performed to assess the dynamic behavior of the protein and to identify the dominant motions of the ADAR1 structural models. PCA can reduce high-dimensional data into principal components that characterize the dominant motions within an MD simulation [44]. These motions can contribute to understanding protein folding and can identify transitions into stable or metastable states of the protein [45]. For PCA, a workflow from GROMACS was followed [46].

### 2.6. Model Quality Assessment

The structural quality and reliability of the predicted models were evaluated using a combination of computational validation tools. Initial model assessment used the molecular pdf (molpdf) score, a summary of total constraints, from Modeller. Protein Structure Analysis, ProSA-web [47], and the Structure Analysis and Verification Server v6.1 (SAVEs, https://saves.mbi.ucla.edu/, assessed in 7 March and 16 June 2025) were employed to assess overall model quality. ProSA-web was used for comparison of computational models to experimental high-resolution crystal or NMR structures, and for evaluating energy profiles to identify problematic local errors or potentially misfolded segments. SAVEs has several tools for protein quality assessment, including VERIFY3D [48], ERRAT [49], and PROCHECK [50], that were used to identify structural steric clashes, improper bond geometry, and conformational outliers. ERRAT relies on statistics of non-bonded atom–atom interactions in the model compared to high-resolution experimental structures to determine confidence levels of the models [49]. VERIFY3D tests the accuracy of a 3D model by comparing it to its own 1D amino acid sequence to assess whether each residue matches the environment expected from other experimental structures [48]. A high-quality model is expected to have a VERIFY3D score of ≥80%. PROCHECK was used to look at the stereochemical quality of the predicted models and generate Ramachandran plots. For PROCHECK, models with stable and common conformations are expected to have more than 90% of residues within the favorable regions [51].

## 3. Results and Discussion

### 3.1. ADAR1 Structures

Homology modeling can yield accurate protein models when suitable templates with high sequence similarity are available [52]. Confidence of predicted models improves above 30% similarity in template structure, and model accuracy has better performance with templates above 50% [52,53]. There is a repertoire of isolated domain structures for ADAR1 published in the Research Collaboratory for Structural Bioinformatics Protein Data Bank (RCSB PDB, RCSB.org) [54,55] (Table S1). The publications of these individual structures have been possible through diligent work by numerous lab groups using a toolbox of experimental structural methods, including NMR spectroscopy, X-ray crystallography, and, most recently, cryo-EM. Cryo-EM studies resolved the catalytic domain of ADAR1 but failed to capture highly flexible regions such as the ZBDs and dsRBDs, which contribute to RNA recognition but remain challenging to visualize [28,56,57]. Previously, homology modeling in combination with experimental constraints was used to generate an ADAR1 CDD protein structure, and this structure aligns well with the captured cryo-EM structure [28,29]. Due to the limitations of experimental approaches, initial full-length ADAR1 models were first generated with Modeller using available templates and de novo segment predictions from Robetta. Following the assessment of these models, cryo-EM structures (PDBs: 9B89, 9B83, and 9B84) became available [28]. Also, AlphaFold DB provided an improved model for structured domains. Incorporating the cryo-EM structures and the AlphaFold model as additional templates enabled the construction of a second generation of refined ADAR1 models, referred to as AlphaFold-included models.

### 3.2. Template Search and De Novo Fragment Generation

A protein template search for homologues of ADAR1, UNIPROT ID: P55265, with PDB structures was initiated using the NCBI BLASTp and SWISS-MODEL BLAST (https://swissmodel.expasy.org/, accessed in 5 March 2025). At the time of this search, cryo-EM data for ADAR1 and more templates (PDBs: 7ZJ1 and 7ZLQ) of the dsRBD3 were not available. The SWISS-MODEL template search resulted in a total of 4901 templates with matches to the ADAR1 sequence, but the SWISS-MODEL heuristic filtered down to 41 templates after removal of low-quality and irrelevant hits. All ADAR1 structures were selected as templates. ADAR1 and ADAR2 share a sequence similarity of 55% and a sequence identity of 39% [58]. Other top templates included RNA-binding proteins and proteins containing dsRBDs or ZBDs, which was expected due to the structural topology and conservation of contact residues within these binding domains [23,59,60,61]. The NCBI BLAST reported 26 sequences, where 11 hits had percentage identities of 60% or greater.

### 3.3. Modeling with Robetta and Modeller

The availability of multiple high-quality structural templates with significant sequence similarity to the target protein provided a strong rationale for employing homology modeling to generate 3D structural predictions. While there were many templates for the ADAR1 structure, there were also large gaps in the template structures spanning residue regions 1–124, 367–502, 683–707, and 802–832. Additionally, regions 210–292 and 572–613 were reported to be intrinsically disordered on UniProt. These regions were submitted to the Robetta server, which uses a combination of a comparative model approach utilizing the known structural templates and de novo fragment generation for regions with no template or poor homology [30,62]. These protein regions were then assessed by confidence score and the Angstrom error estimate (Table S2 and Figure S1). The confidence scores provided by Rosetta predictions look at the convergence of the best clusters [63]. Prior study supports that when the top models generated from multiple clusters predict similar structures, the confidence of the model accuracy is greater [63]. Rosetta also gives an estimate of the per-residue precision, which was evaluated for areas with high and low error estimates. In evaluating the individual residue regions, areas with very low estimated Cα RMS error included regions: 145–200, 300–345, 503–575, 614–680, and 740–795, that directly correspond to the known ADAR1 structure domains (Figure S1). Although there is no structure of the first or second dsRBD of ADAR1, there are structures of the ADAR2 dsRBDs and the third dsRBD of ADAR1. The dsRBDs of ADAR1 and ADAR2 have a high percentage similarity, with the dsRBDs of ADAR2 matching best with the first and third dsRBDs of ADAR1 [58]. Modeller9.20 was then employed to generate five full-length structural models using the first model for each of the residue ranges and the identified ADAR1 structures as templates for the multisequence alignment. The five models were ranked by molfpdf, which scores based on spatial constraints, where lower scores are better. The models ranked model 1, model 4, model 2, model 5, and model 3 with molpdf scores of 145,680.04688, 147,192.57812, 147,838.70312, 149,266.92188, and 153,572.29688, respectively.

### 3.4. Modeling Improvement with AlphaFold DB

**Figure 1:**
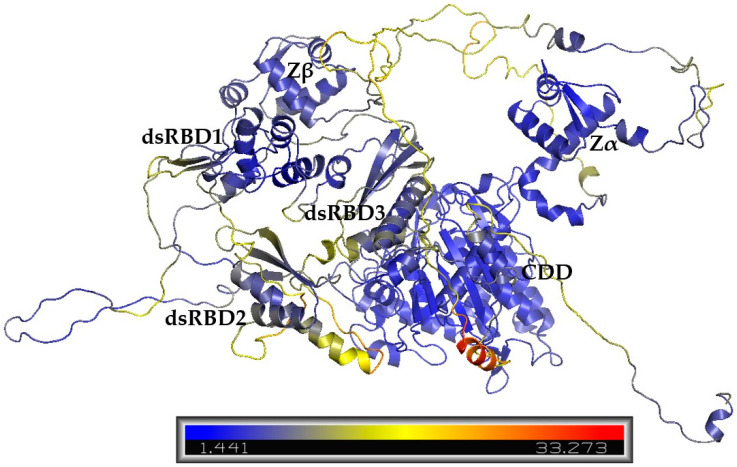
RMSD comparison of AlphaFold-included models. The color spectrum from blue to yellow to red indicates structural variability, with blue highlighting the conserved regions and red marking areas of highest deviation across the models.

Experimental structures of ADAR1 domains Zα (PDB: 1QBJ), Zβ (PDB: 1XMK), dsRBD3 (PDB: 2MDR), CDD (PDB: 9B83), and dsRBD3–CDD (PDB:9B89) had the lowest resolution and largest coverage length of the published structures for ADAR1 domains. The initial models aligned well with the structures for 1QBJ and 1XMK, with Cα-RMSD values less than 2 Å for PDBs, and PDB 2MDR with Cα-RMSD values ranging from 1.98 to 2.27 Å (Table S3). However, alignment with PDBs 9B83 and 9B89 that covered the CDD was poor, with Cα-RMSDs ranging from 5.54 to 6.87, indicating that the CDD was not well captured. Model quality assessment by SAVEs also reflected room for improvement of the initial models, where the ERRAT scores were less than 29.04, VERIFY 3D scores were below 53.08%, and PROCHECK reported less than 75% of residues in the favored regions (Table S4 and Figure S2). The release of the AlphaFold DB structure for ADAR1 (AF-P55265-F1) provided a substantially improved template. Model AF-P55265-F1 showed drastic improvements in the alignments to the structural templates with Cα-RMSD values of 0.77, 1.84, 1.55, 1.08, and 3.07 to PDBs 1QBJ, 1XMK, 2MDR, 9B83, and 9B89, respectively (Table 1). In comparison to the initial models, model AF-P55265-F1 also showed vast improvement in SAVEs with an ERRAT score of 95.3737, and a higher VERIFY3D score of 61.42% (Table 1 and Table S4). These results demonstrate that AlphaFold’s model more accurately captured the structured domains of ADAR1 compared to initial homology modeling. To improve model quality, a second round of homology modeling was performed using Modeller10.5, incorporating the AlphaFold model, AF-P55265-F1, and newly published cryo-EM structures. While AlphaFold improved the model quality results for ordered domains, concerns remained regarding linking regions and loops. They ranked model 4, model 3, model 5, model 1, and model 2 with molpdf scores of 85,828.26562, 89,784.28125, 92,985.09375, 95,629.57031, and 96,981.09375, respectively. Comparison by root mean squared deviation (RMSD) showed that the largest differences between models were in long looping regions, and slight variability between ZBDs and dsRBDs 1 and 2 (Figure 1). Model 2 was the most different from the other four models, with the largest RMSD values in dsRBD2 (Figure S3).

### 3.5. Evaluation of Model Quality

Structural alignment, ProSA, and SAVEs v6.1 tools ERRAT, VERIFY3D, and PROCHECK were used to evaluate the model quality of the AlphaFold-included models.

#### 3.5.1. Structural Alignment Assessment

**Figure 2:**
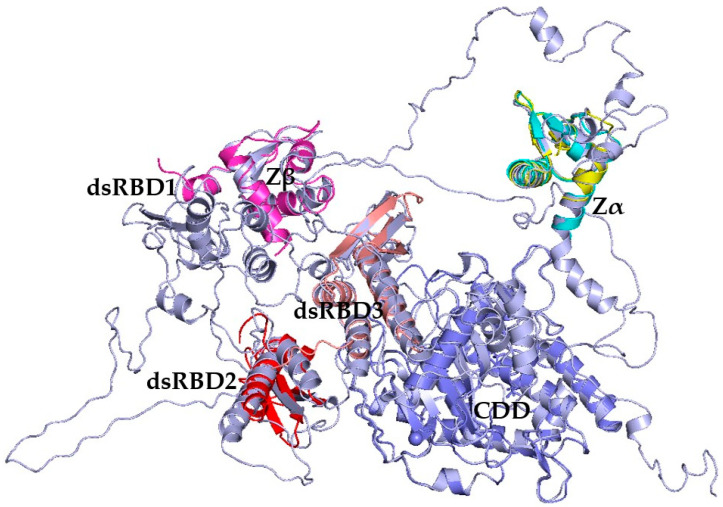
Overlay of PDB templates: 1QBJ (cyan), 2GXB (yellow), 1XMK (pink), 2MDR (salmon), 9B83 (slate), 2B7T (red) onto model 4.

Comparison of the AlphaFold-included models to AF-P55265-F1, showed improved structural alignments to the ADAR1 structures, 1QBJ, 1XMK, 2MDR, 9B83, and 9B89 (Table 1). For each model, the PDB structures appeared with less than 3.06 Å Cα-RMSD values (Table 1). Model 4 best represented the experimental structures with low Cα-RMSD values, all less than 2.12 Ås (Table 1 and Figure 2). These five models were comparable or better aligned to the ADAR1 structures than AF-P55265-F1 (Table 1).

#### 3.5.2. Protein Structure Analysis

ProSA of the AlphaFold-included models indicated good overall structural quality (Figure S4). These models achieved z-scores of −10.58, −10.90, −11.21, −10.72, and −11.63 for models 1 through 5, respectively. These Z-scores fall within the expected range of Z-scores for structures of the same size captured by X-ray crystallography. Also, from the local quality plots for each of these models, most of the positions stayed in the negative range, and the portions in the positive range corresponded to intrinsically disordered or long looping regions (Figure S4). Notably, Z-scores of the initial models fell outside the expected range and showed local model quality plots with mostly positive energies (Figure S5). These findings support that the incorporation of the AlphaFold model and cryo-EM templates did significantly affect the predicted model outcome.

#### 3.5.3. Model Quality with SAVEs

Structural quality of the AlphaFold-included models was assessed by SAVESv6.1 server. PROCHECK showed that in each of the five models, more than 80% of the residues were in favored regions and less than 2% of residues were in disallowed regions (Table 2 and Figure S6). The VERIFY3D scores ranged from 67.58% to 71.23% and the ERRAT scores ranged from 52.9412 to 61.7058 (Table 2). Compared to AF-P55265-F1, PROCHECK scores and VERIFY3D scores improved across all five models; however, the ERRAT scores were much lower (Table 2). An ERRAT score of 95 is very high and indicates very few non-bonded atom interactions deviating from expected high-resolution structural patterns. Notably, flexible loops and intrinsically disordered regions have inherent flexibility and various conformations, so they often lack consistent non-bonded interaction patterns observed in secondary structures, which can affect ERRAT scores. These results, in addition to the structural alignments, support that the AlphaFold-included models accurately represent the ordered regions of the ADAR1 protein.

### 3.6. Model Refinement with MD Simulations

MD simulations have been shown to significantly improve model structures that deviate from experimentally determined structures, at least in small and medium-sized proteins [64]. In preparing for MD simulations, the models undergo energy minimization by steepest descent, which allows for relaxation of the protein and can resolve some of the atomic clashes, achieving a more native protein state. The AlphaFold-included models were run for a force field comparison for an initial 100 ns and then run for an additional 800 ns using the CHARMM36 force field.

#### 3.6.1. 100. ns MD Simulations

**Figure 3:**
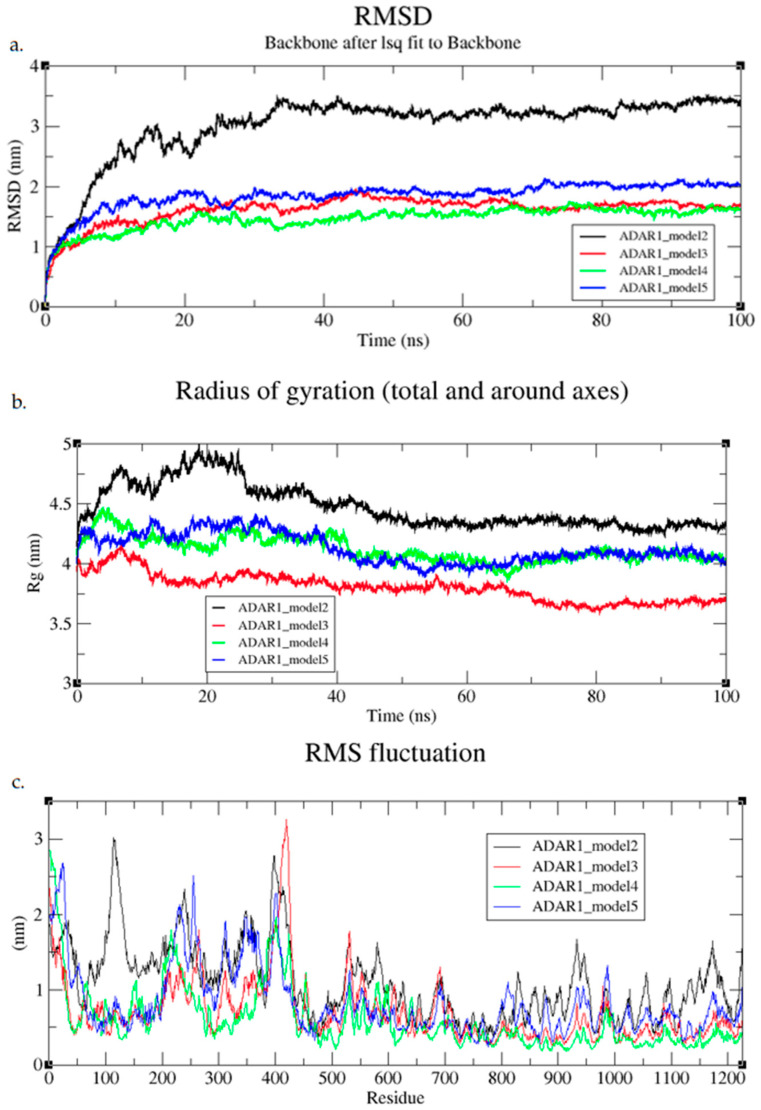
(**a**) RMSD, (**b**) Rg, and (**c**) RMSF plots of ADAR1 full-length AF included models during initial 100 ns MD simulations.

The AlphaFold-included models reached convergence during the first 100 ns. Issues within model 1 led to high strain and instability, and the model failed to complete energy minimization. This failure was due to a structural error caused by overlapping atoms and steric clashes. Models 3, 4, and 5 converged early on after around 10 ns (Figure 3). Model 4 remained the most stable over this time course, staying around 1.5 nm or less (Figure 3). The ability for the models to converge quite quickly supports that the initial conformations were reasonable (Figure 3). Model 2 had more instability initially and stabilized later in the MD run at around 40 ns and about 3.2 nm (Figure 3a). The Rg of models 3, 4, and 5 were relatively consistent, supporting that the protein was well folded (Figure 3b). Model 2 slightly unfolded during the first 25 ns before becoming more compact and stabilizing with Rg values around 4.5 nm (Figure 3b). This model may have become partially unfolded, or flexibility within the interdomain linkers may have sampled a more open protein state. The RMSFs of models 3, 4, and 5 differed from model 2 in one major region spanning from residues 50–200 (Figure 3c). This region overlaps with the intrinsically disordered region in the N-terminus and the location of the ZαBD. The higher RMSF values in model 2 in this region suggest some flexibility in the loop region, which could be biologically relevant dynamics of the domain moving or potential instability caused by missing stabilizing contacts.

#### 3.6.2. 800 ns MD Simulations

**Figure 4:**
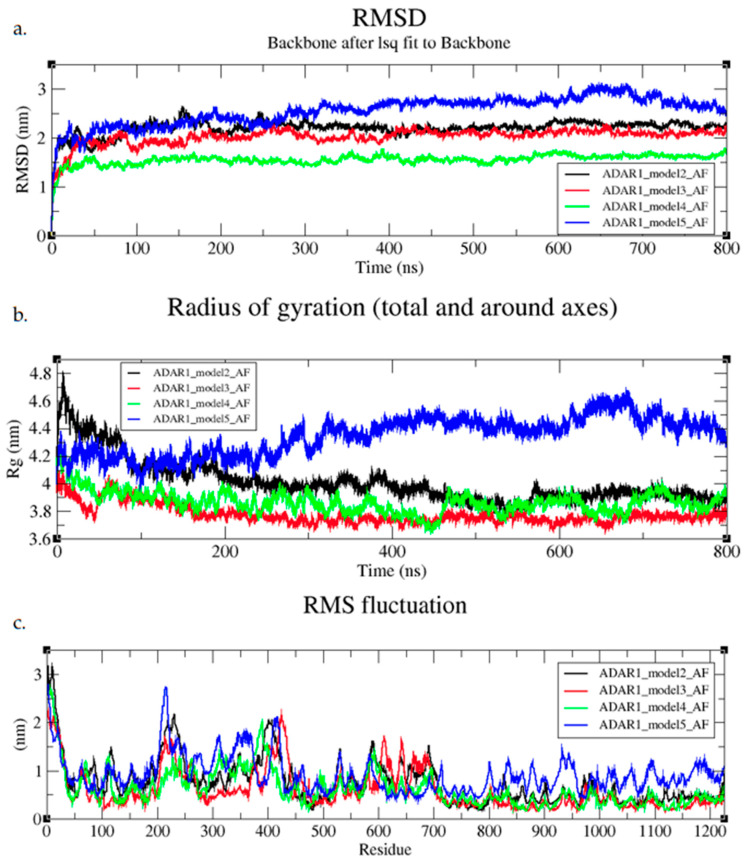
(**a**) RMSD, (**b**) Rg, and (**c**) RMSF plots of ADAR1 full-length AF included models during 800 ns MD simulations.

Following the 100 ns MD simulation, 800 ns MD simulations were run. By increasing the simulation time, we hoped to capture important motions and potential transitions between different metastable states of the protein models. The Alpha Fold models had mostly stable RMSD values throughout the 800 ns simulation (Figure 4a). Model 4 had the lowest RMSD values at around 1.5 nm (Figure 4a). Model 5 was stable up to around 300 ns and then began to gradually deviate from an RMSD of around 2 nm to around 3 nm (Figure 4a). The Rg at 300 ns also showed an increase in Rg values, shifting from 4.2 nm to 4.6 nm (Figure 4b). From the RMSF graph, residues 800–1226 corresponding to the CDD have higher fluctuations than the other 3 models (Figure 4c). This suggests that the CDD may have been reorienting and becoming more expanded or loosened in structure. The other three models maintained stable Rg and had low RMSF values in the CDD around 0.5 nm (Figure 4b,c). The RMSF graph did show flexibility from ranges 200–400 and 600–700 (Figure 4c). These areas cover linkers between domains, but also the Zβ and dsRBD2, where higher flexibility was expected.

#### 3.6.3. Post-MD Model Quality Analysis

Following MD simulation refinement, the quality of the output structures was evaluated. The AlphaFold-included models generally improved after the MD simulations. Post-simulation, the ERRAT scores ranged from 85.6275 to 88.9872 (Table 2). The PROCHECK statistics also improved with a range of 87.9% to 88.6% of residues in favorable regions (Table 2 and Figure S6). Having 90% of residues within the favorable region is expected for ideal or high-quality models. Also, PROCHECK showed a few residues in the disallowed region, spanning from 0.3% to 0.7% for the four models (Table 2 and Figure S6). The VERIFY3D scores of the models after MD simulation decreased compared to the initially generated models (Table 2). The VERIFY3D scores of the models, however, decreased compared to the initially generated structures (Table 2).

When assessing only the quality of the ordered domains, ERRAT scores improved after MD, with the highest being model 4, reaching an ERRAT score of 94.1964 (Table S5). This reflects a reduction in steric clashes and improved local packing following relaxation. In contrast, VERIFY3D scores decreased from greater than 80% to between 58.66 and 74.07%, likely due to movements during MD creating non-crystal-like local environments (Table S5). VERIFY3D therefore reported lower environmental compatibility despite improved local geometry in the ordered domains. PROCHECK statistics changed only marginally in the favorable region, with models 2 and 3 staying above 90% (Table S5). The ADAR1 structure is flexible, with looping regions and intrinsically disordered regions that can contribute to poor VERIFY3D scores. However, structural comparison to the known PDBs after MD simulation provided further evidence for the overall fold of the well-characterized domains (Table 3 and Figure S7). The AlphaFold models included had Cα-RMSD values between 1.69 and 3.3.22 Å for each of the PDB comparisons (Table 3). Model 3 was the best representation of the experimentally captured structures with Cα-RMSD values of 1.89, 1.82, 1.87, and 2.98 Å for the PDBs: 1QBJ, 1XMK, 2MDR, and 9B83, respectively (Table 3). Overall, this data supports that the models have good quality for the structured core but provide limited insight into the dynamic behavior of the domains, loops, and disordered regions. To capture these conformational fluctuations and explore the dominant motions sampled during the MD simulations, principal component analysis (PCA) was performed on the trajectories.

#### 3.6.4. MD Refinement of Initial Models

The lower-quality initial models were also refined by MD simulation to assess whether strong-quality models could still be generated after MD simulation. During the initial 100 ns simulations, most models converged by 50 ns, with the exception of model 3, which retained minor fluctuations (Figure S8a). The Rg indicated compaction of the structures by 20 ns except for model 1, which stabilized later around 50 ns and reached its most compact state at 4 nm (Figure S8b). From these two graphs, the RMSD was increasing, showing a structural drift from the initial structures, but the Rg mainly decreases, showing that the structures are becoming more compact. The RMSF for these initial structures during the first 100 ns stays low across the protein models, with most portions ranging between 0.3 and 1.5 nm (Figure S8c). One area of concern was the 550–700 area that covers the dsRBD1, dsRBD2, and linker regions between these domains (Figure S8c). While these could be highly flexible, it is also possible that these domains were poorly modeled, and MD let them move more freely. These are RNA-binding domains, so it is also possible that the domains remain highly flexible until the binding of RNA substrate.

In the extended 800 ns MD simulations, convergence occurred within the first 200 ns (Figure S9a). Rg values remained stable for models 1 and 5, and semi-stable Rg values for the other models (Figure S9b). There was a downward trend in Rg for models 3 and 4, finishing the 800 ns (Figure S9b). RMSF values showed minor fluctuations across the protein, mostly between 0.3 and 1 nm across residues (Figure S9c). There were some peaks in RMSF at regions 450–500 and 600–700 corresponding to the linker region between the Zβ domain and dsRBD1 and the regions spanning dsRBD2 and the linkers between dsRBD1-dsRBD2 and dsRBD2-dsRBD3 (Figure S9c).

Model quality improved notably after MD refinement. Prior to MD simulation, model 2 from the initial models had the highest ERRAT score of 29.0429, indicating poor overall quality (Table S4). Following the MD simulation, model 2 had an ERRAT score of 80.4752, a notable improvement in the reliability of the model (Table S6). The PROCHECK plot statistics of the initial models increased, having greater than 80% of residues in the favored region (Table S6 and Figure S2). The VERIFY3D scores showed minimal changes, suggesting that the local environment compatibility of individual residues was not significantly improved during the simulations (Table S6). Despite the ERRAT and PROCHECK improvements, even the refined models still deviated more from the experimental PDBs than the AlphaFold-included models (Table S7). In particular, the CDD region maintained high Cα-RMSD values (greater than 6 Å), and the TM-score did not support a similar folding, indicating that the CDD was not well represented (Table S7).

### 3.7. Principal Component Analysis

For large, highly dynamic proteins like ADAR1 with long flexible loops and intrinsically ordered regions, PCA enables the identification of the major conformational changes by evaluating the major variance of atomic displacements, i.e., PCA provides insights into the flexibility and functional motions of the protein. Using PCA, the critical motions of the protein can be revealed, which would otherwise be ignored by static structures or hidden by local fluctuations in a flexible protein.

#### Principal Component Analysis

**Figure 5:**
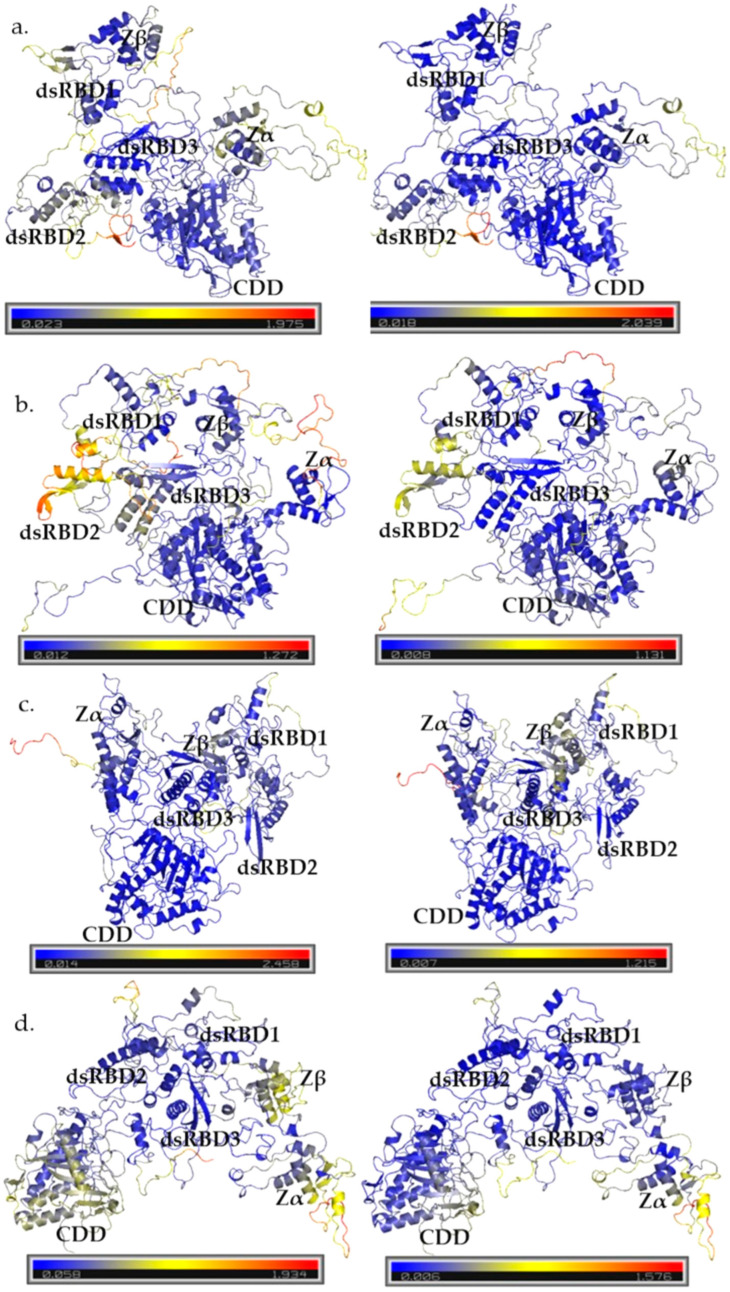
RMSF of PC1 (**left**) and PC2 (**right**) for AlphaFold-included models 2–5, (**a**–**d**). Color spectra span from blue to yellow to red, with blue representing areas that stay rigid and red representing areas with the highest flexibility.

Only the AlphaFold-included models, which best represented the experimental structures and showed higher overall quality, were selected for PCA. Scree plots showed that the first 2 principal components (PCs) captured the most significant motions, with additional PCs contributing subtle conformational changes (Figure S10). While cumulative variance analysis indicated that 12–22 PCs were required to explain 90% of total movement across models, the largest amplitude of motions was described by PC1 and PC2 (Figure S11). Analysis of PC1 and PC2 revealed consistent patterns: dsRBD3 and the CDD remained structurally stable, whereas Zα, Zβ, dsRBD1, and dsRBD2 displayed pronounced flexibility (Figure 5 and Figure S12 ). Within PC1 and PC2, the long interdomain loops often showed high variability, and these motions coincided with domain motions seen in collective movements in the projection plots (Figure S13). RMSF of PC3 further highlighted the movement of interdomain loops with other minor fluctuations in the ZβBD and dsRBD1 (Figure S14).

The dsRBD3 and CDD were the most stable domains with the lowest RMSF values across models (Figure S15). Crystallographic and biochemical data have demonstrated that the dsRBD3 can independently form stable dimers and is sufficient to mediate protein–protein interactions [26,27,57,65]. Our simulations reinforce this, as the dsRBD3 remained well packed in the models, with motions dominated by small breathing motions rather than large-scale domain motions (Figure 5 and Figure S13). This stability suggests that the dsRBD3 shape is critical for its multiple functions, not only as an RNA-binding protein but also as a scaffolding protein, dimer interface, and domain contacts with the CDD.

Interestingly, in model 5, the dsRBD3 shifted away from the CDD, resulting in increased RMSF within residues 826–864, 874–880, and 894–905 covering the loop between dsRBD3–CDD and the CDD (Figure 5d). Importantly, this range covers the active site and the highly conserved CHAE sequence, where mutations of H910 and E912 completely abolish RNA editing [66,67]. This increase in flexibility can explain why in experimental studies the independent CDD maintains editing activity, but at a lower efficiency than wild type [68]. A strict rigidity of residues in the early CDD supports that this may be a region contributing to RNA identification and interaction. Mutations within the early CDD: Ala870, Leu872, Arg892, Lys999, TyrY1112, and Asp1113, impair editing of short substrates but maintain editing of longer substrates [28]. The more rigid conformation along these residues is likely essential for the productive engagement of short substrates. Notably, ADAR1 can form dimers through its dsRBD3, but is capable of forming dimers in other modes. Co-immunoprecipitation experiments demonstrate that dsRBD3 provides a critical dimerization interface in the absence of the CDD, as mutations of the dsRBD3 abrogate ADAR1 dimer formation [26]. However, in the context of full-length ADAR1, dsRBD3 mutations alone are not sufficient to prevent dimerization, indicating that additional domains contribute to dimer formation [26]. ADAR2 forms an asymmetric dimer utilizing its CDD and dsRBD2 [69]. The dimerization helix identified in the ADAR2 CDD (PDB: 6VFF, residues 501–509; TWDG**VLQ**GE) is at least partially conserved in ADAR1 CDD, as seen in the cryo-EM structure (PDB: 9B89, residues 1021–1028; (TWDG**IRL**GE). This conservation suggests that, beyond its catalytic role, the ADAR1 CDD likely also contributes to dimer formation, similar to what has been observed for ADAR2. While the stability of the CDD and dsRBD3 contributes to their critical functions, other domains of ADAR1 exhibited markedly higher flexibility, with the largest motions concentrated in the interdomain loops and other RNA-binding domains.

Examination of PC1 and PC2 for each model highlighted flexibility in the ZαBD, ZβBD, dsRBD1, and dsRBD2, particularly in the loop regions and β-sheets that intermittently transitioned between ordered and disordered states (Figure 5 and Figure S12). The Zα and Zβ binding domains showed large domain movements; however, the core topology was mostly maintained with the exception of some disordering of the β1 sheet (Figures S16 and S19a,b). Previous structures of the apo and RNA/DNA duplex bound states of the ZαBD support that residues in helix α3: Lys169, Lys170, Asn173, and Tyr177, and in the β-hairpin: Lys187, Thr191, and Trp195, are critical for binding interactions (Table S8). Notably, residues Thr191, Pro192, Pro193, and Trp195 remain ordered in the apo- ZαBD state; the rigidness of these residues highlights an important recognition interface for Z-DNA [20,21,22]. Mutation of other more flexible residues, such as Gln50, can also reduce DNA binding affinity [21]. These results were mirrored by the flexibility of the ZαBD seen during MD simulation, but the rigidity of the primary backbone structure over time. In all, these data support that for the ZαBD, some interface contacts remain rigid for important contact interface residues, but other, more flexible residues can also contribute to binding affinity and likely also affect selectivity of some substrates. Interestingly, using constructs of the ZβBD alone and ZαBD and ZβBD together (Zαβ), the ZβBD alone showed stability, but the combined construct showed instability [70]. During simulations for models 2 and 5, the ZβBD showed higher flexibility between basins, and its movement was directly affected by the rearrangement of the ZαBD and high mobility of the Zα to Zβ linker (Figure S12).

Examinations of PC1 and PC2 for each model often showed large variation in the first and second dsRBDs (Figure 5). Observation of these regions across the different transition basins showed that the domains intermittently transitioned between ordered and disordered states (Figures S16–S19). RNA binding by the dsRBDs occurs through three conserved interfaces: helix α1, the β1-2 loop, and the KKxxK motif (Table S8) [26]. Deletion analyses revealed that removal of ZαBD, ZβBD, dsRBD1, or dsRBD2 has minimal impact on the editing of model substrates 5-HT2CR-71nt and Gli1-31nt [28]. In contrast, deletion or disruption of dsRBD3 almost completely abolishes editing activity of those substrates, highlighting its indispensable role in contributing to RNA recognition and potentially catalytic efficiency [28]. Together, these findings suggest a hierarchical contribution of the dsRBDs, where dsRBD3 provides a primary, high-affinity RNA-binding interface with strong structural constraints, while dsRBD1 and dsRBD2 contribute to more auxiliary interactions without the requirement for strict structural constraints. However, like ADAR2’s dsRBDs, the three interaction interfaces remain important for substrate recognition. The ordering and disordering motions of the ZBDs and dsRBD1 and dsRBD2 are indicative of conformational selection or fly-casting mechanisms that contribute to substrate recognition, consistent with their known roles in binding Z-DNA/RNA. These transient disordered states likely contribute to the difficulty of capturing these domains by standard structural methods. Such domains often exhibit dynamic motion, enabling mechanisms such as fly-casting and conformational selection during target engagement [71]. The intrinsic flexibility of RNA-binding loops allows them to dynamically search and adapt to diverse RNA surfaces efficiently [72]. In this context, conformational selection not only facilitates RNA-binding, but can also influence the formation of higher-order protein complexes [71].

Free-energy landscapes (FEL) allow us to identify what conformations were explored and the relative stability of each tested conformational state. The 2D FEL plots were colored by ΔG energy and by simulation time, so that the time at which the lowest-energy structures were sampled could be visualized (Figure S20). The 3D FEL was also graphed to better visualize the deepest basins, where the structures are colored the darkest purple and represent the lowest-energy structures (Figure S21). In FEL, distinct basins are generated, representing each conformational state. The deeper the basin, the more favorable the conformation thermodynamically. Shallow basins in between deep basins are indicative of transitional states. Representative structures were extracted from the minimum of each basin in the 2D FEL, defined by the frames closest to the centers of high-density regions in PC1–PC2 space. To visualize the transitional conformations and changes between dominant conformations, 10 structures from each basin were assessed by RMSD. To assess the most critical motions of the models, the 10 overall lowest-energy structures were extracted for each model.

**Figure 6:**
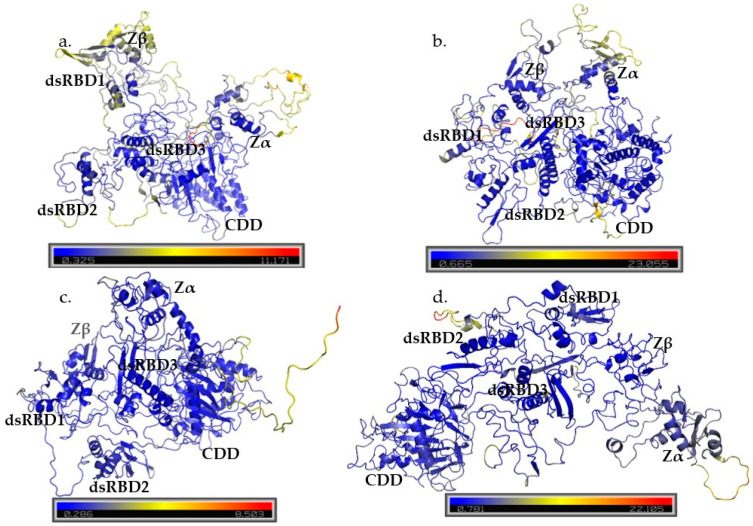
RMSD of the 10 lowest-energy conformations for each AlphaFold-included models 2–5, (**a**–**d**). Color spectra span from blue to yellow to red, with blue representing areas that stay conserved and red representing areas with the highest structural variation.

RMSD analysis of structures within individual basins highlights regions of structural variability among the dominant conformational states sampled during the MD simulations. Across all models, the largest RMSD fluctuations occurred in the inter-domain loop regions, particularly between the ZαBD-ZβBD and ZβBD-dsRBD1 linkers (Figures S22–S25). Model 2 displayed three primary basins, with the highest intradomain RMSD values in the dsRBD1 and ZβBD (Figure S22). The lowest-energy structures for model 2 were sampled between 500 ns and 600 ns and showed the greatest conformational changes in the β-sheets of the ZαBD (Table S9 and Figure 6). Model 3 exhibited four basins that captured extensive movement of the ZαBD (Figure S23). Its lowest-energy structures were distributed through the trajectory, suggesting that ZαBD mobility strongly influenced the overall energy landscape (Table S9 and Figure 6). Model 4 remained remarkably stable across basins (Figure S24), with its lowest-energy conformations concentrated between 700 and 800 ns (Table S9). Only the C-terminal intrinsically disordered region contributed to deviations in energy between the lowest-energy structures of model 4 (Figure 6). In contrast, model 5 showed the greatest variation between basins, reflecting progressive compaction and decompaction of the RNA-binding domains and CDD over time (Figure S25). The lowest-energy structures for model 5 occurred between 600 ns and 800 ns and showed minimal variance within the ordered domain cores (Table S9 and Figure 6).

**Figure 7:**
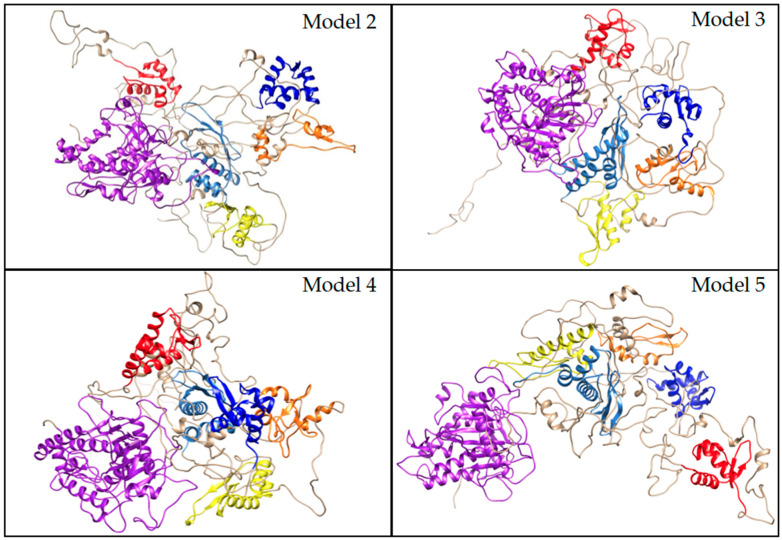
Representative low-energy structures for AlphaFold-included models. The Zα domain, Zβ domain, dsRBD1, dsRBD2, dsRBD3, and the CDD are shown in red, blue, orange, yellow, cornflower blue, and purple, respectively.

Overall, RMSD comparisons highlighted that the lowest-energy structures for each model differed primarily in the interdomain loops and showed minor fluctuations in the ZβBD and dsRBD1 but had highly stable dsRBD3 and CDD structures (Figure 6). Particularly, model 4, which had the lowest overall-energy conformation structures, showed little variation between its top conformations (Figure 6). Examination of the stable and transitional basins for each model revealed that stable basins predominantly captured ordered domain conformations connected by flexible loops, while transitional basins included partial disorder within the β-sheets of the Zα, Zβ, dsRBD1, and dsRBD2 (Figures S16–S19). Collectively, these results reinforce the notion that ADAR1 RNA-binding domains are highly dynamic, enabling adaptability in recognizing diverse substrates, while the dsRBD3 and CDD remain structurally stable to maintain catalytic efficiency and protein-interacting functions. RMSD analysis between the lowest-energy structures of all models further emphasized that the domain arrangements represent distinct but energetically comparable conformations. (Figure S26), supporting a model in which the RNA-binding domains can adopt multiple orientations facilitated by the long interdomain linkers (Figure 7).

## 4. Conclusions

Advances in cryo-EM methodology continue to improve the ability to resolve large proteins and complexes, but the intrinsic disorder and motion of ADAR domains currently remain a barrier to full-length structural determination. Computational modeling provides a valuable complementary approach for assembling structural pieces into cohesive models and probing the dynamics of large, multidomain proteins. In this study, our computational approach complemented traditional structure methods to produce full-length models of the ADAR1p150 isoform and characterize its major dynamics in the apo state, providing insights into both biological mechanisms and translational applications.

Our findings support a framework in which ADAR1 functions are shaped by a division in dynamics, i.e., the dsRBD3 and CDD remain structurally stable, providing a crucial interaction interface and maintaining catalytic efficiency, while the ZBDs and dsRBD1/2 display significant flexibility for facilitating RNA recognition and substrate adaptability. This dynamic architecture is consistent with mechanisms of conformational selection and fly-casting, which allow RNA-binding proteins to scan and engage diverse RNA substrates. Therefore, domain dynamics should be considered along with RNA sequence and shape when considering RNA substrate recognition. Importantly, our simulations also reinforce experimental observations that dsRBD3 serves as a protein-protein interface and indicate the dsRBD3 may influence the stability of the CDD as a possible functional interplay between domains.

These apo models likely display greater flexibility than would be observed in the presence of ligands, RNA substrates, or protein partners, which can stabilize dynamic regions through induced fit, resulting in tighter and more specific interactions [71]. Conformational dynamics of this type cannot be captured by static structures alone and require time-resolved approaches to fully understand ADAR complex formation. In addition, ADAR1 can bind to a multitude of RNA substrates including short RNAs between 15 and 25 bps and siRNAs. The difference in substrate likely results in different domain arrangements which may contribute to substrate selectivity. Future studies that simulate ADAR1 in complex with RNA, protein partners, or small molecule inhibitors will be essential for identifying key interaction interfaces and therapeutic agents, as well as understanding how domains transition into functional conformations with different substrates.

As ADAR1 emerges as a therapeutic target in a variety of fields, understanding its dynamic architecture is essential for identifying novel druggable sites, particularly within flexible or allosteric regions. Integration of computational modeling with emerging high-resolution experimental structures promises to accelerate mechanistic insight into ADAR1 regulation and establish a framework for rational therapeutic design.

In conclusion, while full-length experimental structures of ADAR1 remain elusive, computational modeling provides an essential window into its complex conformational landscape. The interplay between the stable and flexible domains of ADAR1 describes the dynamics of the apo state and lays the groundwork for future simulations of ADAR1 dimers and complexes. Combined with mutagenesis studies, these approaches will help uncover regulatory mechanisms and identify new interfaces for drug development.

## Figures and Tables

**Table 1:** Structural alignment results of AF-P55265-F1 and AlphaFold-included models.

		PDB				
		1QBJ	1XMK	2MDR	9B83	9B89
AF-P55265-F1	TM-score	0.94350	0.79996	0.86573	0.98091	0.84275
	RMSD Value (Å)	0.77	1.84	1.55	1.08	3.07
	Aligned Length	66	71	90	385	422
Model 1	TM-score	0.91895	0.83429	0.74950	0.98121	0.86723
	RMSD Value (Å)	1.40	1.77	1.85	1.13	2.86
	Aligned Length	66	72	81	385	432
Model 2	TM-score	0.91366	0.84227	0.80269	0.97891	0.84385
	RMSD Value (Å)	1.58	1.64	2.54	1.30	3.01
	Aligned Length	66	72	93	385	421
Model 3	TM-score	0.95466	0.81490	0.84983	0.98802	0.85914
	RMSD Value (Å)	0.65	1.79	1.83	0.90	3.06
	Aligned Length	66	71	90	385	430
Model 4	TM-score	0.93571	0.82709	0.84068	0.98879	0.83827
	RMSD Value (Å)	0.79	1.81	2.12	0.92	1.80
	Aligned Length	66	72	91	385	403
Model 5	TM-score	0.94630	0.83280	0.77372	0.98141	0.86549
	RMSD Value (Å)	0.75	1.76	2.54	1.29	3.19
	Aligned Length	66	72	90	385	436

**Table 2:** SAVE’s evaluation of AF-P55265-F1 and AlphaFold-included models.

AlphaFold-included Models Pre-MD Simulation							
**Model**	**Molpdf**	**ERRAT**	**VERIFY3D**	**PROCHECK**			
				**Favored**	**Allowed**	**Generously Allowed**	**Disallowed**
AF-P55265-F1		95.3737	61.42%	78.6%	12.6%	2.4%	6.5%
Model 1	95,629.57031	52.9412	67.58%	82.9%	12.8%	3.6%	0.7%
Model 2	96,981.09375	54.4348	71.23%	82.7%	13.1%	2.6%	1.7%
Model 3	89,784.28125	54.491	71.12%	84.1%	11.9%	2.1%	1.9%
Model 4	85,828.26562	61.7058	68.61%	83.1%	12.4%	2.6%	1.9%
Model 5	92,985.09375	56.4957	67.81%	85.3%	11.3%	2.0%	1.3%
AlphaFold-included Models Post-MD Simulations							
**Model**	**ERRAT**	**VERIFY3D**	**PROCHECK**				
			**Favored**	**Allowed**	**Generously Allowed**		**Disallowed**
Model 2	85.6275	62.56%	88.6%	8.8%	2.1%		0.6%
Model 3	86.9822	63.13%	88.6%	9.3%	1.6%		0.5%
Model 4	88.9872	61.53%	88.0%	10.3%	1.4%		0.3%
Model 5	85.7282	57.65%	87.9%	9.8%	1.6%		0.7%

**Table 3:** Structural alignment results of AlphaFold-included models after MD simulation.

		PDB				
		1QBJ	1XMK	2MDR	9B83	9B89
Model 2	TM-score	0.82375	0.70837	0.64477	0.85329	0.79438
	RMSD Value (Å)	2.03	2.75	3.15	3.22	4.20
	Aligned Length	65	67	93	376	440
Model 3	TM-score	0.79966	0.78393	0.82476	0.89278	0.78305
	RMSD Value (Å)	1.89	1.82	1.87	2.98	4.21
	Aligned Length	65	72	92	382	428
Model 4	TM-score	0.80826	0.74380	0.81220	0.88156	0.77782
	RMSD Value (Å)	1.69	2.24	2.24	3.11	3.92
	Aligned Length	66	70	89	383	422
Model 5	TM-score	0.82185	0.72882	0.73847	0.86625	0.73615
	RMSD Value (Å)	1.77	2.30	2.20	3.16	3.65
	Aligned Length	66	67	84	378	392

## Data Availability

The data presented in this study are openly available at https://github.com/WAMillerLab/ADAR/tree/ADAR1 (accessed on 18 October 2025).

## Supplementary Materials

The following supporting information can be downloaded at: https://www.mdpi.com/article/10.3390/cimb47110958/s1.

## Abbreviations

MD, Molecular Dynamics; PCA, Principal Component Analysis; ZDB, Z-DNA Binding Domains; CDD, Catalytic Deaminase Domain; NMR, Nuclear Magnetic Resonance Spectroscopy; cryo-EM, Cryogenic Electron Microscropy; dsRBD, double-stranded RNA-Binding Domains; RCSB PDB, Research Collaboratory for Structural Bioinformatics Protein Data Bank; Cα-RMSD, Alpha-Carbon Root Mean.
