# Supplementary material for: In Silico Characterization of ADAR1: Structure, Dynamics, and Functional Implications
Source: Curr Issues Mol Biol. 2025 Nov 18;47(11):958. doi: 10.3390/cimb47110958 (PMC12651035; doi:10.3390/cimb47110958)
Supplement: Supplementary file 1 [file cimb-47-00958-s001.zip › Supporting_Information_Figures_ADAR1_S_11-13-25.pdf]

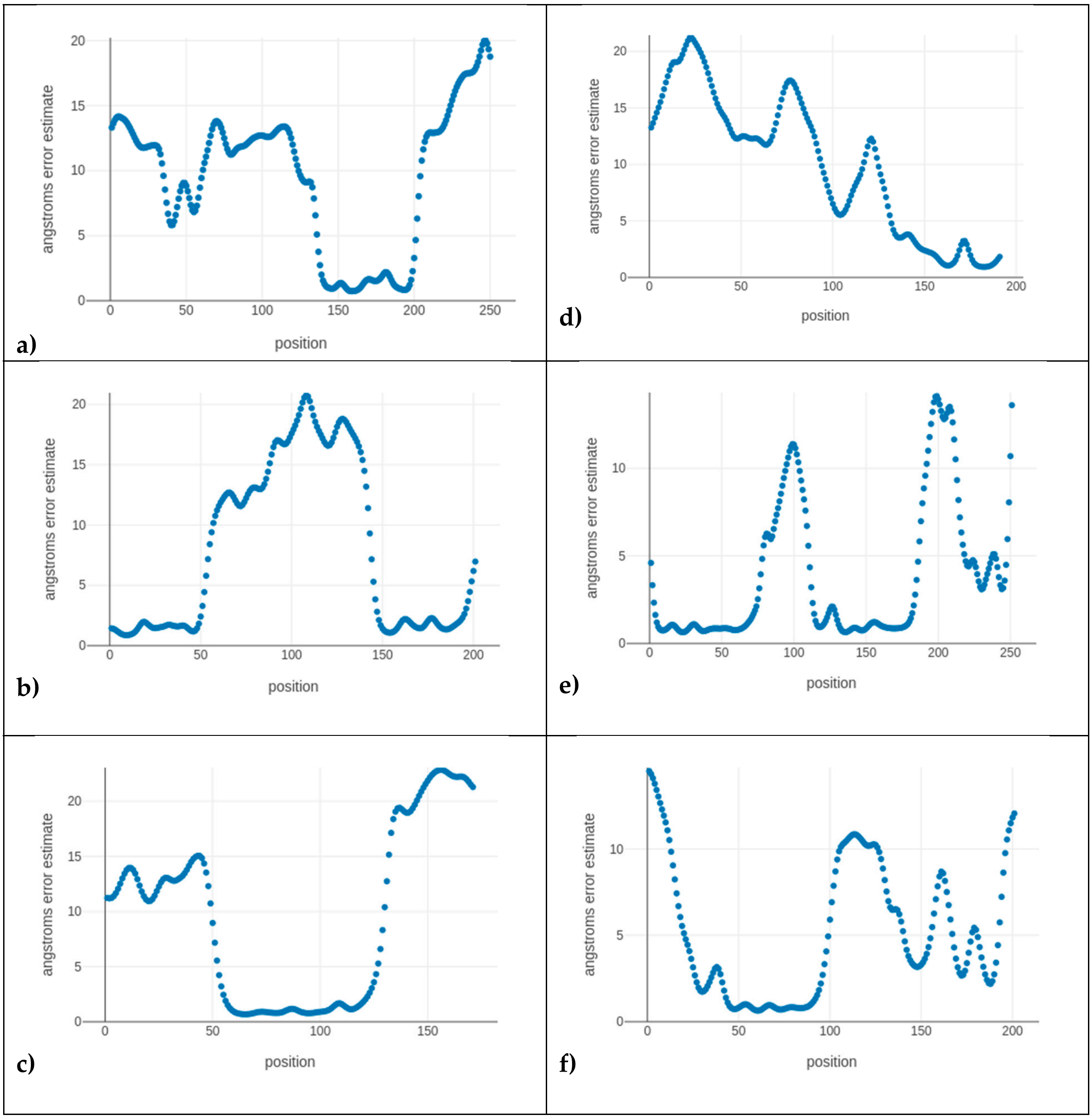

Figure S1. From RosettaCM, model 1 plots of the estimated error in Å for each residue position across the residue ranges **a)** 1–250, **b)** 150–350, **c)** 240–410, **d)** 360–550, **e)** 500–750, and **f)** 700–900.

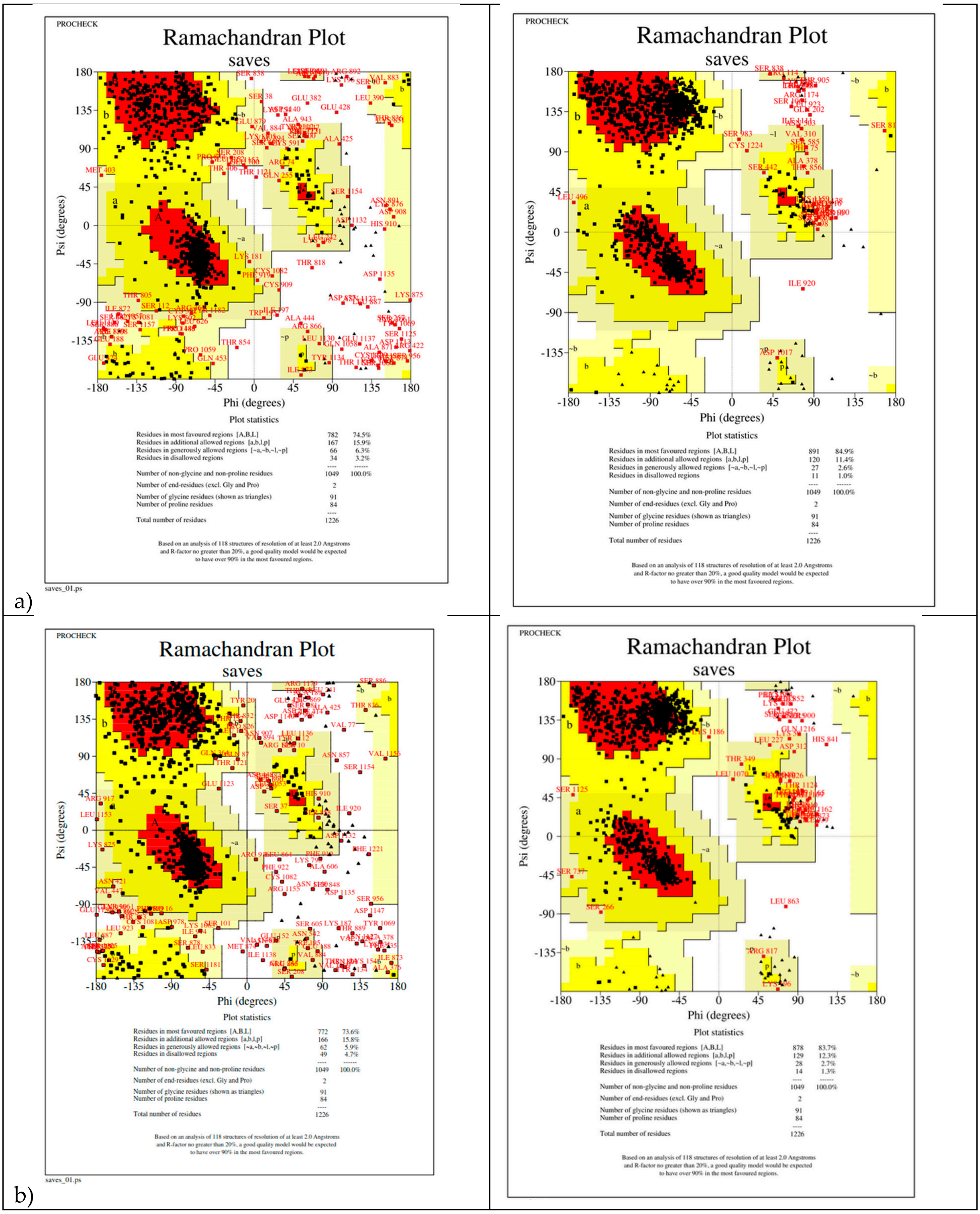

PROCHECK
Ramachandran Plot
saves
Psi (degrees)
Phi (degrees)
Plot statistics
Residues in most favoured regions [A,B,L] 782 74.5%
Residues in additional allowed regions [a,b,l,p] 167 15.9%
Residues in generously allowed regions [~a,~b,~l,~p] 66 6.3%
Residues in disallowed regions 34 3.2%
Number of non-glycine and non-proline residues 1049 100.0%
Number of end-residues (excl. Gly and Pro) 2
Number of glycine residues (shown as triangles) 91
Number of proline residues 84
Total number of residues 1226
Based on an analysis of 118 structures of resolution of at least 2.0 Angstroms and R-factor no greater than 20%, a good quality model would be expected to have over 90% in the most favoured regions.
saves_01.ps
a)
PROCHECK
Ramachandran Plot
saves
Residues in most favoured regions [A,B,L] 891 84.9%
Residues in additional allowed regions [a,b,l,p] 120 11.4%
Residues in generously allowed regions [~a,~b,~l,~p] 27 2.6%
Residues in disallowed regions 11 1.0%
Number of non-glycine and non-proline residues 1049 100.0%
Total number of residues 1226
PROCHECK
Ramachandran Plot
saves
Residues in most favoured regions [A,B,L] 772 73.6%
Residues in additional allowed regions [a,b,l,p] 166 15.8%
Residues in generously allowed regions [~a,~b,~l,~p] 62 5.9%
Residues in disallowed regions 49 4.7%
Number of non-glycine and non-proline residues 1049 100.0%
Total number of residues 1226
saves_01.ps
b)
PROCHECK
Ramachandran Plot
saves
Residues in most favoured regions [A,B,L] 878 83.7%
Residues in additional allowed regions [a,b,l,p] 129 12.3%
Residues in generously allowed regions [~a,~b,~l,~p] 28 2.7%
Residues in disallowed regions 14 1.3%
Number of non-glycine and non-proline residues 1049 100.0%
Total number of residues 1226

c)

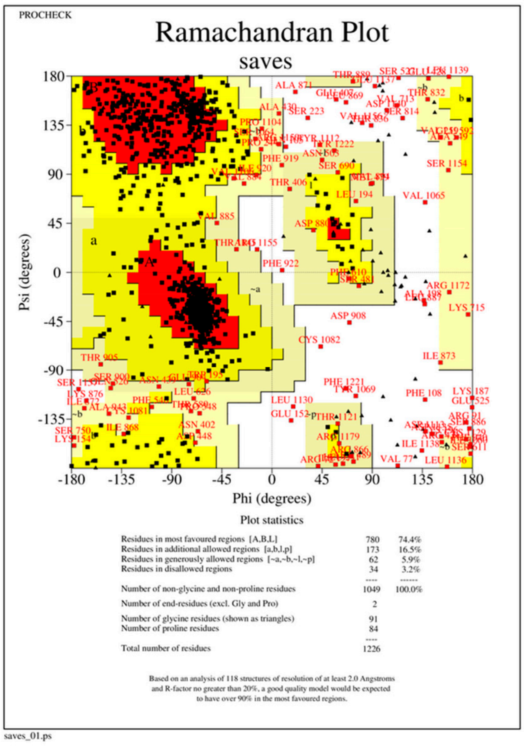
PROCHECK
Ramachandran Plot
saves
Psi (degrees)
Phi (degrees)
Plot statistics
Residues in most favoured regions [A,B,L] 780 74.4%
Residues in additional allowed regions [a,b,l,p] 173 16.5%
Residues in generously allowed regions [~a,~b,~l,~p] 62 5.9%
Residues in disallowed regions 34 3.2%
Number of non-glycine and non-proline residues 1049 100.0%
Number of end-residues (excl. Gly and Pro) 2
Number of glycine residues (shown as triangles) 91
Number of proline residues 84
Total number of residues 1226
Based on an analysis of 118 structures of resolution of at least 2.0 Angstroms and R-factor no greater than 20%, a good quality model would be expected to have over 90% in the most favoured regions.
saves_01.ps

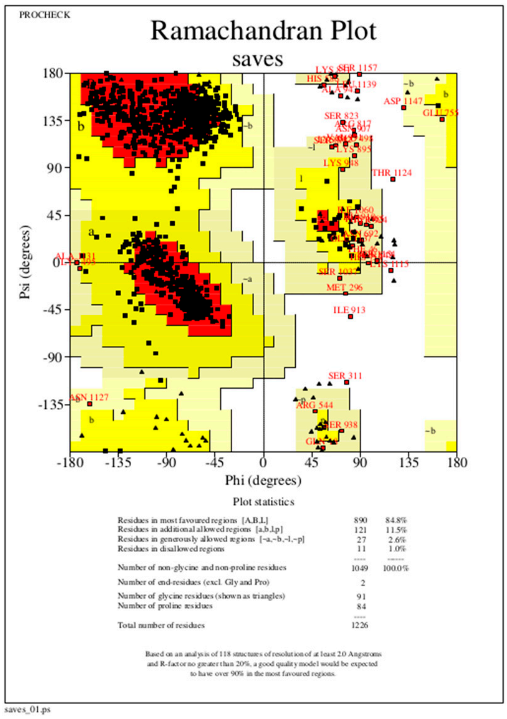
PROCHECK
Ramachandran Plot
saves
Psi (degrees)
Phi (degrees)
Plot statistics
Residues in most favoured regions [A,B,L] 890 84.8%
Residues in additional allowed regions [a,b,l,p] 121 11.5%
Residues in generously allowed regions [~a,~b,~l,~p] 27 2.6%
Residues in disallowed regions 11 1.0%
Number of non-glycine and non-proline residues 1049 100.0%
Number of end-residues (excl. Gly and Pro) 2
Number of glycine residues (shown as triangles) 91
Number of proline residues 84
Total number of residues 1226
Based on an analysis of 118 structures of resolution of at least 2.0 Angstroms and R-factor no greater than 20%, a good quality model would be expected to have over 90% in the most favoured regions.
saves_01.ps

d)

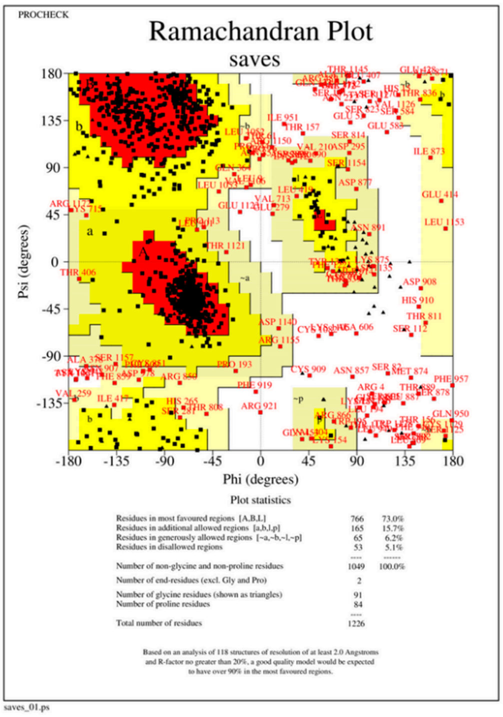
PROCHECK
Ramachandran Plot
saves
Psi (degrees)
Phi (degrees)
Plot statistics
Residues in most favoured regions [A,B,L] 766 73.0%
Residues in additional allowed regions [a,b,l,p] 165 15.7%
Residues in generously allowed regions [~a,~b,~l,~p] 65 6.2%
Residues in disallowed regions 53 5.1%
Number of non-glycine and non-proline residues 1049 100.0%
Number of end-residues (excl. Gly and Pro) 2
Number of glycine residues (shown as triangles) 91
Number of proline residues 84
Total number of residues 1226
Based on an analysis of 118 structures of resolution of at least 2.0 Angstroms and R-factor no greater than 20%, a good quality model would be expected to have over 90% in the most favoured regions.
saves_01.ps

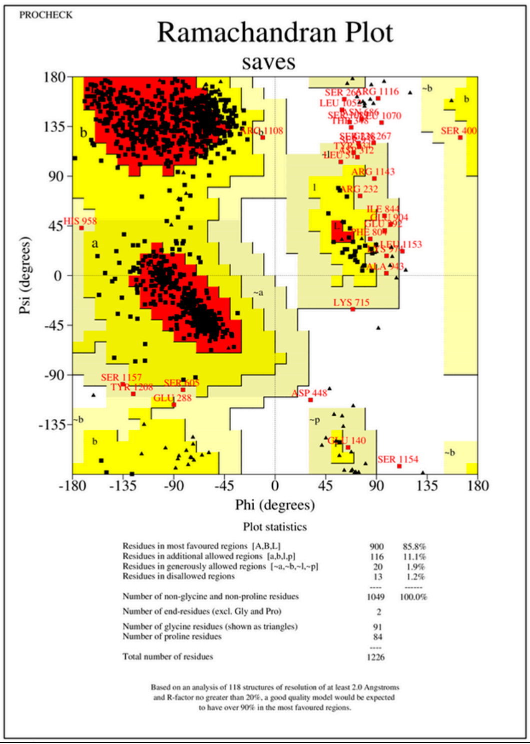
PROCHECK
Ramachandran Plot
saves
Psi (degrees)
Phi (degrees)
Plot statistics
Residues in most favoured regions [A,B,L] 900 85.8%
Residues in additional allowed regions [a,b,l,p] 116 11.1%
Residues in generously allowed regions [~a,~b,~l,~p] 20 1.9%
Residues in disallowed regions 13 1.2%
Number of non-glycine and non-proline residues 1049 100.0%
Number of end-residues (excl. Gly and Pro) 2
Number of glycine residues (shown as triangles) 91
Number of proline residues 84
Total number of residues 1226
Based on an analysis of 118 structures of resolution of at least 2.0 Angstroms and R-factor no greater than 20%, a good quality model would be expected to have over 90% in the most favoured regions.

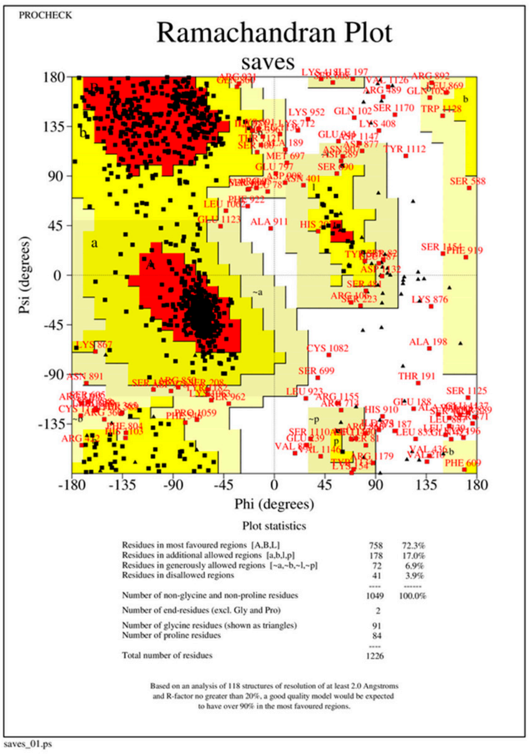

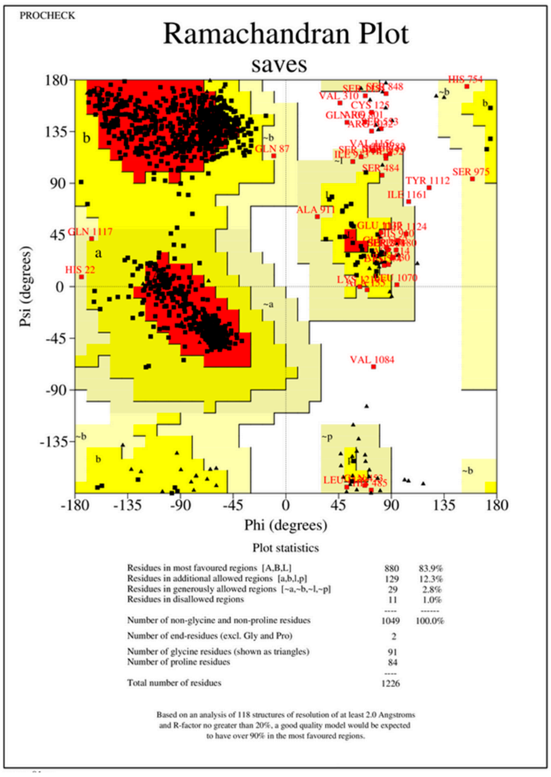

e)

Figure S2. a-e) Ramachandran plots generated by PROCHECK of initial models 1-5 prior to MD simulation (on left) and after MD simulation (on right). Favored regions (A, B, and L) in red, additional allowed regions (a, b, l, and p) in yellow, generously allowed regions (~a, ~b, ~l, and ~p) in tan, and disallowed regions in white. Squares represent non-glycine residues, and triangles represent non-end glycine residues.

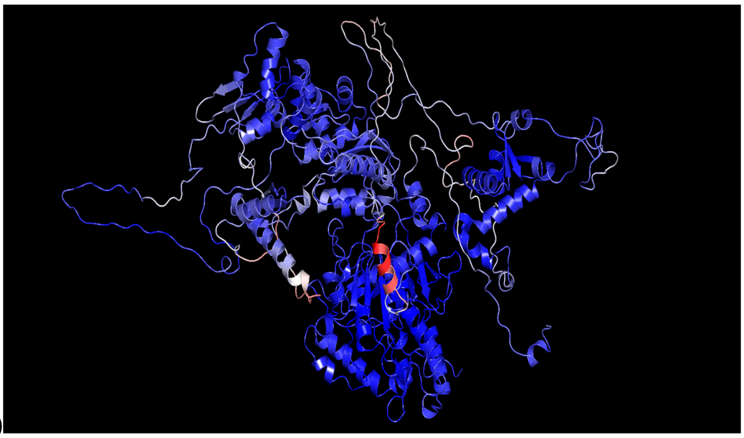

A)

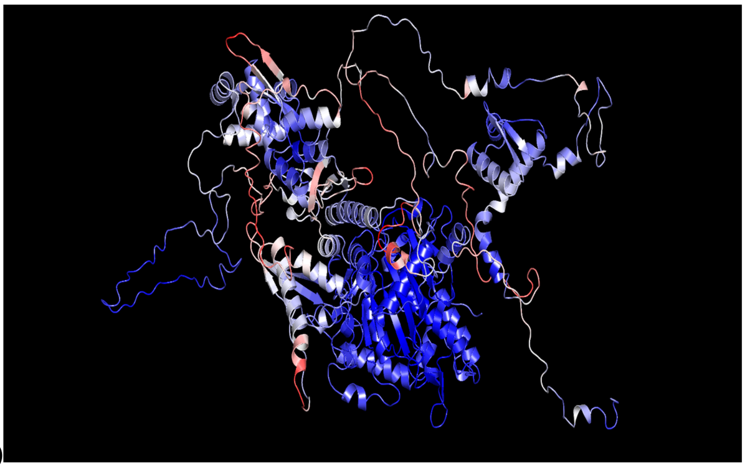

B)

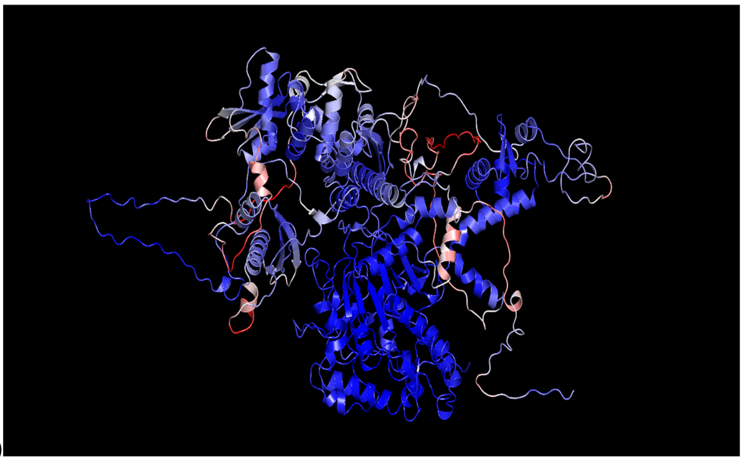

C)

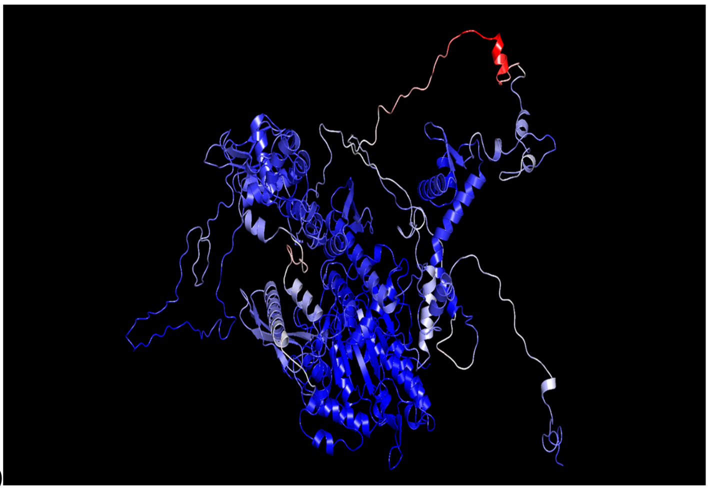

D)

E) 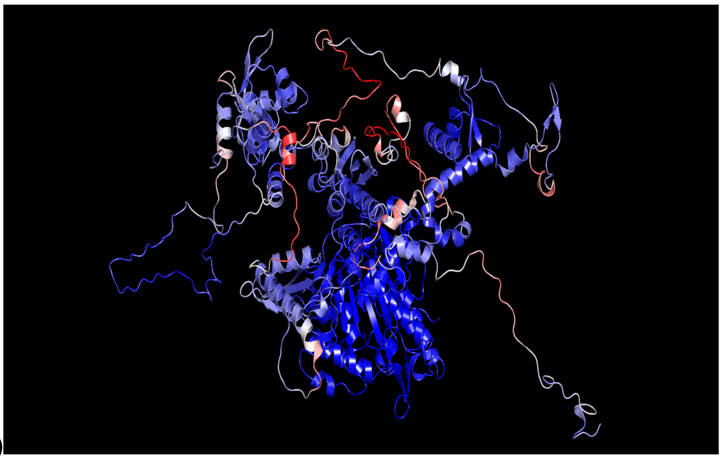

Figure S3. RMSD comparison between AlphaFold-included models (A-E). Color spectra spans from blue, to white, to red, where blue represents areas that stay conserved and red represents areas with the highest structural variation.

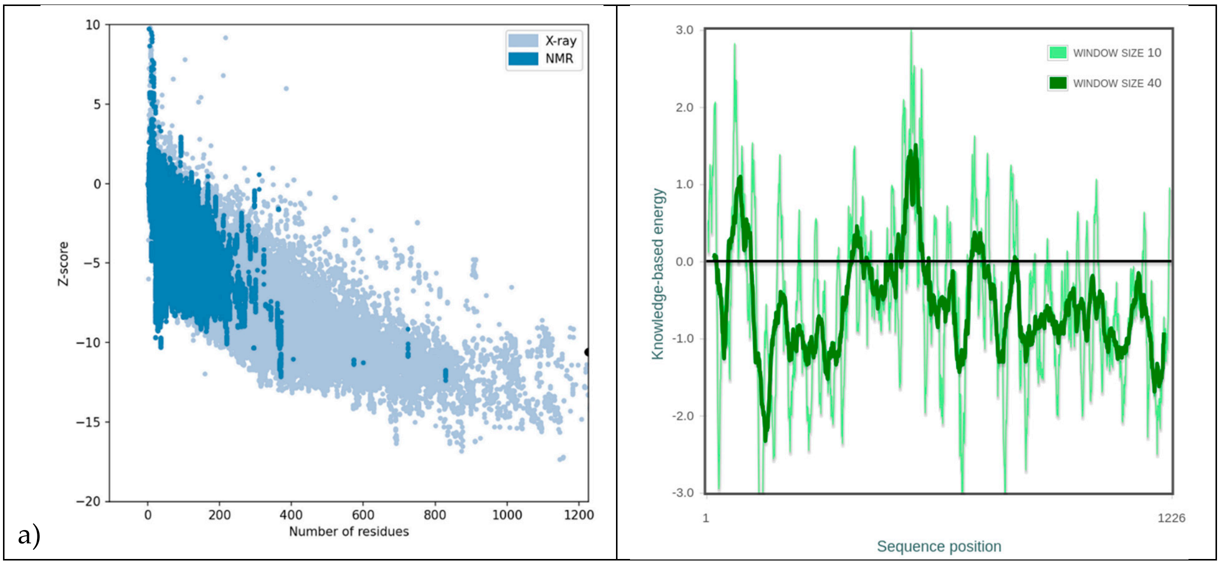

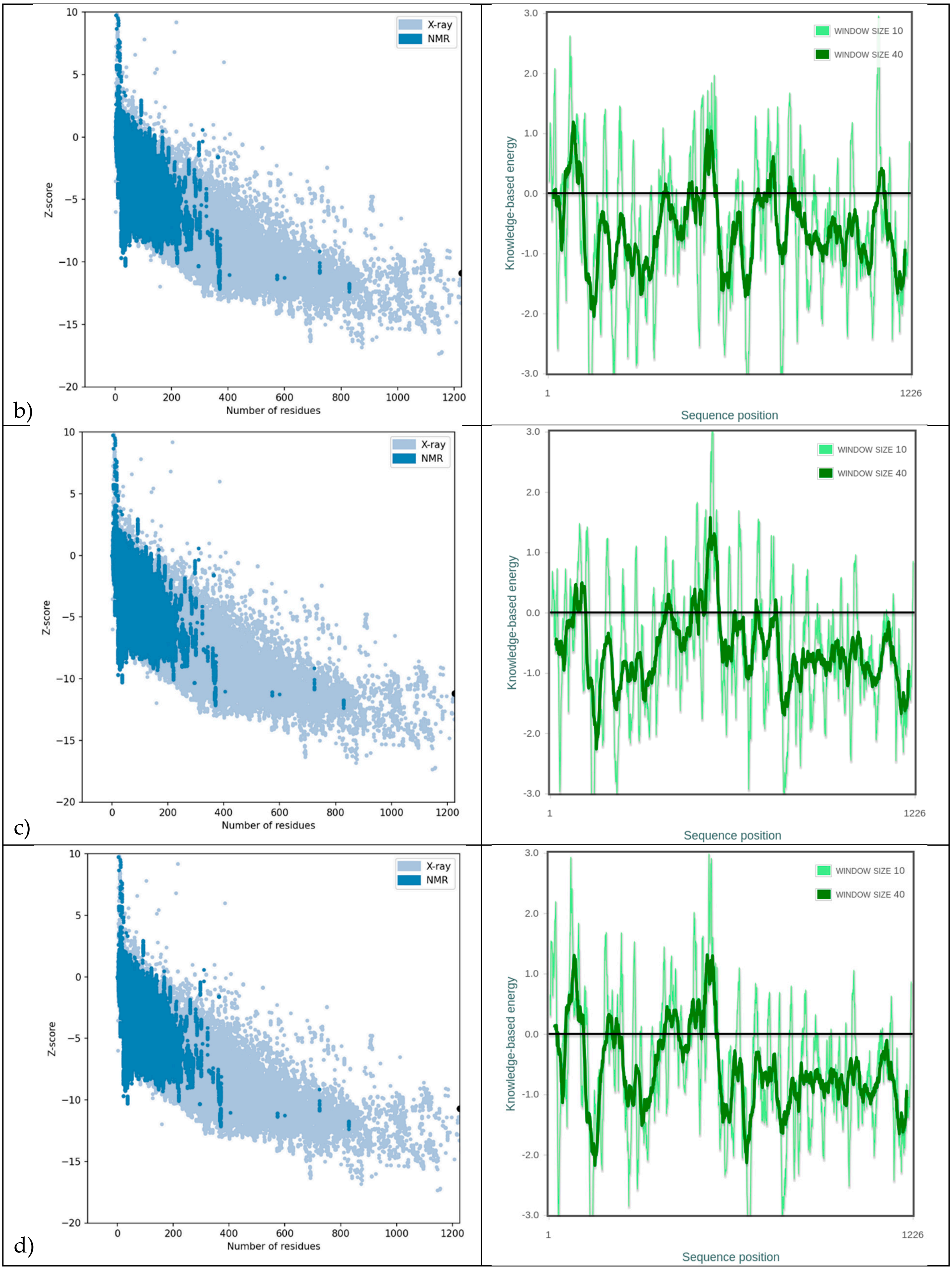

X-ray
NMR
Z-score
Number of residues
WINDOW SIZE 10
WINDOW SIZE 40
Knowledge-based energy
Sequence position
1
1226
b)
c)
d)

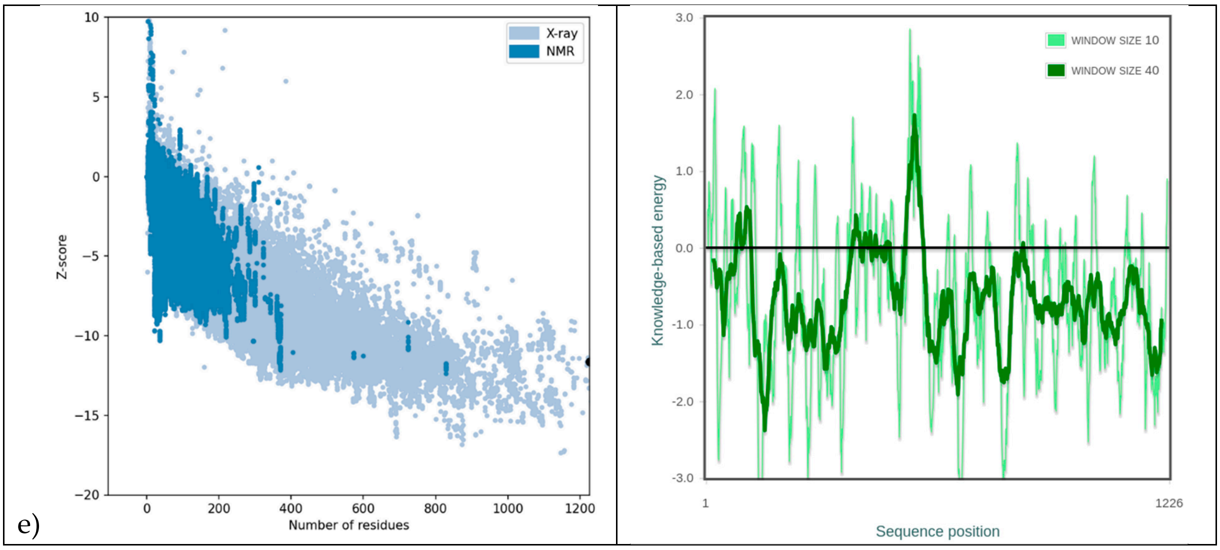

Figure S4. ProSA results for AlphaFold included models. For each model 1-5 labeled a-e, the left is the overall model quality plots, and the right is the local model quality plots.

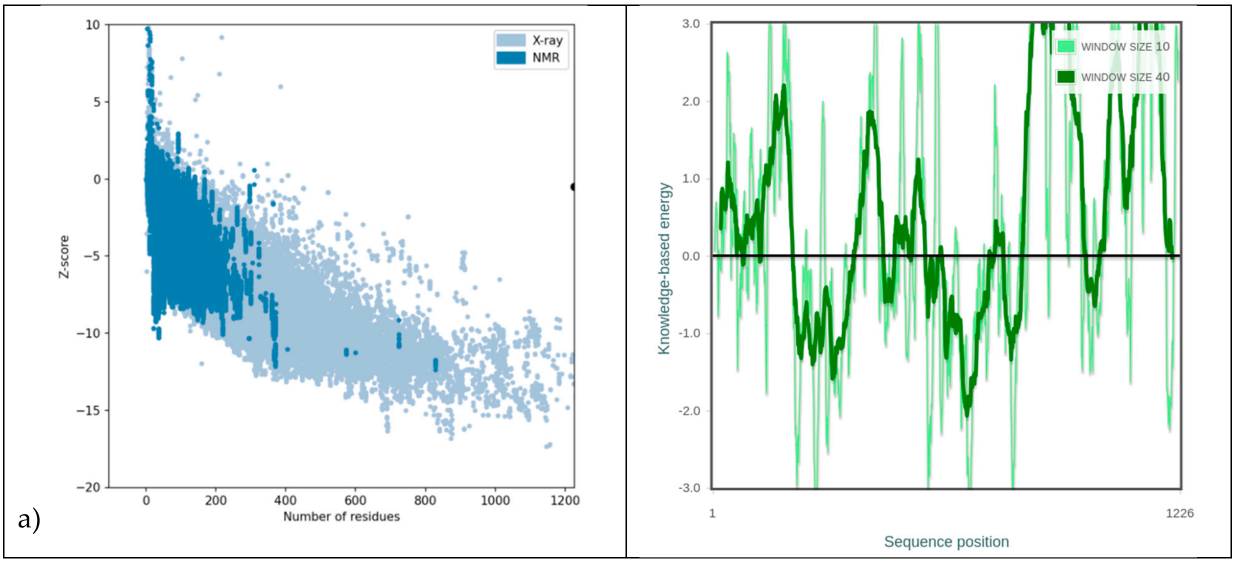

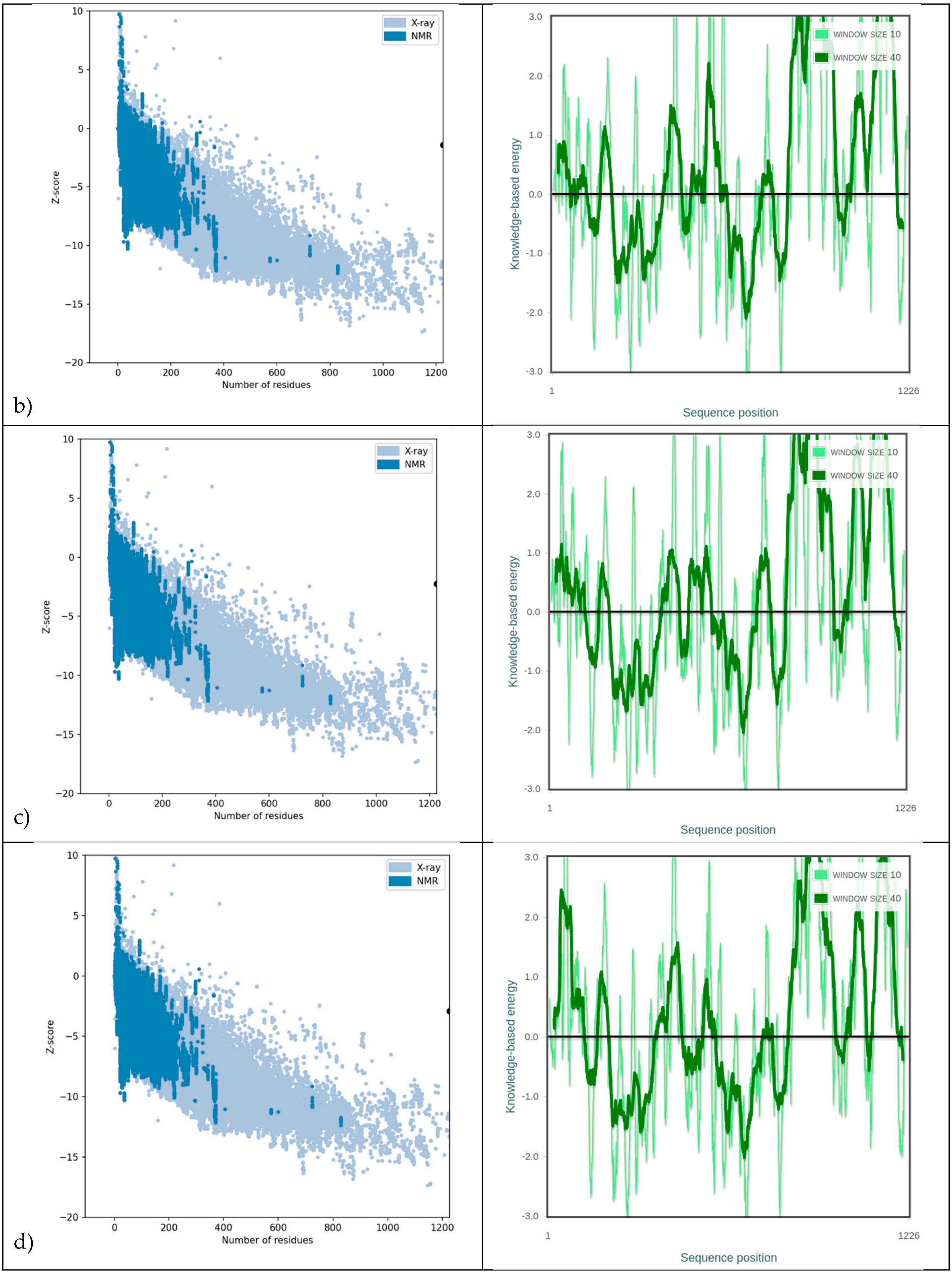
X-ray
NMR
Z-score
Number of residues
b)
WINDOW SIZE 10
WINDOW SIZE 40
Knowledge-based energy
Sequence position
1
1226
X-ray
NMR
Z-score
Number of residues
c)
WINDOW SIZE 10
WINDOW SIZE 40
Knowledge-based energy
Sequence position
1
1226
X-ray
NMR
Z-score
Number of residues
d)
WINDOW SIZE 10
WINDOW SIZE 40
Knowledge-based energy
Sequence position
1
1226

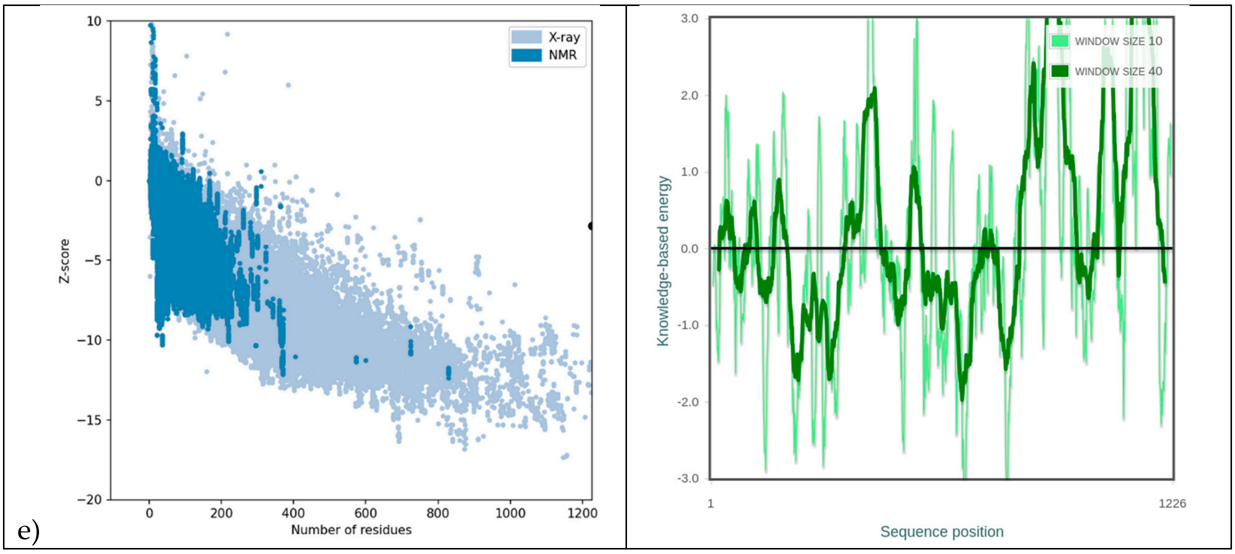

e)

Figure S5. ProSA results for initial homology models. For each model 1-5 labeled a-e, the left is the overall model quality plot and the right is the local model quality plots.

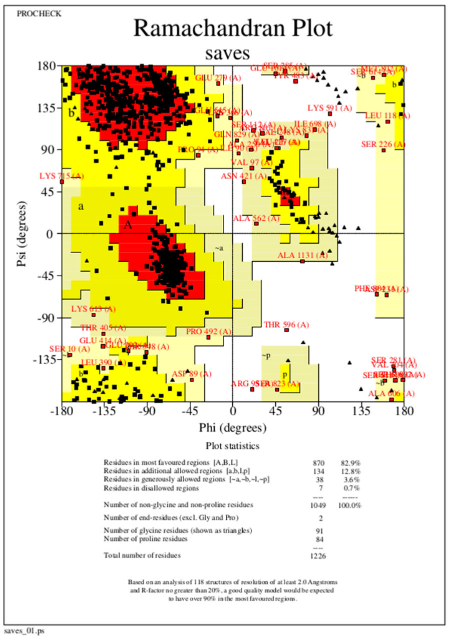

a)

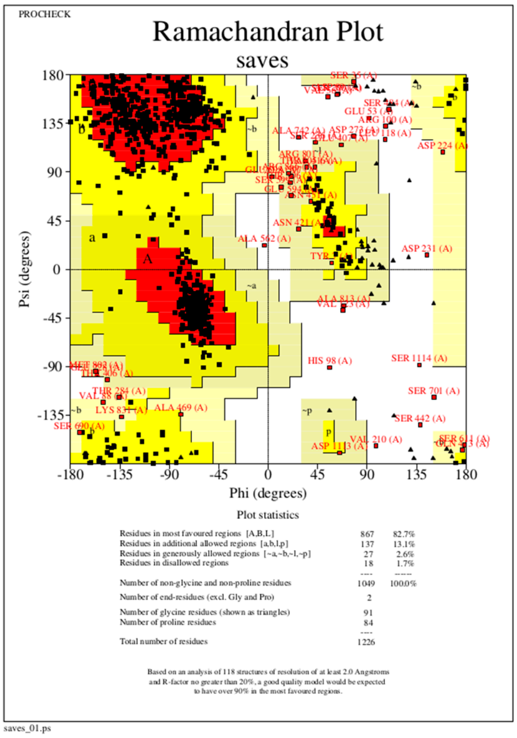
PROCHECK
Ramachandran Plot
saves
Psi (degrees)
Phi (degrees)
Plot statistics
Residues in most favoured regions [A,B,L] 867 82.7%
Residues in additional allowed regions [a,b,l,p] 137 13.1%
Residues in generously allowed regions [~a,~b,~l,~p] 27 2.6%
Residues in disallowed regions 18 1.7%
Number of non-glycine and non-proline residues 1049 100.0%
Number of end-residues (excl. Gly and Pro) 2
Number of glycine residues (shown as triangles) 91
Number of proline residues 84
Total number of residues 1226
Based on an analysis of 118 structures of resolution of at least 2.0 Angstroms and R-factor no greater than 20%, a good quality model would be expected to have over 90% in the most favoured regions.
saves_01.ps

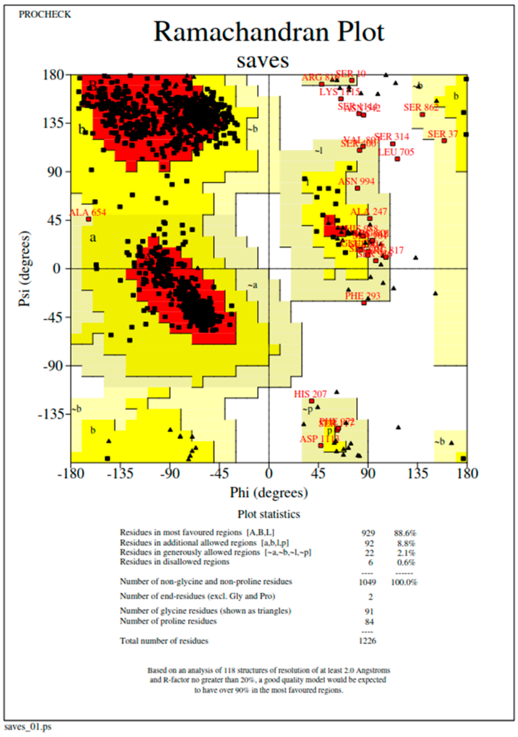
PROCHECK
Ramachandran Plot
saves
Psi (degrees)
Phi (degrees)
Plot statistics
Residues in most favoured regions [A,B,L] 929 88.6%
Residues in additional allowed regions [a,b,l,p] 92 8.8%
Residues in generously allowed regions [~a,~b,~l,~p] 22 2.1%
Residues in disallowed regions 6 0.6%
Number of non-glycine and non-proline residues 1049 100.0%
Number of end-residues (excl. Gly and Pro) 2
Number of glycine residues (shown as triangles) 91
Number of proline residues 84
Total number of residues 1226
Based on an analysis of 118 structures of resolution of at least 2.0 Angstroms and R-factor no greater than 20%, a good quality model would be expected to have over 90% in the most favoured regions.
saves_01.ps

b)

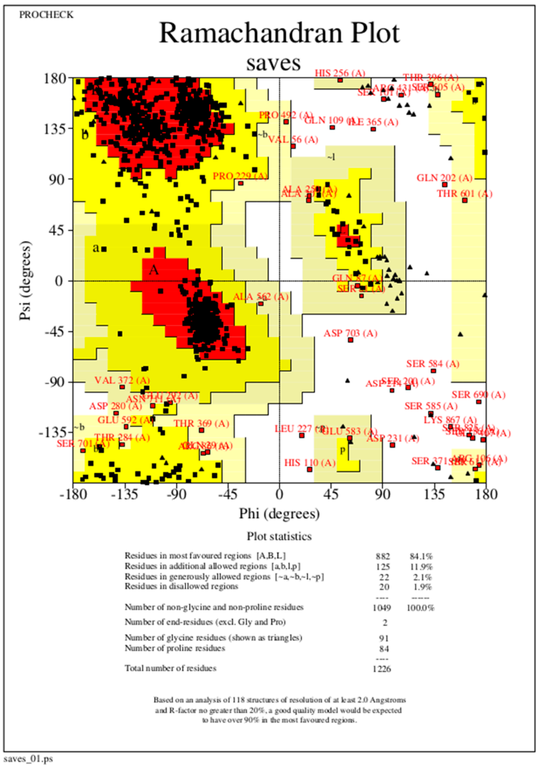
PROCHECK
Ramachandran Plot
saves
Psi (degrees)
Phi (degrees)
Plot statistics
Residues in most favoured regions [A,B,L] 882 84.1%
Residues in additional allowed regions [a,b,l,p] 125 11.9%
Residues in generously allowed regions [~a,~b,~l,~p] 22 2.1%
Residues in disallowed regions 20 1.9%
Number of non-glycine and non-proline residues 1049 100.0%
Number of end-residues (excl. Gly and Pro) 2
Number of glycine residues (shown as triangles) 91
Number of proline residues 84
Total number of residues 1226
Based on an analysis of 118 structures of resolution of at least 2.0 Angstroms and R-factor no greater than 20%, a good quality model would be expected to have over 90% in the most favoured regions.
saves_01.ps

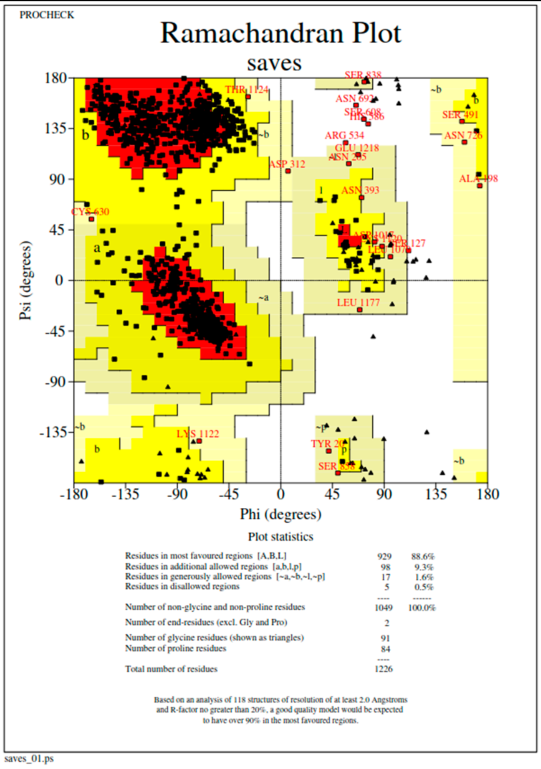
PROCHECK
Ramachandran Plot
saves
Psi (degrees)
Phi (degrees)
Plot statistics
Residues in most favoured regions [A,B,L] 929 88.6%
Residues in additional allowed regions [a,b,l,p] 98 9.3%
Residues in generously allowed regions [~a,~b,~l,~p] 17 1.6%
Residues in disallowed regions 5 0.5%
Number of non-glycine and non-proline residues 1049 100.0%
Number of end-residues (excl. Gly and Pro) 2
Number of glycine residues (shown as triangles) 91
Number of proline residues 84
Total number of residues 1226
Based on an analysis of 118 structures of resolution of at least 2.0 Angstroms and R-factor no greater than 20%, a good quality model would be expected to have over 90% in the most favoured regions.
saves_01.ps

c)

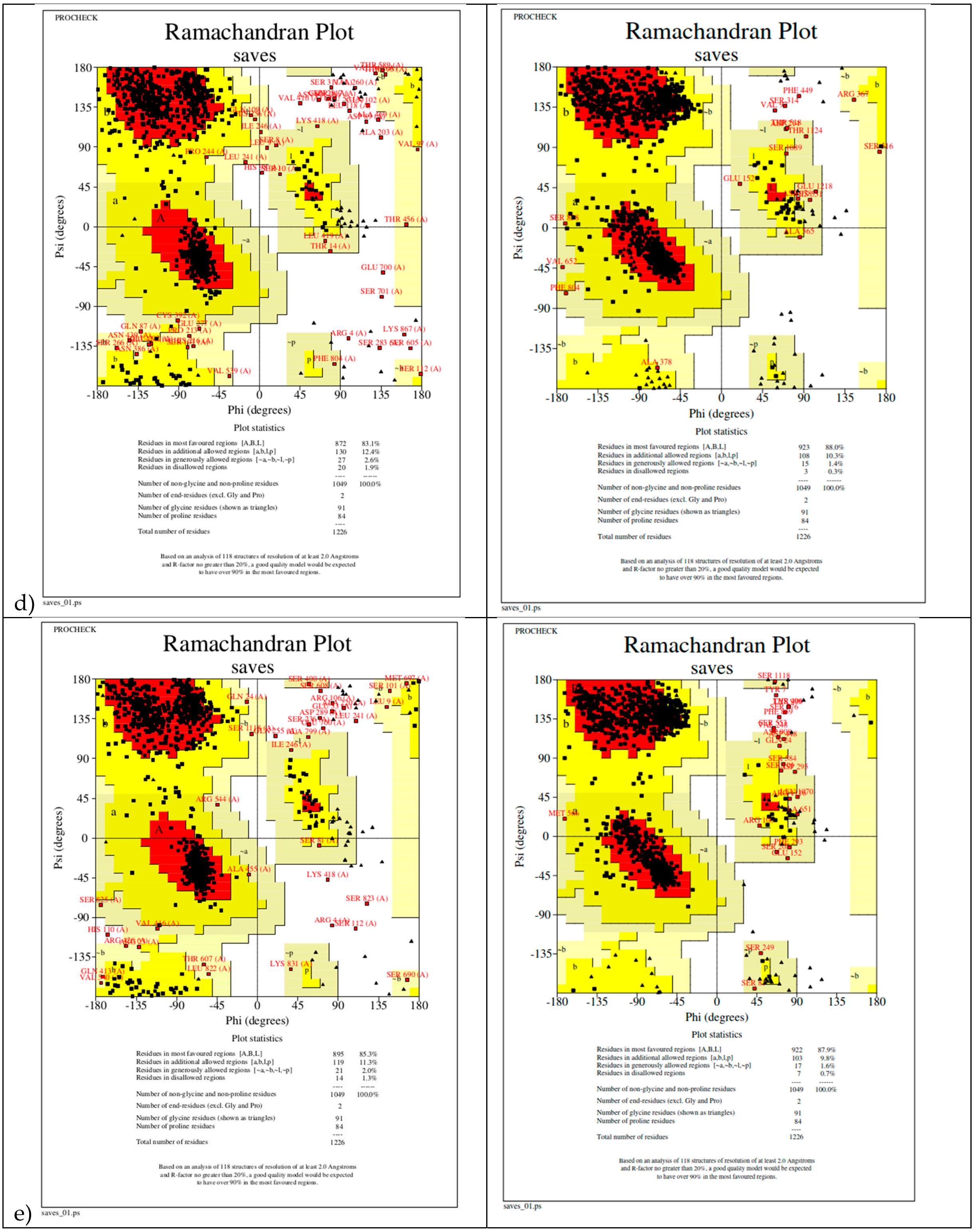

Figure S6. a-e) Ramachandran plots generated by PROCHECK of AlphaFold included models 1-5 prior to MD simulation (on left) and model 2-5 after MD simulation (on right). Favored regions (A, B, and L) in red, additional allowed regions (a, b, l, and p) in yellow, generously

allowed regions (~a, ~b, ~l, and ~p) in tan, and disallowed regions in white. Squares represent non-glycine residues, and triangles represent non-end glycine residues.

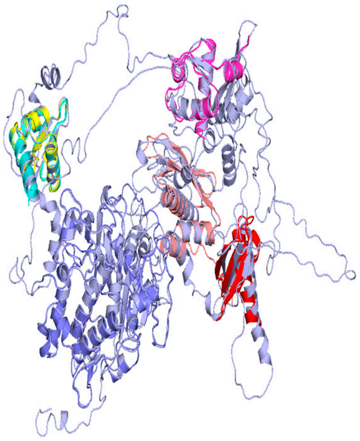

a)

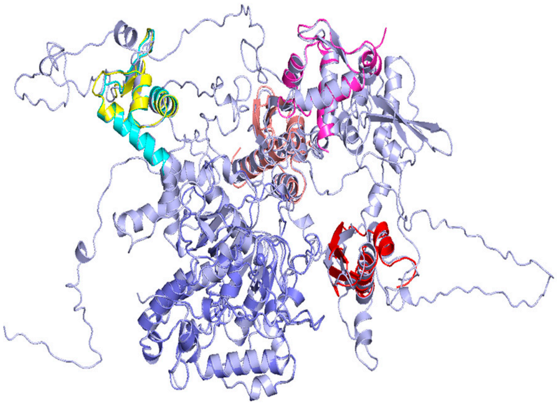

b)

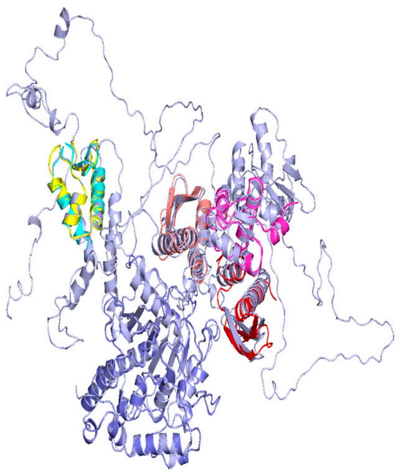

c)

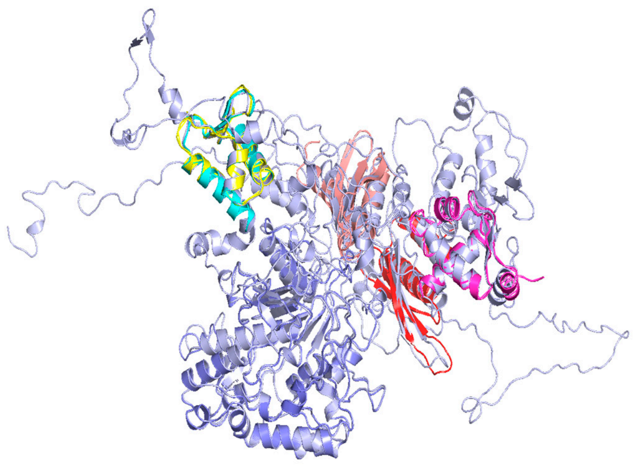

d)

Figure S7. Overlay of PDB templates: 1QBJ (cyan), 1XMK (pink), 2MDR (salmon), 9B83 (slate), 2B7T (red) onto AlphaFold-included models 2-5, a-d.

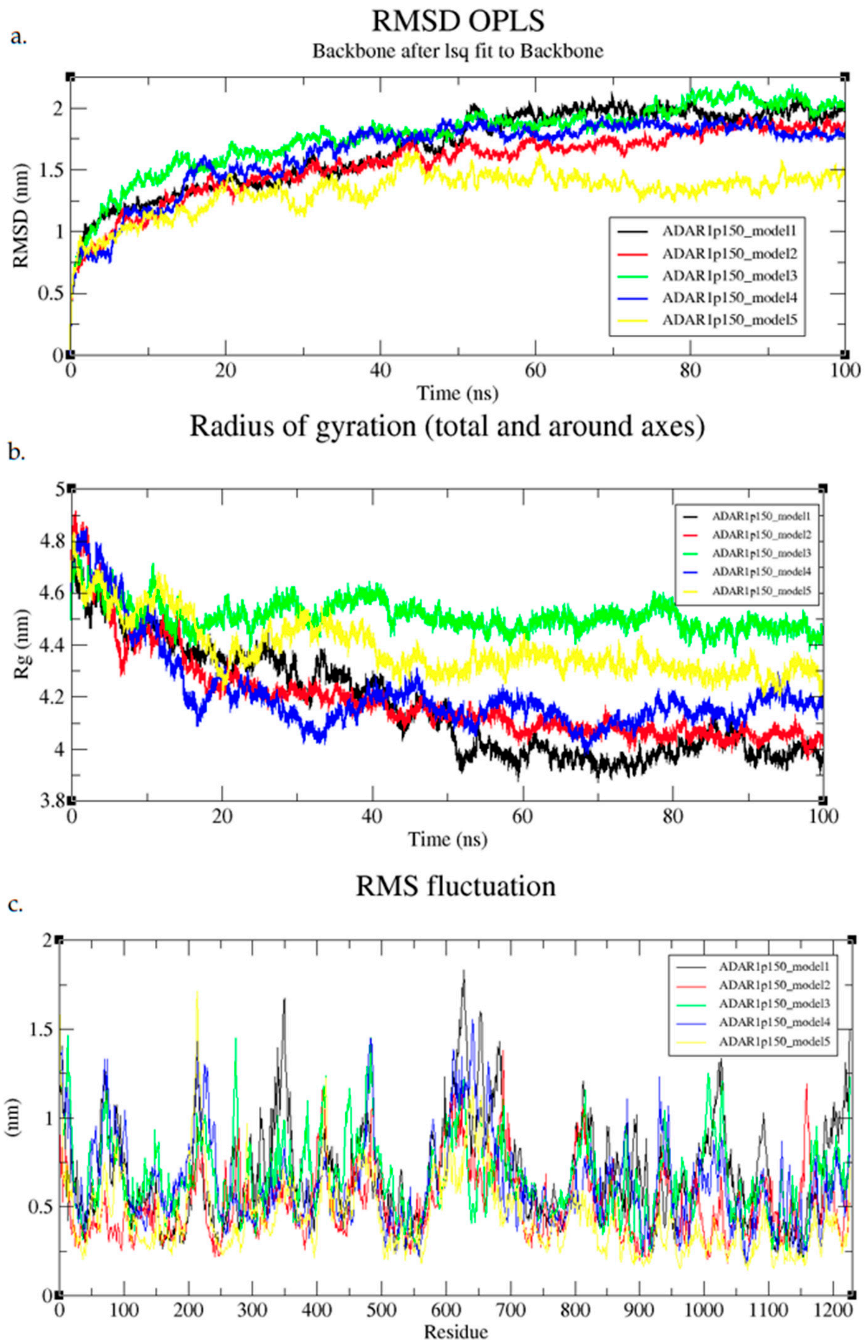

Figure S8. a) RMSD, b)Rg, c) RMSF plots of ADAR1 full-length initial models during initial 100 ns MD simulations.

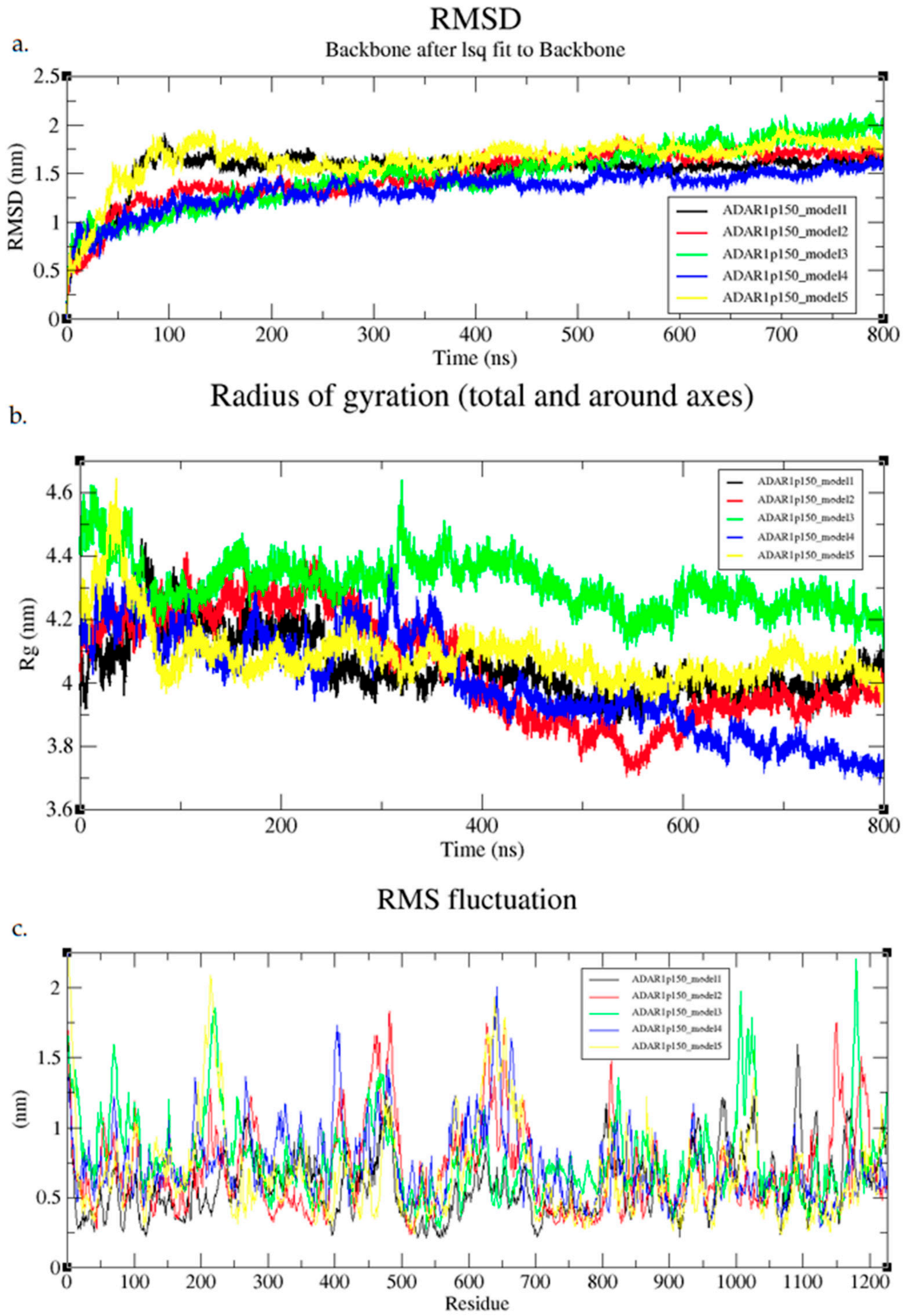

Figure S9. a) RMSD, b)Rg, c) RMSF plots of ADAR1 full-length initial models during 800 ns MD simulations.

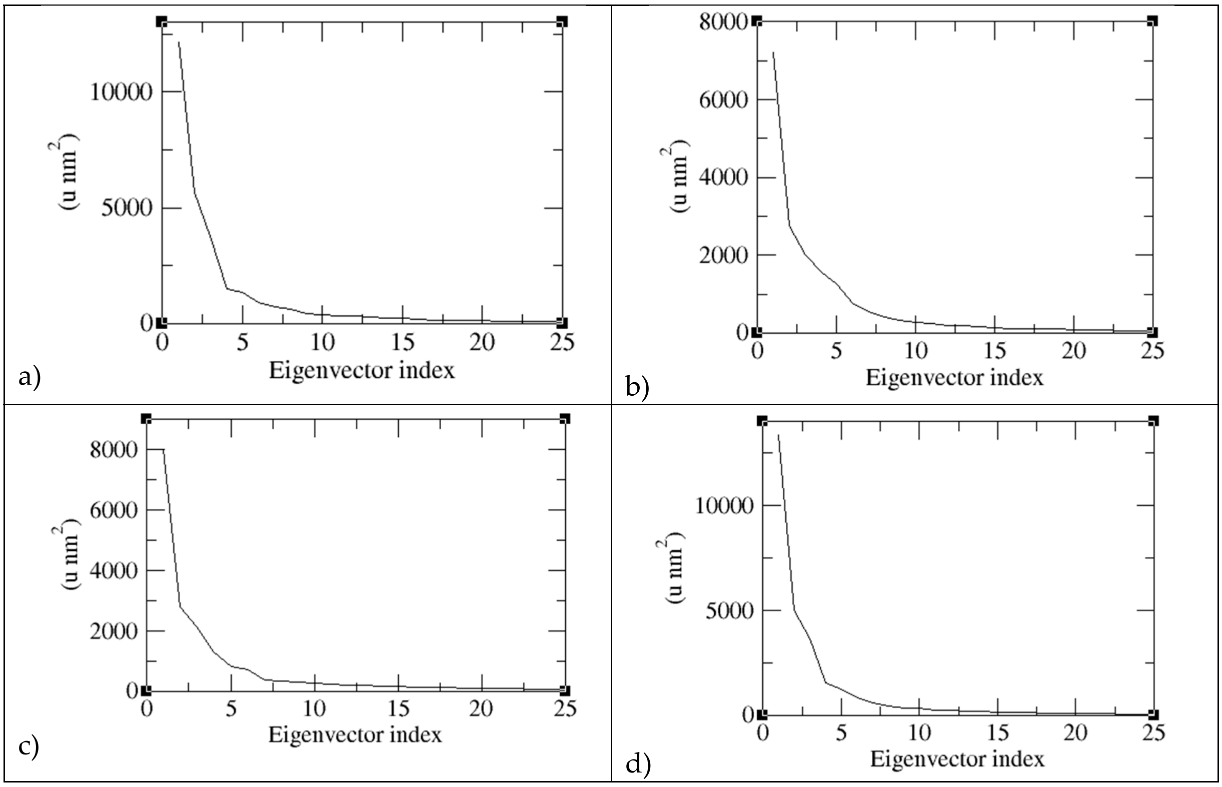

Figure S10. Scree plots for models a) 2, b) 3, c) 4, and d) 5 of the AlphaFold included models.

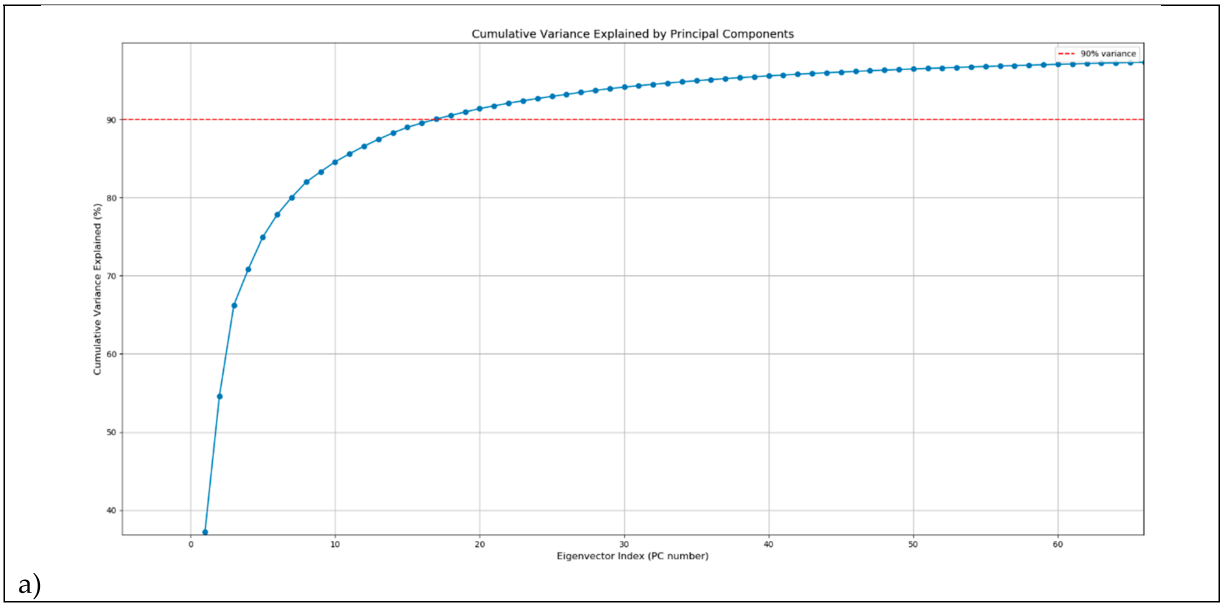

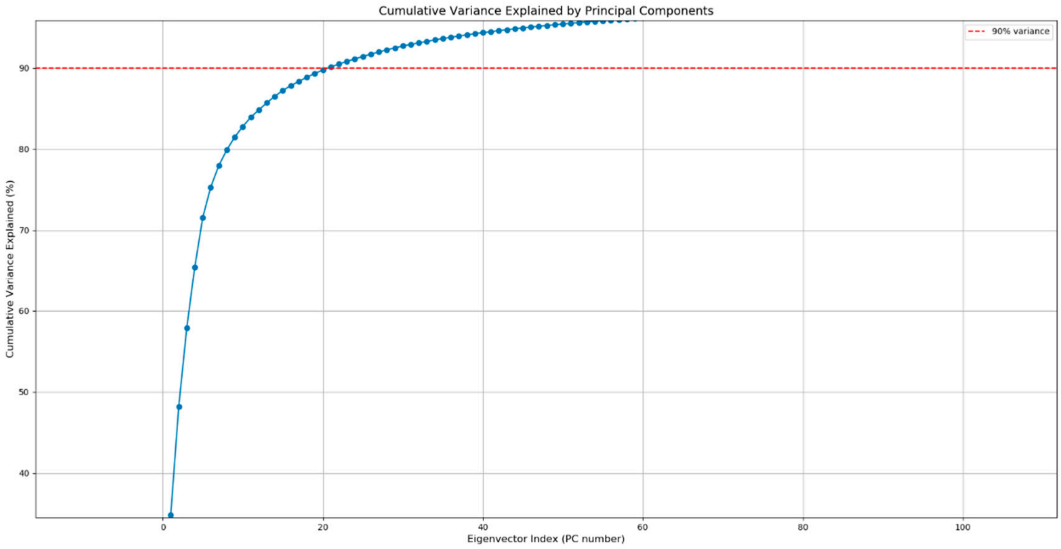
Cumulative Variance Explained by Principal Components
90% variance
Cumulative Variance Explained (%)
Eigenvector Index (PC number)
40
50
60
70
80
90
0
20
40
60
80
100

b)

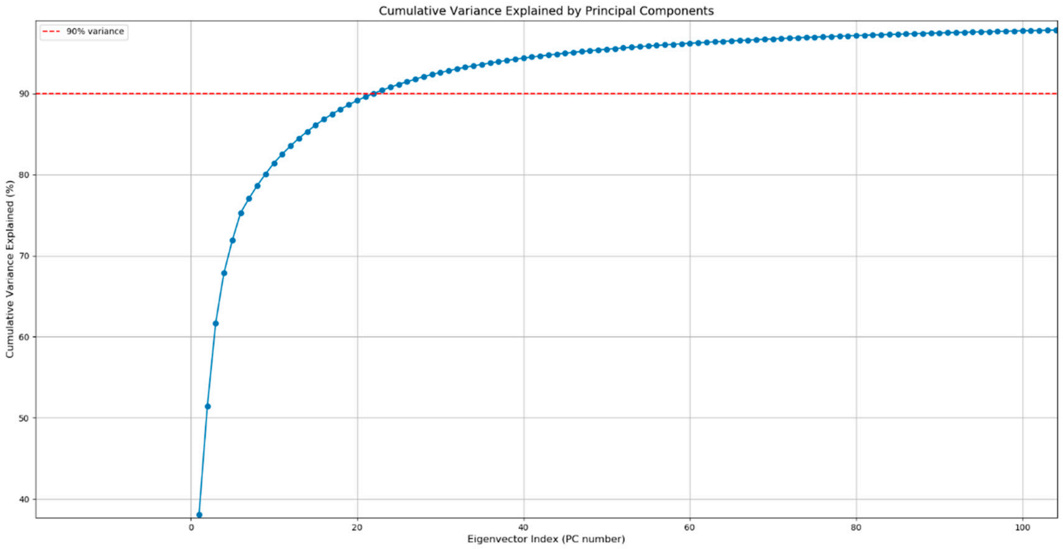
Cumulative Variance Explained by Principal Components
90% variance
Cumulative Variance Explained (%)
Eigenvector Index (PC number)
40
50
60
70
80
90
0
20
40
60
80
100

c)

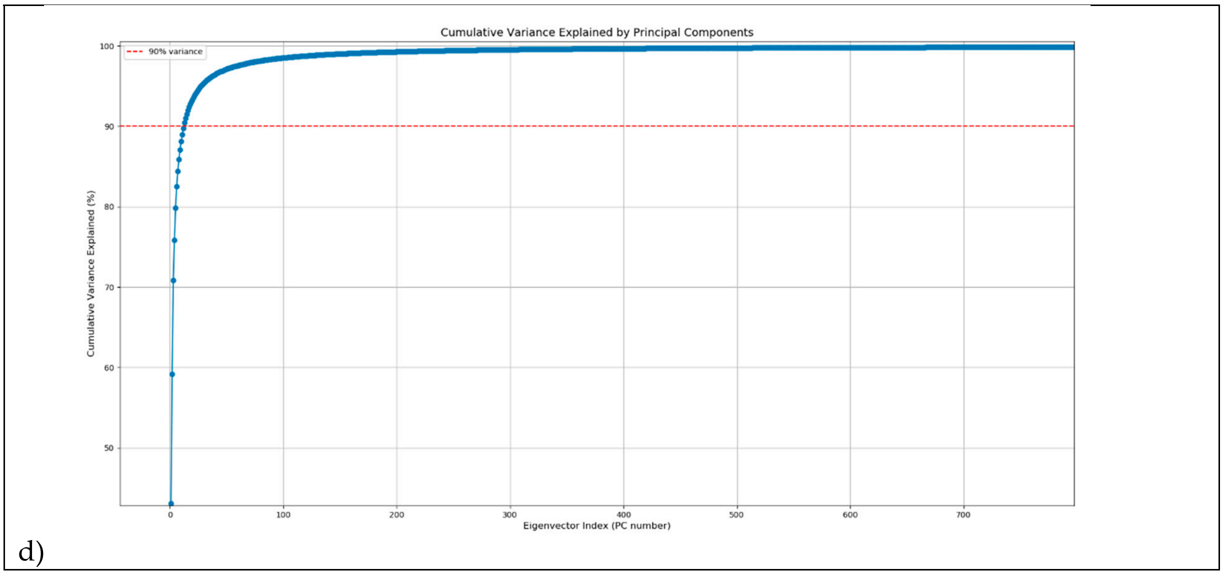

d)

Figure S11. Cumulative variance plots for models a) 2, b) 3, c) 4, and d) 5 of the AlphaFold included models. The dashed red line represents the cut off for 90% of cumulative variance explained.

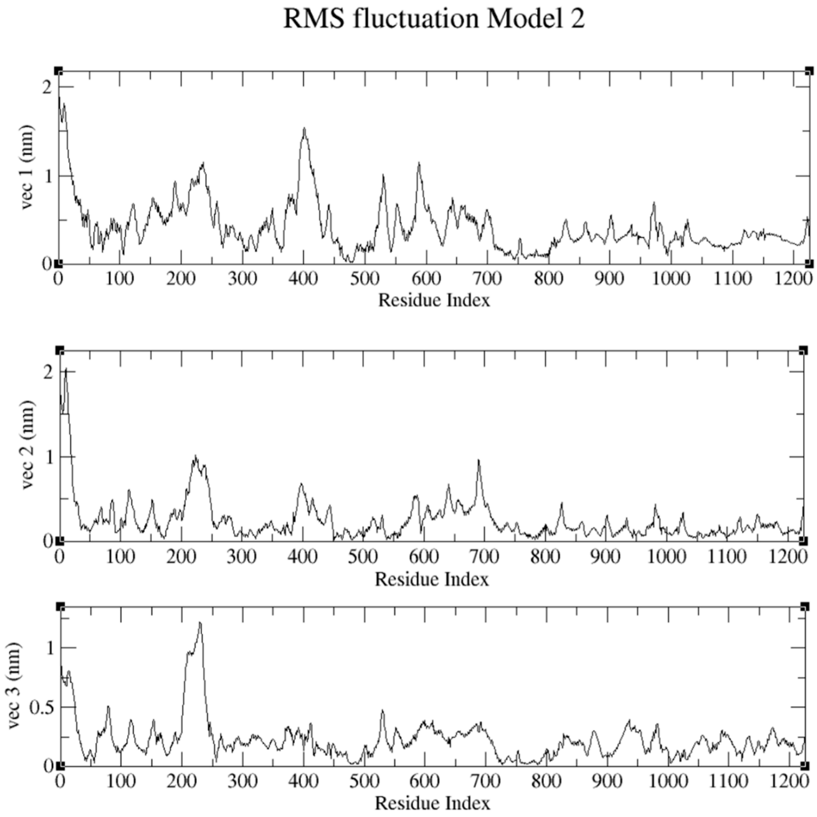

a)

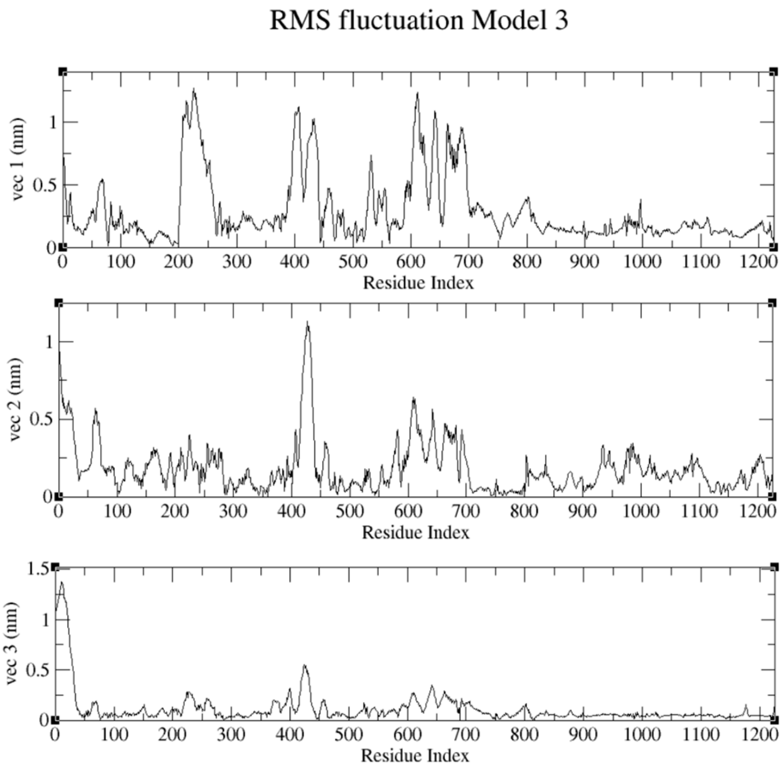
RMS fluctuation Model 3
vec 1 (nm)
vec 2 (nm)
vec 3 (nm)
Residue Index

b)

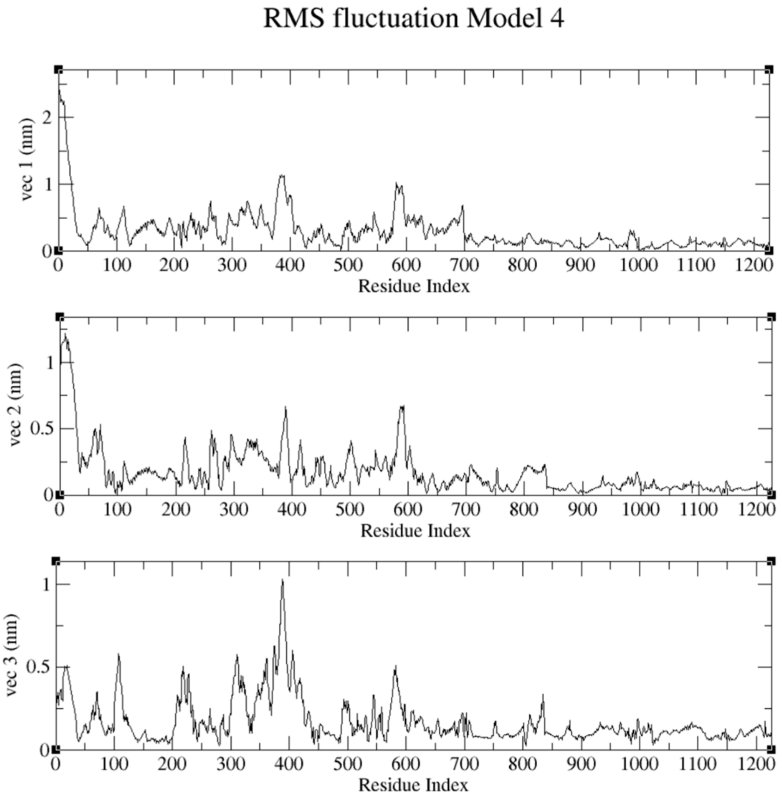
RMS fluctuation Model 4
vec 1 (nm)
vec 2 (nm)
vec 3 (nm)
Residue Index

c)

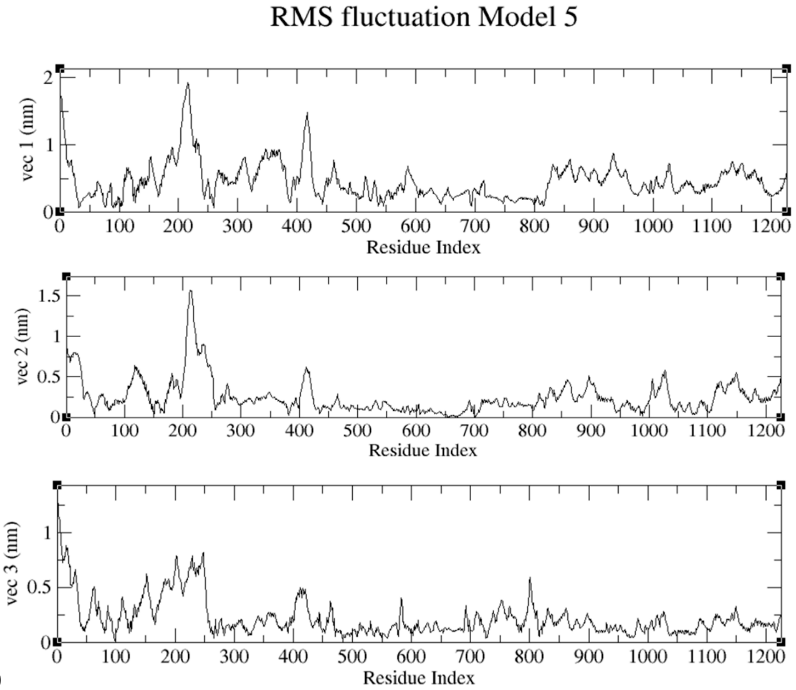

d)

Figure S12. RMSF for PCs 1, 2, and 3 for AlphaFold included models a) 2, b) 3, c) 4, and d) 5.

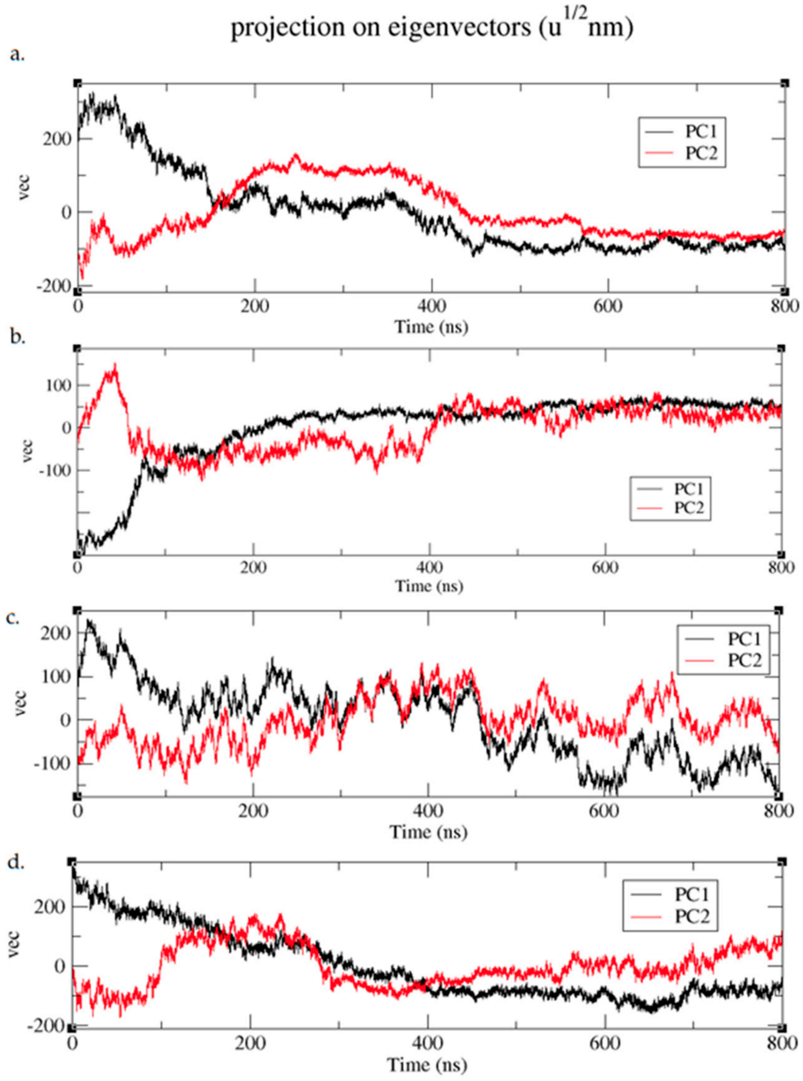

Figure S13. PC1 and PC2 projection plots for AlphaFold included models: a) Model-2, b) Model-3, c) Model-4, and d) Model-5.

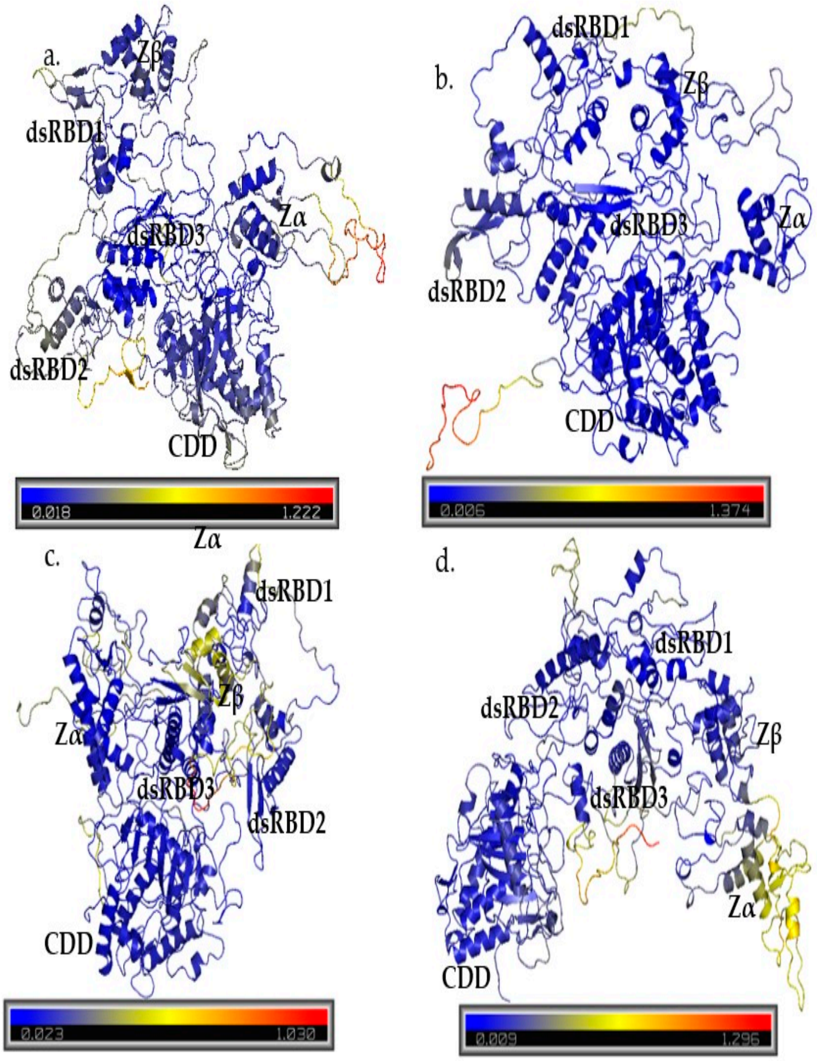

Figure S14. RMSF of PC3 for AlphaFold included models 2-5, (a-d). Color spectra spans from blue, to yellow, to red, where blue represents areas that stay conserved and red represents areas with the highest structural variation.

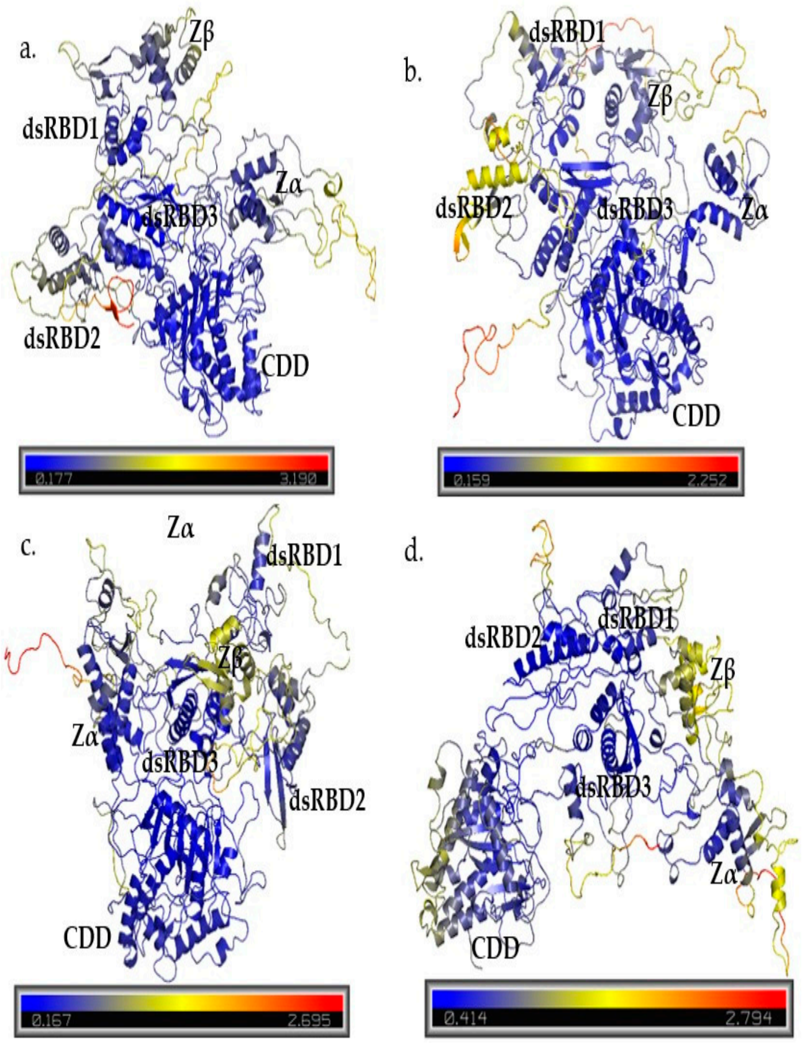

Figure S15. Overall RMSF for AlphaFold-included models 2-5, a-d. Color spectra spans from blue, to yellow, to red, where blue represents areas that stay rigid and red represents areas with the highest flexibility.

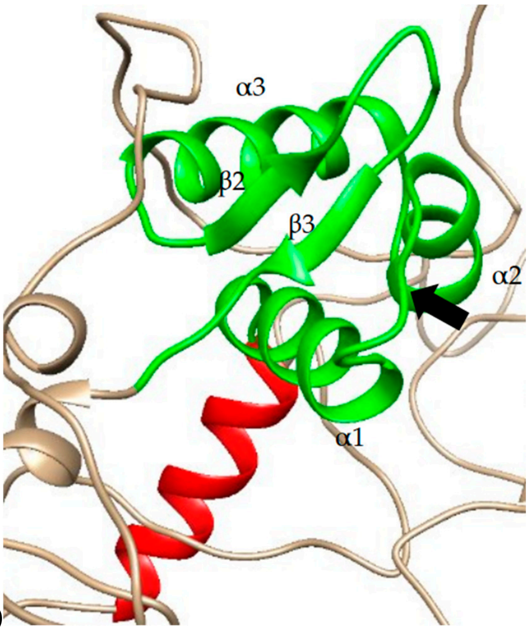

a)

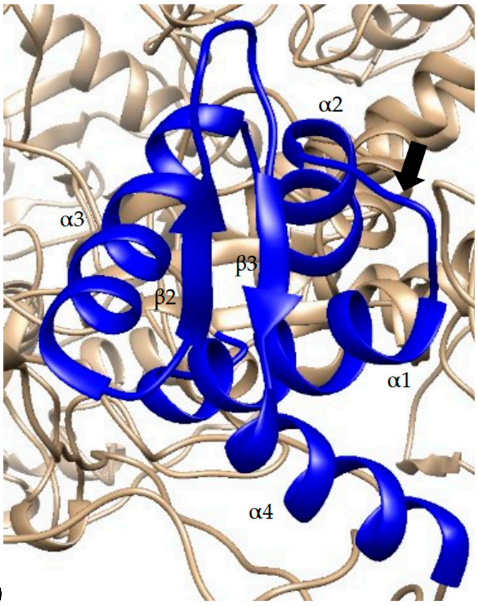

b)

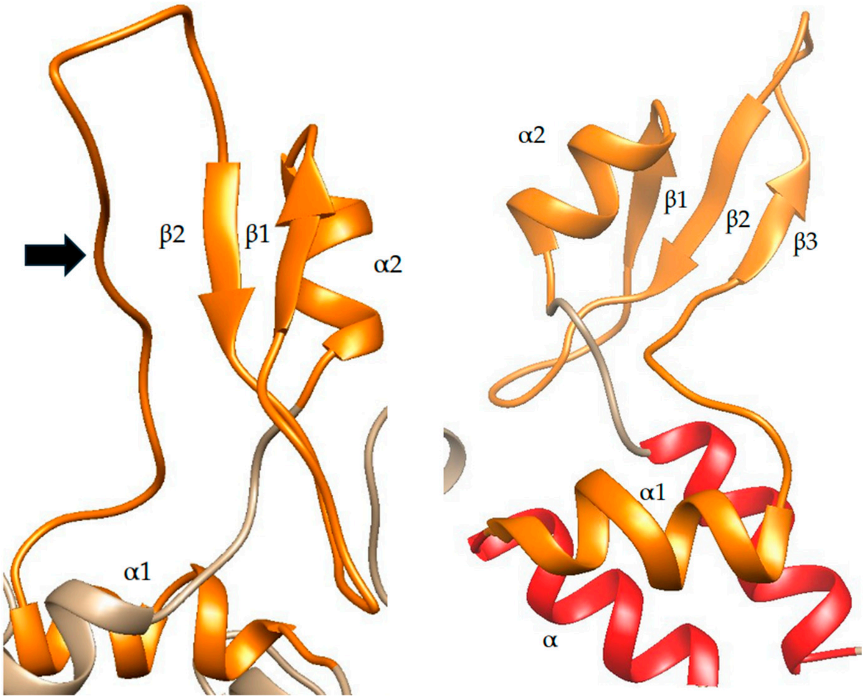

c)

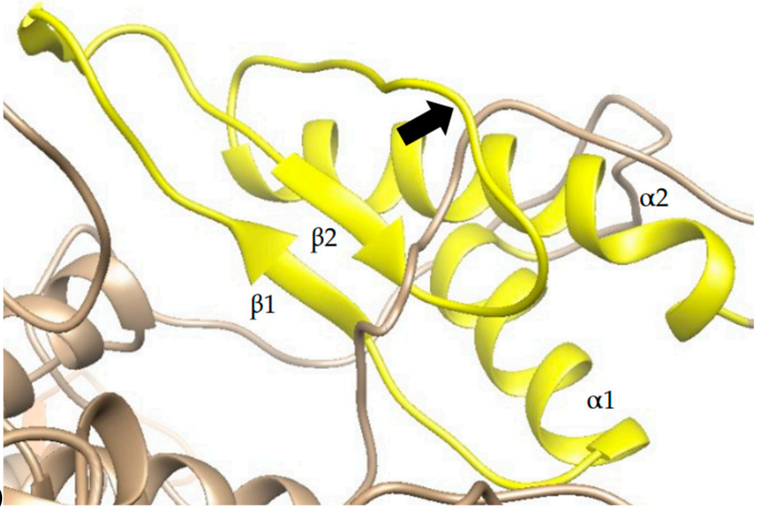

d)

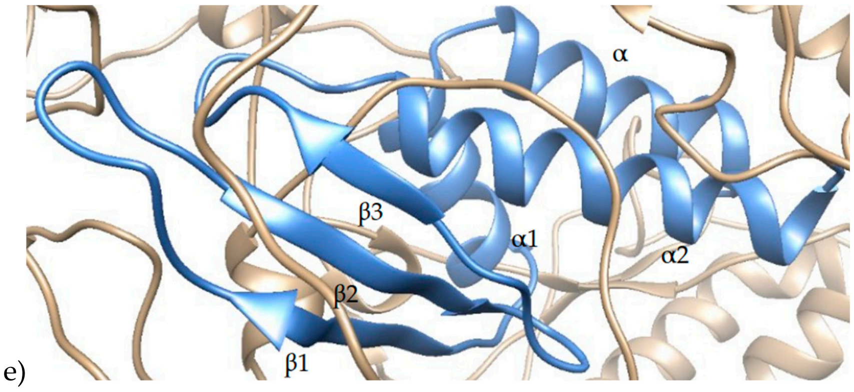

e)

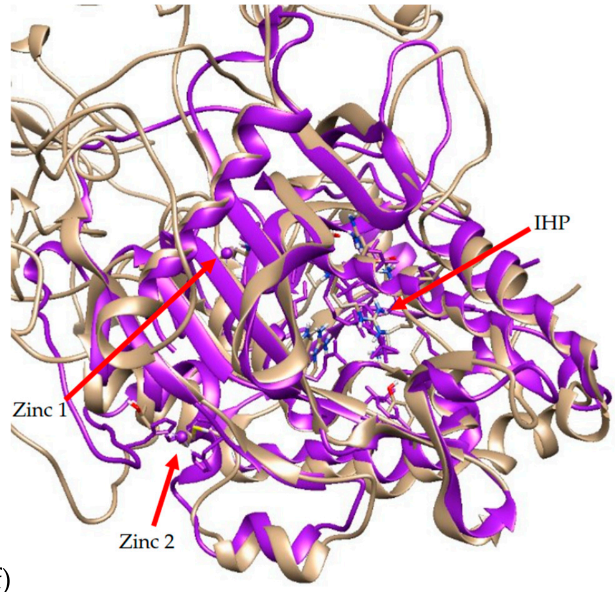

f)

Figure S16. Visualization of ADAR1 ordered domains for AlphaFold included model 2 representative structure from 694,720 ps. Black arrows point to areas where a β sheet should appear. a) Zα DBD in green, helix not identified in PDB colored red, b) Zβ domain in blue, c) two structures of dsRBD1 from two basins of ADAR1 mod 2, left 694,720 ps and right 493,890 ps where the dsRBD1 is orange and helices not identified in PDB are shown in red, d) dsRBD2 in yellow, e) dsRBD3 in cornflower blue, f) comparison of CDD to PDB: 9B83 in purple.

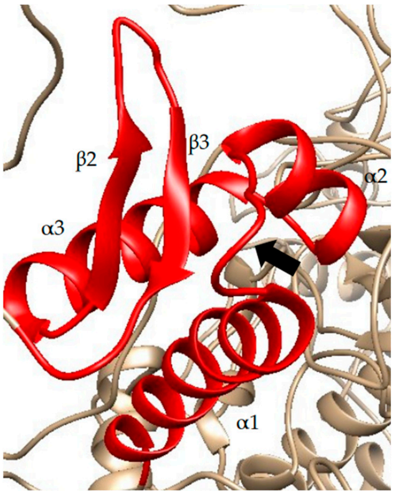

a)

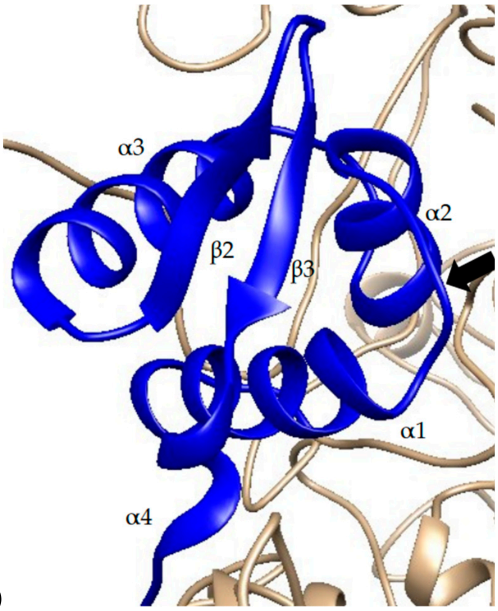

b)

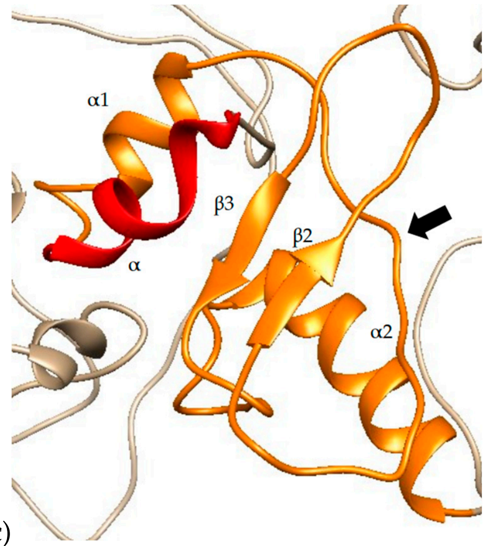

c)

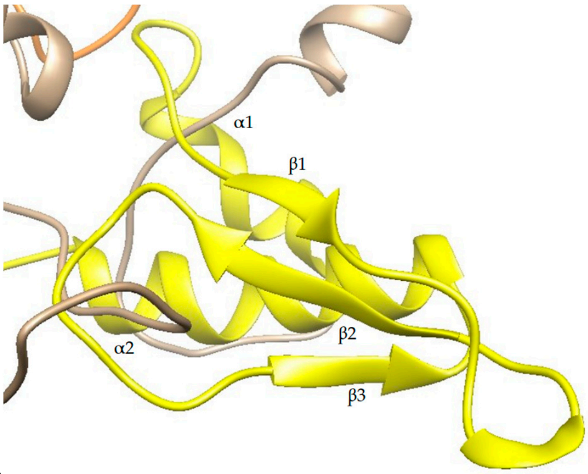

d)

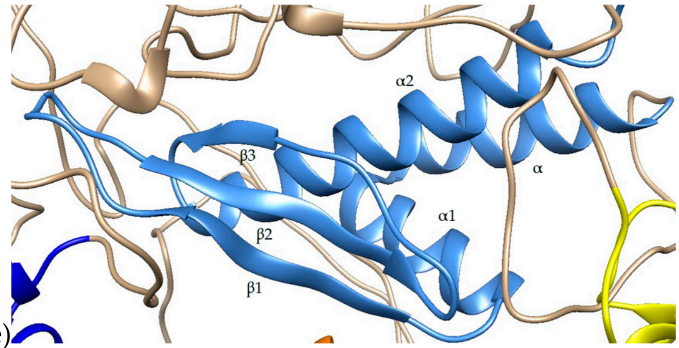

e)

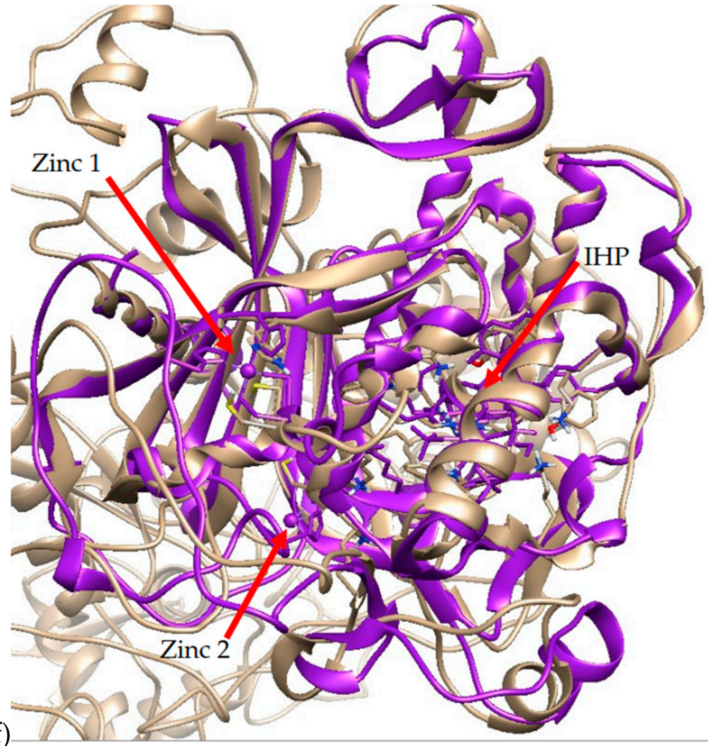

f)

Figure S17. Visualization of ADAR1 ordered domains for AlphaFold included model 3 representative structure from 726,960 ps. Black arrows point to areas where a β sheet should appear. a) Zα DBD in red, b) Zβ domain in blue, c) dsRBD1 in orange and helix not identified in PDB is shown in red, d) dsRBD2 in yellow. e) dsRBD3 in cornflower blue, f) comparison of CDD to PDB: 9B83 in purple.

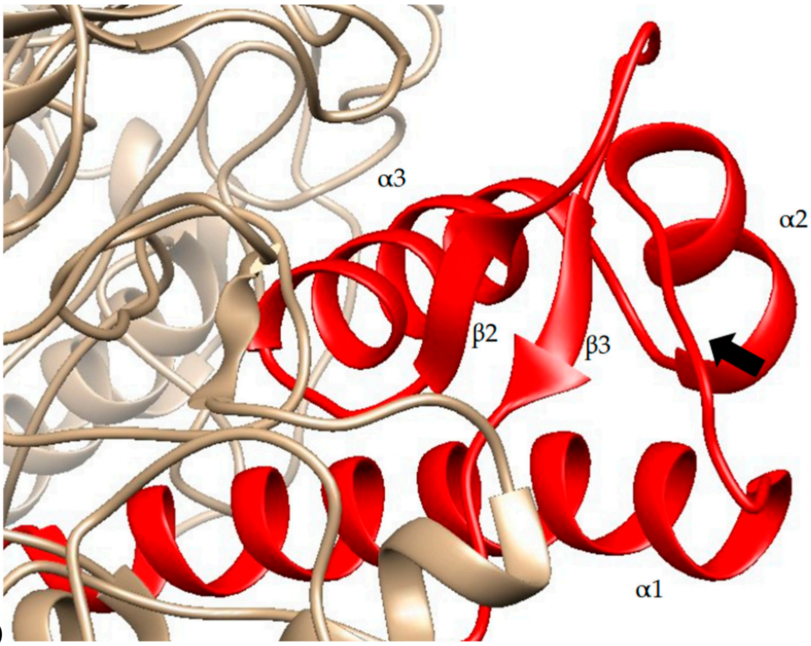

a)

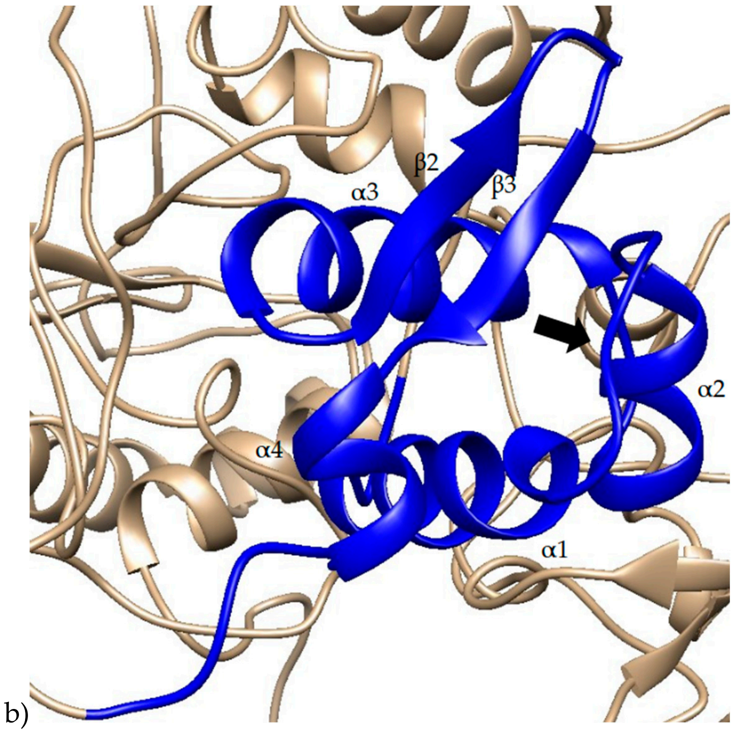

b)

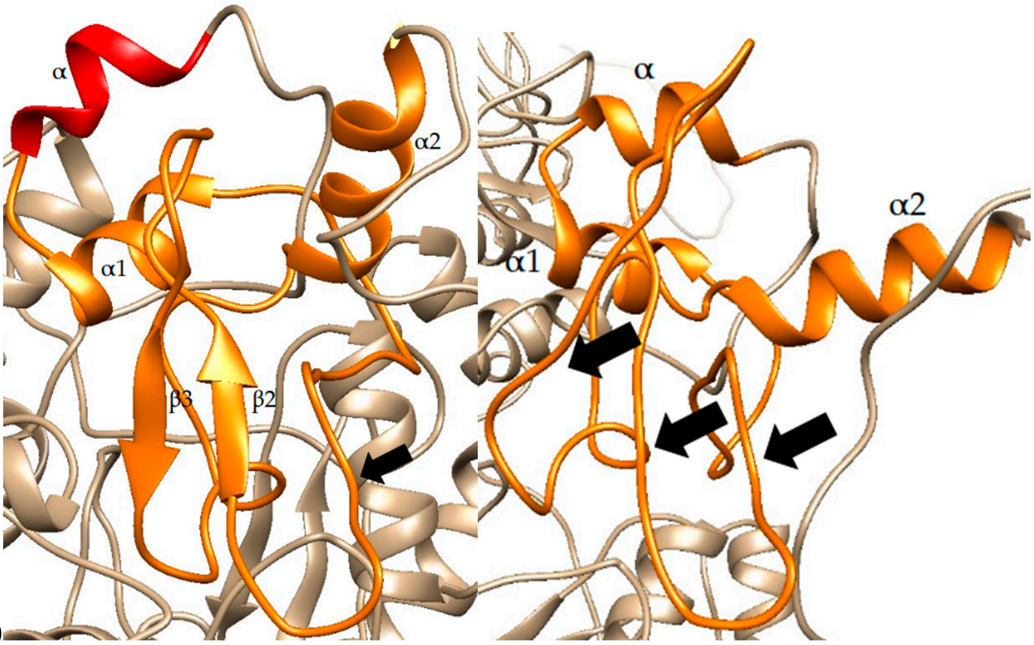

c)

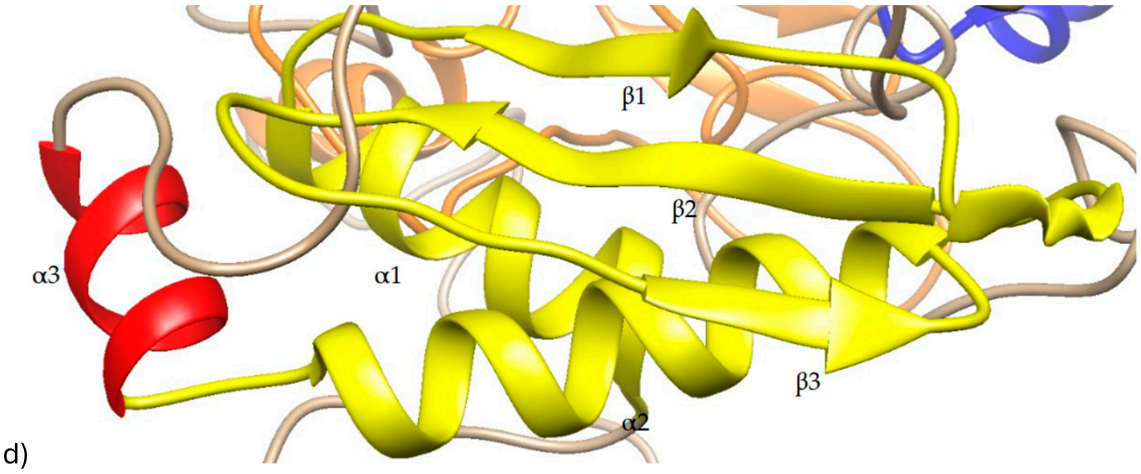

d)

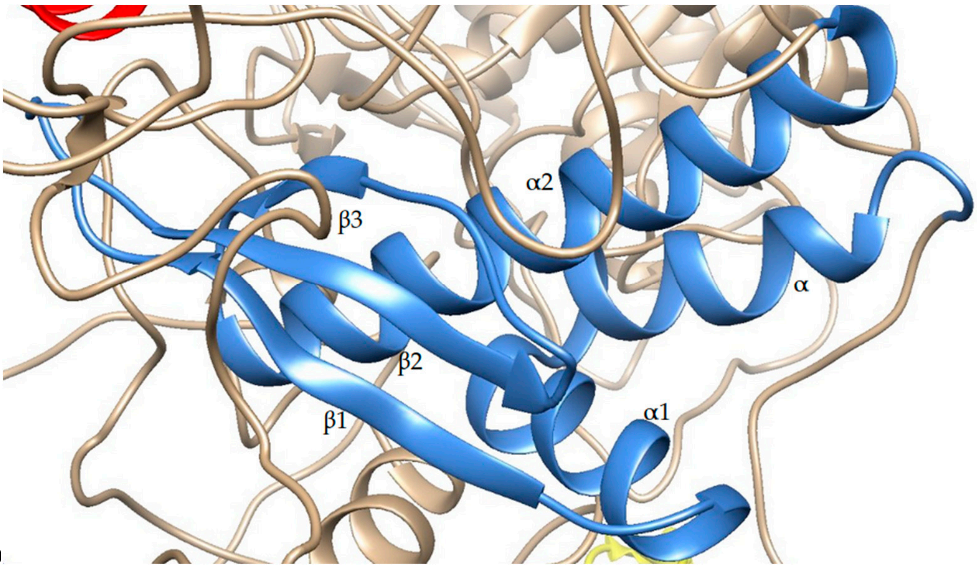

e)

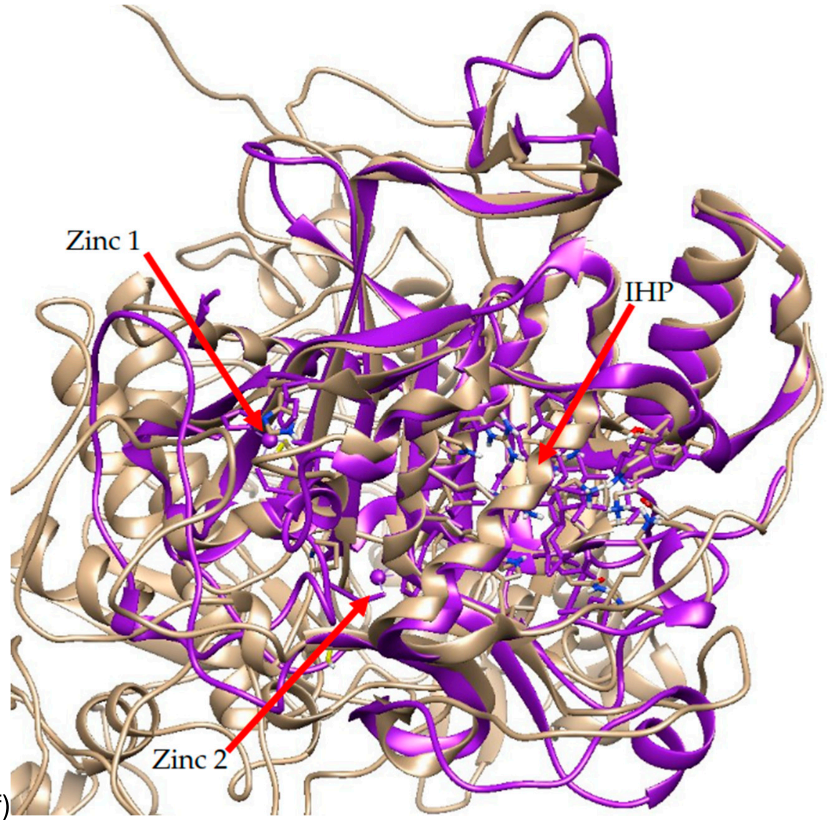

f)

Figure S18. Visualization of ADAR1 ordered domains for AlphaFold included model 4 representative structure from 768,750 ps. Black arrows point to areas where a β sheet should appear. a) Zα DBD in red, b) Zβ domain in blue, c) on left dsRBD1 from 768,750 ps and on right dsRBD1 from 656,580 ps, dsRBD1 in orange and helix not identified in PDB is shown in red, d)

dsRBD2 in yellow, potential new helix in red. e) dsRBD3 in cornflower blue, f) comparison of CDD to PDB: 9B83 in purple.

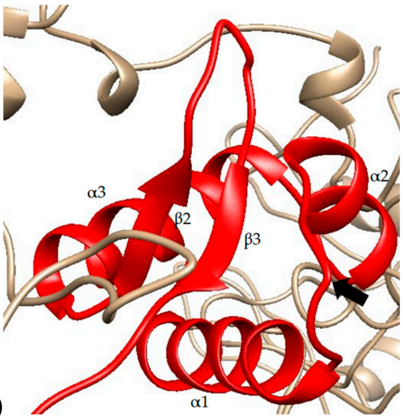

a)

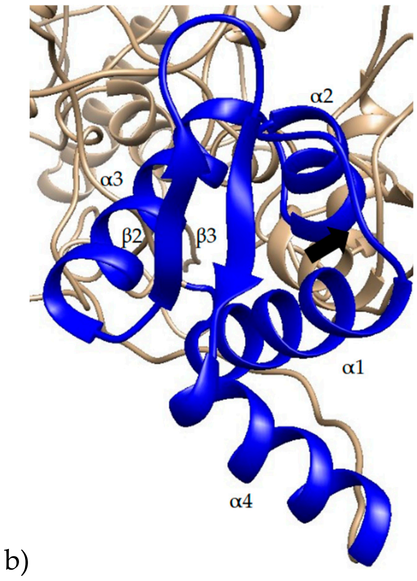

b)

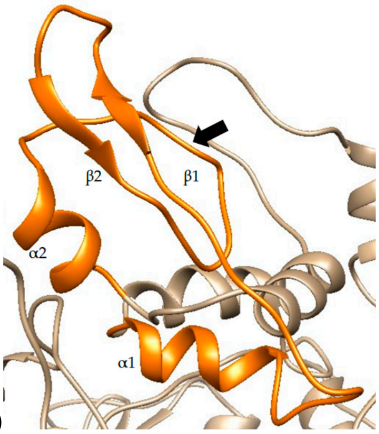

c)

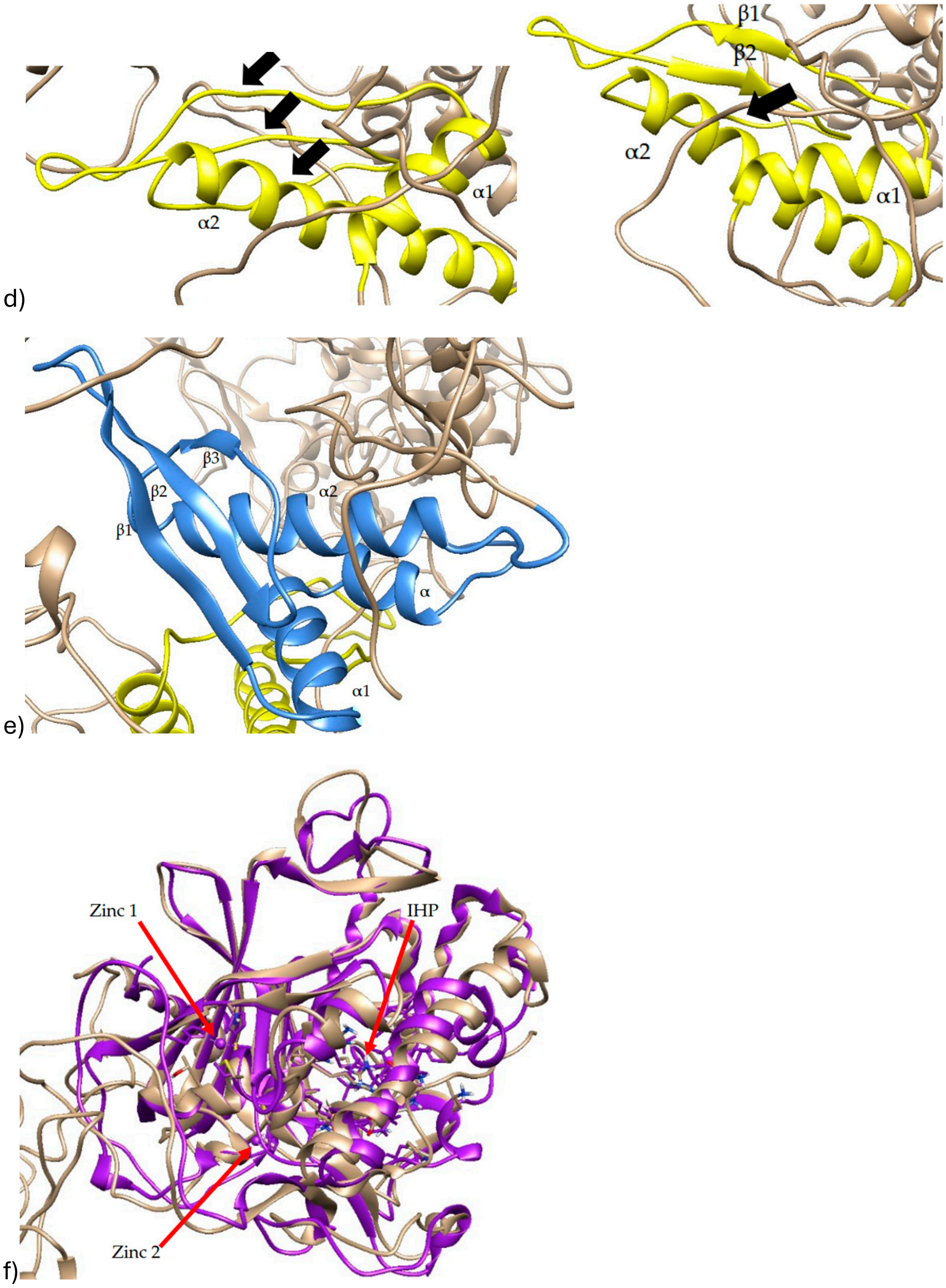

Figure S19. Visualization of ADAR1 ordered domains for AlphaFold included model 5 representative structure from 491,870 ps. Black arrows point to areas where a β sheet should appear. a) Z*α* DBD in red, b) Zβ domain in blue, c) dsRBD1 in orange, d) dsRBD2 in yellow, left

froom 491,870 ps, and right from 549,210 ps, e) dsRBD3 in cornflower blue, f) comparison of CDD to PDB: 9B83 in purple.

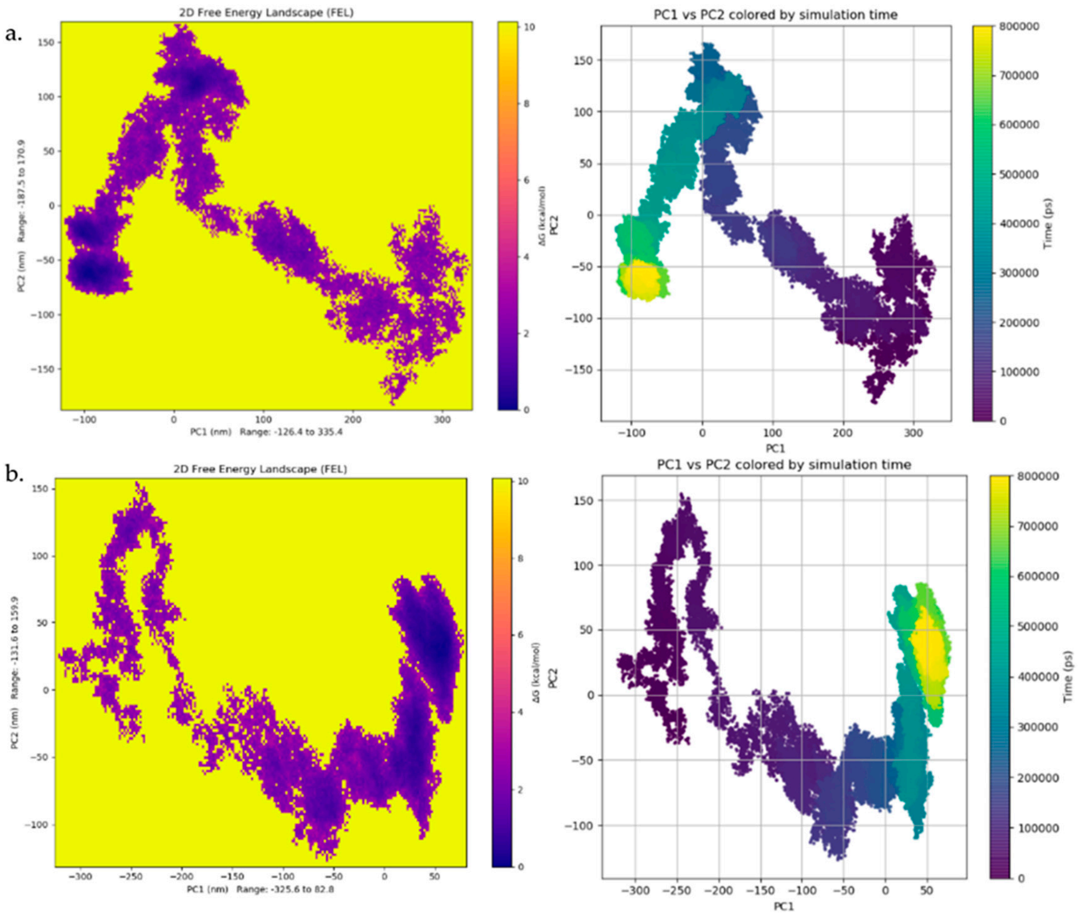

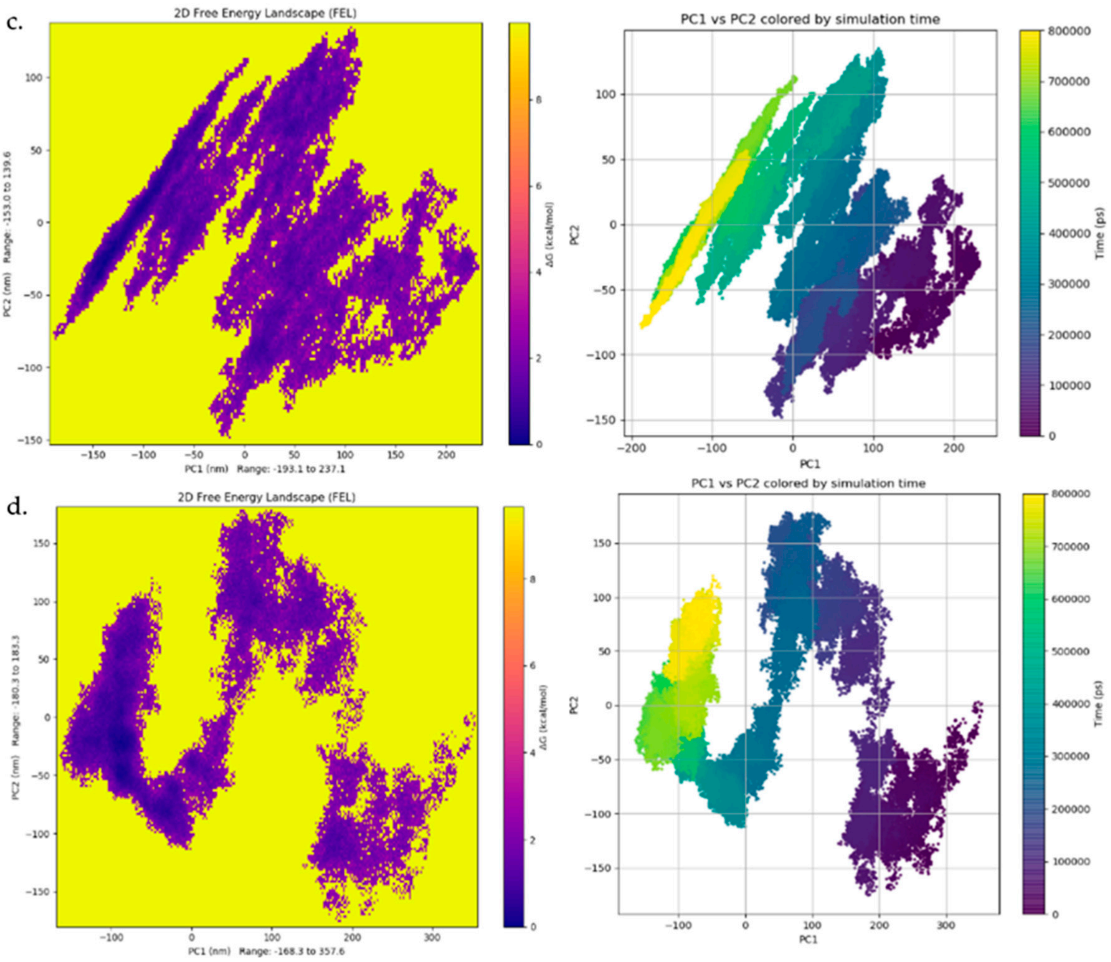

Figure S20. For each of the AlphaFold included models: a) Model-2, b) Model-3, c) Model-4, and d) Model-5, on the left is the 2D free energy landscapes colored by ΔG energy and on the right is the 2D free energy landscapes colored by simulation time.

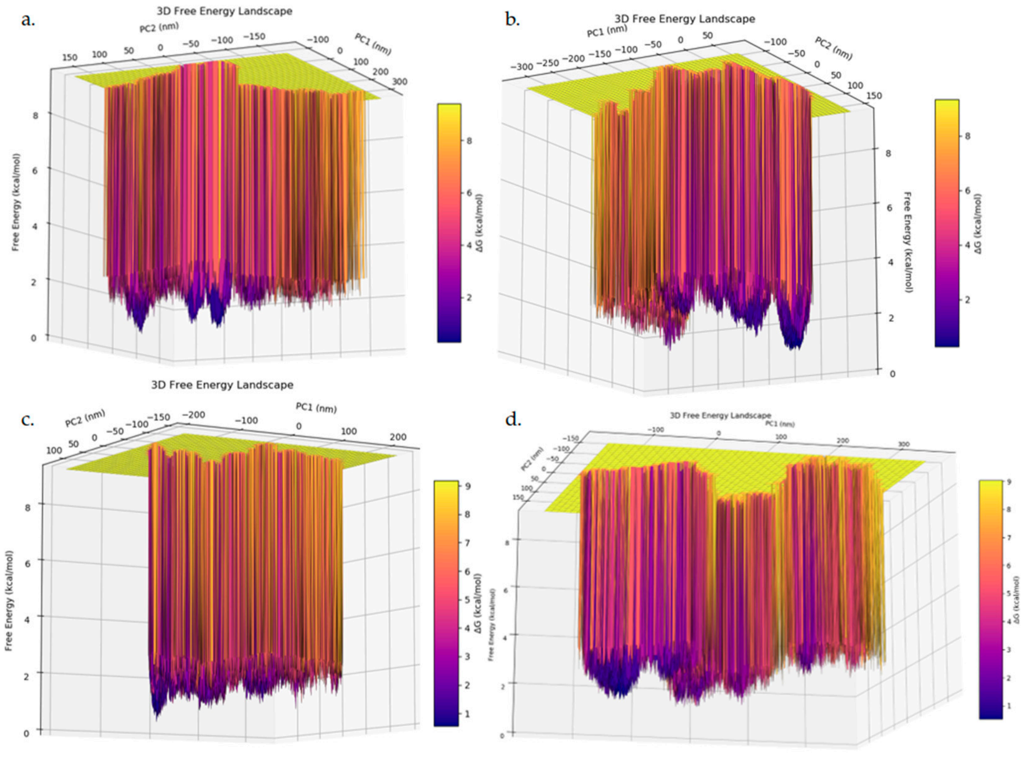

Figure S21. 3D free energy landscapes for AlphaFold included models: a) Model-2, b) Model-3, c) Model-4, and d) Model-5.

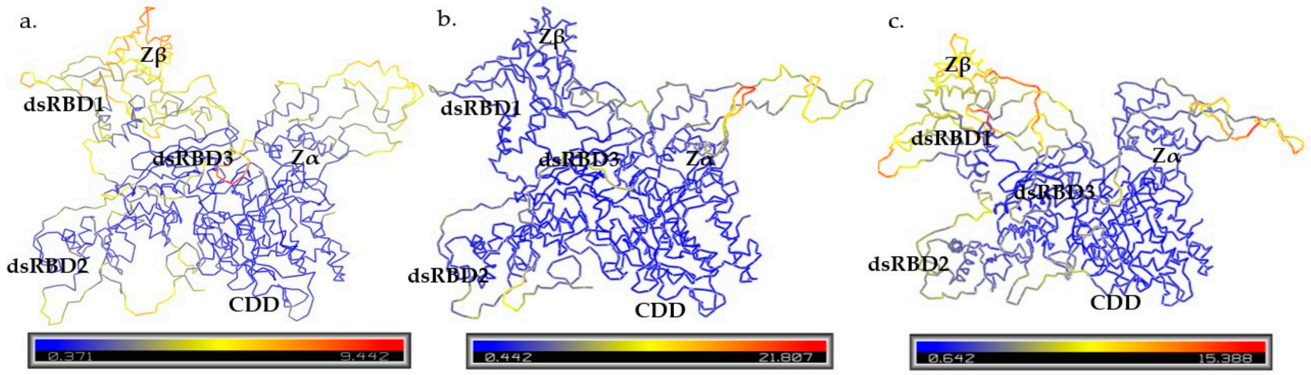

Figure S22. RMSD between 10 structures for basins of AlphaFold-included model 2: a. 500-600 ns, b.

600-700 ns, and c. 700-800ns. Color spectra spans from blue, to yellow, to red, where blue represents areas that are rigid and red represents areas with large variation.

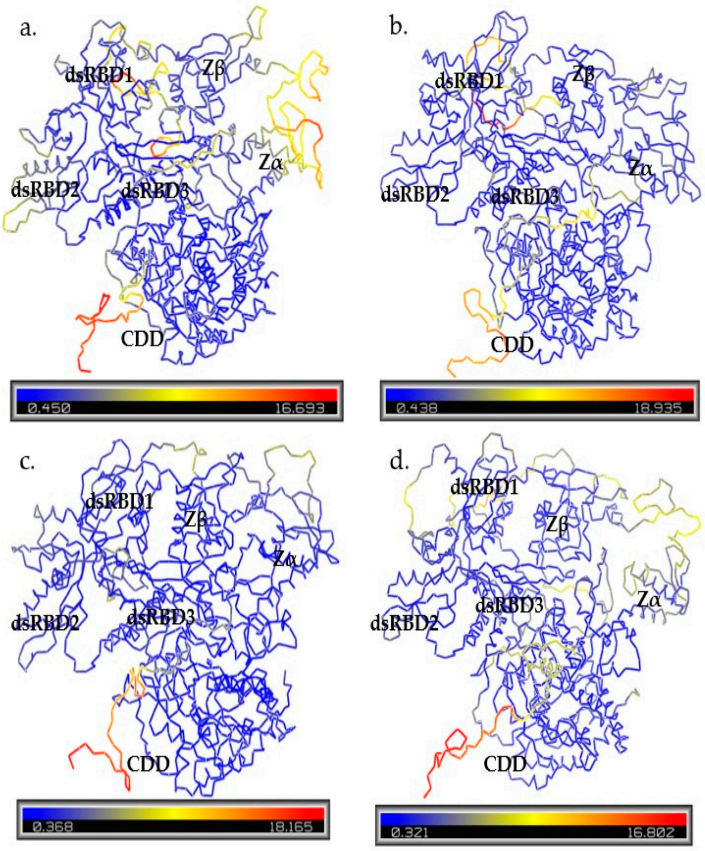

Figure S23. RMSD between 10 structures for basins of AlphaFold-included model 3: a. 100-300 ns, b. 300-400 ns, c. 400-600ns, and d. 600-800ns. Color spectra spans from blue, to yellow, to red, where blue represents areas that are rigid and red represents areas with large variation.

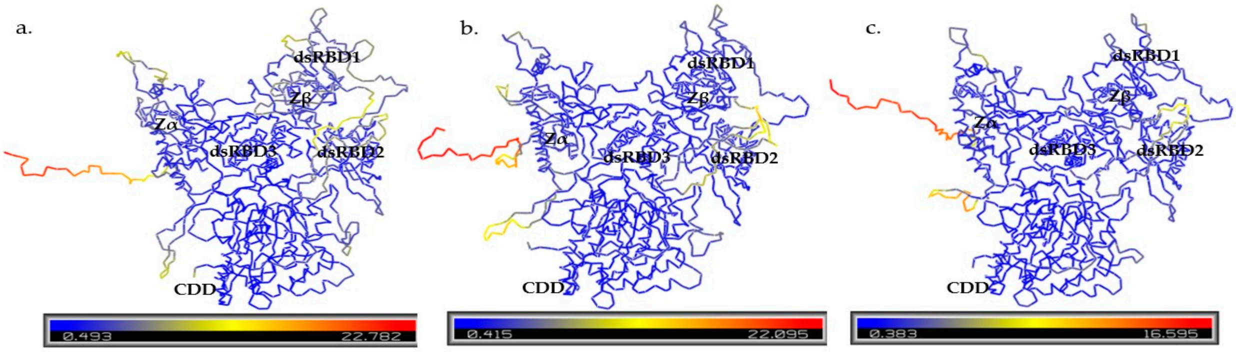

Figure S24. RMSD between 10 structures for basins of AlphaFold-included model 4: a. 500-600 ns, b. 600-700 ns, and c. 700-800ns. Color spectra spans from blue, to yellow, to red, where blue represents areas that are rigid and red represents areas with large variation.

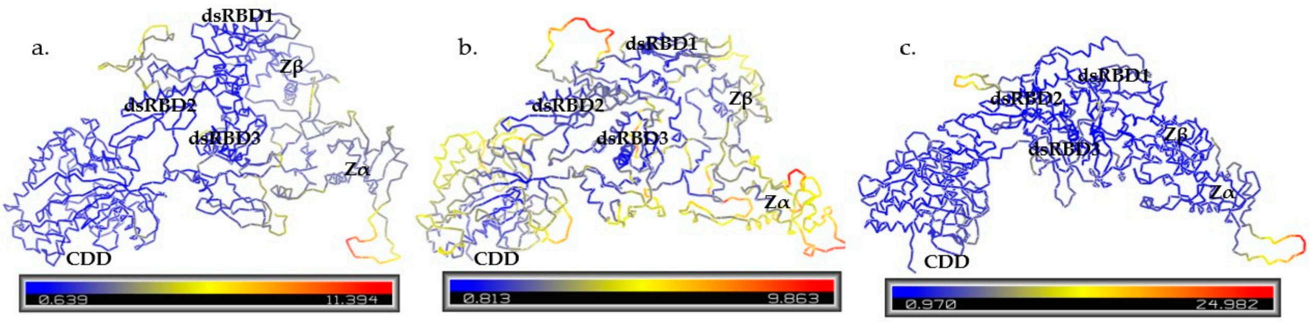

Figure S25. RMSD between 10 structures for basins of AlphaFold-included model 5: a. 300-400 ns, b. 500-600 ns, and c. 600-800ns. Color spectra spans from blue, to yellow, to red, where blue represents areas that are rigid and red represents areas with large variation.

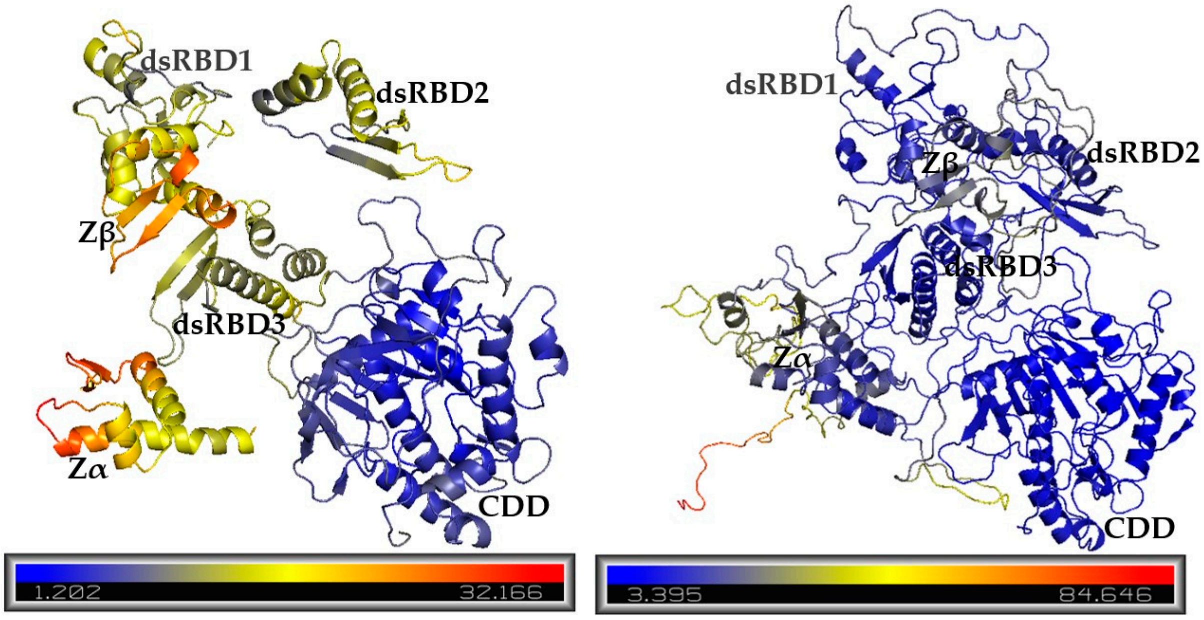

Figure S26. RMSD between lowest energy conformers for AlphaFold-included models, (left) is ordered domains only, and (right) entire structures. Color spectra spans from blue, to yellow, to red, where blue represents areas that are rigid and red represents areas with large variation.
