# Supplementary material for: In Silico Characterization of ADAR1: Structure, Dynamics, and Functional Implications
Source: Curr Issues Mol Biol. 2025 Nov 18;47(11):958. doi: 10.3390/cimb47110958 (PMC12651035; doi:10.3390/cimb47110958)
Supplement: Supplementary file 1 [file cimb-47-00958-s001.zip › Supporting_Information_Tables_ADAR1_S_11-17-25.pdf]

Table S1. Summary of published ADAR1 structures.

PDB	Domain	Coverage	Capture Method, Resolution	Reference
1QBJ	Zα	134–199	X-ray diffraction, 2.10 Å	[1]
1QGP	Zα	126–201	Solution NMR, 200 conformers, 15 submitted	[2]
2GXB	Zα	140–201	X-ray diffraction, 2.25 Å	[3]
2ACJ	Zα	140–196	X-ray diffraction, 2.60 Å	[4]
3F21, 3F22, 3F23	Zα	133–199	X-ray diffraction, 2.20 Å, 2.5 Å, 2.7 Å	[5]
1XMK	Zβ	294–366	X-ray diffraction, 0.97 Å	[6]
7ZJ1, 7ZLQ	dsRBD3	716–797	X-ray diffraction, 1.65 Å, 2.80 Å	[7]
2MDR	dsRBD3	708–801	Solution NMR, 100 conformers, 20 submitted	[8]
9B89	dsRBD3-CDD	742–823, 839–1,223	Electron microscopy, 3.87 Å	[9]
Homology Model S_0001_1432	CDD	833–1,226	RosettaCM + Experimental Constraints	[10]
9B83, 9B84	CDD	839–1,224	Electron microscopy, 3.01 Å, 3.2 Å	[9]

Table S2. Results from RosettaCM for each residue range.

Residues	Length	C-Score
1–250	250 aa	0.60
150–350	201 aa	0.68
240–410	171 aa	0.46
360–550	191 aa	0.57
500–750	251 aa	0.55
700–900	201 aa	0.44

Table S3. Structure comparison of ADAR1 initial models to experimental structures of ADAR1 domains: Zα (PDB: 1QBJ), Zβ (PDB: 1XMK), dsRBD3 (PDB: 2MDR), CDD (PDB: 9B83), and dsRBD3-CDD (PDB:9B89).

		PDB				
		1QBJ	1XMK	2MDR	9B83	9B89
Model 1	TM-score	0.84323	0.82491	0.76774	0.49807	0.47877
	RMSD Value (Å)	1.38	1.68	2.20	6.32	6.87
	Aligned Length	63	71	88	297	348
Model 2	TM-score	0.84854	0.82065	0.75320	0.51985	0.48965
	RMSD Value (Å)	1.35	1.65	2.27	6.12	6.79
	Aligned Length	63	71	88	303	352
Model 3	TM-score	0.83878	0.81722	0.78815	0.53975	0.50590
	RMSD Value (Å)	1.42	1.80	1.98	5.83	6.41
	Aligned Length	63	72	87	301	348
Model 4	TM-score	0.83530	0.81076	0.78455	0.56099	0.49707
	RMSD Value (Å)	1.41	1.71	2.21	5.54	6.15
	Aligned Length	63	71	89	303	330
Model 5	TM-score	0.83033	0.78325	0.76925	0.52054	0.48462

	RMSD Value (Å)	1.48	1.99	2.04	6.26	6.67
	Aligned Length	63	71	86	305	344

Table S4. SAVEs evaluation initial 5 models from homology modeling.

Model	ERRAT	Verify3D	Procheck			
			Favored	Allowed	Generously Allowed	Disallowed
Model 1	28.8668	53.08%	74.5%	15.9%	6.3%	3.2%
Model 2	29.0429	46.92%	73.6%	15.8%	5.9%	4.7%
Model 3	26.7327	52.97%	74.4%	16.5%	5.9%	3.2%
Model 4	27.8053	52.28%	73.0%	15.7%	6.2%	5.1%
Model 5	24.9793	50.34%	72.3%	17.0%	6.9%	3.9%

Table S5. SAVEs evaluation of AlphaFold-included models with domains only.

Model	ERRAT	VERIFY3D	PROCHECK			
			Favored	Allowed	Generously Allowed	Disallowed
Model 1 Prior	68.4005	80.47%	92.7%	6.4%	0.9%	0.0%
Model 2 Prior	64.1404	81.00%	91.7%	7.6%	0.3%	0.4%
Model 2 Post	90.708	70.73%	90.8%	7.3%	1.3%	0.6%
Model 3 Prior	74.8698	80.62%	93.0%	6.7%	0.1%	0.1%

Model 3 Post	90.1429	74.07%	90.8%	7.1%	1.8%	0.3%
Model 4 Prior	75.9791	83.06%	92.40%	6.8%	0.1%	0.6%
Model 4 Post	94.1964	63.80%	89.80%	8.6%	1.5%	0.1%
Model 5 Prior	71.4286	80.87%	93.0%	6.4%	0.6%	0.0%
Model 5 Post	87.7013	58.66%	88.9%	9.3%	1.2%	0.6%

Table S6. SAVEs evaluation of five models from initial homology modeling after MD simulation.

**Model**	**ERRAT**	**VERIFY3D**	**PROCHECK**			
			**Favored**	**Allowed**	**Generously Allowed**	**Disallowed**
Model 1	80.8429	56.16%	84.9%	11.4%	2.6%	1.0%
Model 2	80.4752	50.46%	83.7%	12.3%	2.7%	1.3%
Model 3	83.2512	47.95%	84.8%	11.5%	2.6%	1.0%
Model 4	86.608	55.25%	85.8%	11.1%	1.9%	1.2%
Model 5	82.6979	47.72%	83.9%	12.3%	2.8%	1.0%

Table S7. Structure comparison of ADAR1 initial models to experimental structures of ADAR1 domains: Zα (PDB: 1QBJ), Zβ (PDB: 1XMK), dsRBD3 (PDB: 2MDR), CDD (PDB: 9B83), and dsRBD3-CDD (PDB:9B89) after MD simulation.

	**PDB**

		1QBJ	1XMK	2MDR	9B83	9B89
Model 1	TM-score	0.60908	0.62561	0.71101	0.36592	0.32282
	RMSD Value (Å)	2.99	2.98	2.25	6.96	7.24
	Aligned Length	65	73	80	240	250
Model 2	TM-score	0.80855	0.79986	0.70569	0.37737	0.36669
	RMSD Value (Å)	1.54	1.81	2.82	7.04	7.39
	Aligned Length	63	73	88	248	288
Model 3	TM-score	0.65005	0.79262	0.73518	0.30704	0.26990
	RMSD Value (Å)	2.70	2.04	2.54	6.91	7.11
	Aligned Length	65	72	84	196	203
Model 4	TM-score	0.76600	0.78903	0.71853	0.43379	0.43025
	RMSD Value (Å)	2.03	2.04	3.10	6.57	7.15
	Aligned Length	63	73	90	273	331
Model 5	TM-score	0.57635	0.61930	0.70772	0.34448	0.30199
	RMSD Value (Å)	3.01	3.11	2.52	7.15	7.38
	Aligned Length	62	68	83	224	232

Table S8. Reported RNA/DNA interaction residues from published ADAR1 structures.

PDB	Domain	RNA/DNA	Interaction Residues	Reference
1QBJ,1QGP	Zα	6-bp DNA duplex d(CGCGCG)$_2$	Helix α3: Lys169, Lys170, Asn173, Arg174, Tyr177<br>β-hairpin: Thr191, Pro192, Pro193, Trp195	Schwartz and Schade 1999
2GXB	Zα	dUr(CG)$_{3\ \text{duplex RNA}}$	Helix α3: Lys169, Lys170, Asn173, Tyr177<br>β-hairpin: Lys187, Thr191, Trp195	Placido 2007
3F21	Zα	d(CACGTG)$_2$	Helix α3: *Lys169*, *Lys170*, Asn173, *Arg174*, Tyr177	Ha 2008

3F22	Zα	d(CGTACG)$_2$	β-hairpin: *Thr191*, Pro192, Pro193, Trp195	
3F23	Zα	d(CGGCCG)$_2$		
7ZLQ	dsRBD3	5′-CGAAGCCUUCGCG-3'	Helix aN: R721, N726<br>Helix a1: E733, R736<br>B1-B2 loop: H754, P753<br>KKxxK motif: K777, K778,	Mboukou 2024
9B89	dsRBD3-CDD	71-MER (5-HT$_{2C}$R)	K777, K778, K781	Deng 2025
9B83	CDD	31-MER (GLI1)	E912, N891, T1121, R892, K1115, N1106, N994, K996, K999, R1001, R1030, E1008, K1120	Deng 2025
9B84	CDD	66-MER (5-HT$_{2C}$R)	E912, N891, R892, K895, K1115, K999, R1001, R1030, E1008, N1006, K1120	Deng 2025

Table S9. Lowest 10 energy structures for each AlphaFold-included model.

	Time (ps)	Energy (kJ/mol)
Model 2	529880	-14013.732422
	555380	-13678.484375
	554950	-13578.454102
	555320	-13459.929688
	535710	-13437.886719
	529170	-13380.388672
	500430	-13353.529297
	529900	-13317.344727
	554920	-13271.275391
	555020	-13208.341797
Model 3	183150	-13675.204102
	506360	-13165.665039
	183060	-13120.180664
	506330	-12950.899414
	784050	-12834.384766
	183160	-12693.061523
	183020	-12616.165039
	504340	-12562.269531
	464840	-12548.101562
	506400	-12537.928711
Model 4	730760	-14960.472656
	730740	-14097.222656
	730770	-14063.739258
	730950	-13986.317383
	730800	-13895.233398
	730790	-13839.129883
	749710	-13700.018555

	730730	-13690.258789
	730920	-13675.143555
	730660	-13597.994141
Model 5	679160	-13329.839844
	640860	-13109.09375
	623080	-12870.779297
	501600	-12808.587891
	622630	-12808.12793
	683590	-12804.833008
	622940	-12785.386719
	679470	-12765.411133
	623180	-12749.594727
	622950	-12745.542969

References:

1. Schwartz, T.; Rould, M.A.; Lowenhaupt, K.; Herbert, A.; Rich, A. Crystal Structure of the Zα Domain of the Human Editing Enzyme ADAR1 Bound to Left-Handed Z-DNA. *Science* **1999**, *284*, 1841–1845. https://doi.org/10.1126/SCIENCE.284.5421.1841.
2. Schade, M.; Turner, C.J.; Kühne, R.; Schmieder, P.; Lowenhaupt, K.; Herbert, A.; Rich, A.; Oschkinat, H. The Solution Structure of the Za Domain of the Human RNA Editing Enzyme ADAR1 Reveals a Prepositioned Binding Surface for Z-DNA. *Proc. Natl. Acad. Sci. USA* **1999**, *96*, 12465–12470. https://doi.org/10.1073/PNAS.96.22.12465/.
3. Placido, D.; Brown, B.A.; Lowenhaupt, K.; Rich, A.; Athanasiadis, A. A Left-Handed RNA Double Helix Bound by the Zα Domain of the RNA-Editing Enzyme ADAR1. *Structure* **2007**, *15*, 395–404. https://doi.org/10.1016/j.str.2007.03.001.
4. Sung, C.H.; Lowenhaupt, K.; Rich, A.; Kim, Y.G.; Kyeong, K.K. Crystal Structure of a Junction Between B-DNA and Z-DNA Reveals Two Extruded Bases. *Nature* **2005**, *437*, 1183–1186. https://doi.org/10.1038/nature04088.
5. Ha, S.C.; Choi, J.; Hwang, H.Y.; Rich, A.; Kim, Y.G.; Kim, K.K. The Structures of Non-CG-Repeat Z-DNAs Co-Crystallized with the Z-DNA-Binding Domain, HZαADAR1. *Nucleic Acids Res.* **2009**, *37*, 637. https://doi.org/10.1093/NAR/GKN976.
6. Athanasiadis, A.; Placido, D.; Maas, S.; Brown, B.A.; Lowenhaupt, K.; Rich, A. The Crystal Structure of the Zβ Domain of the RNA-Editing Enzyme ADAR1 Reveals Distinct Conserved Surfaces Among Z-Domains. *J. Mol. Biol.* **2005**, *351*, 496–507. https://doi.org/10.1016/J.JMB.2005.06.028.
7. Mboukou, A.; Rajendra, V.; Messmer, S.; Catala, M.; Tisné, C.; Jantsch, M.F.; Barraud, P. Dimerization of ADAR1 Modulates Site-Specificity of RNA Editing. *bioRxiv* **2023**, *15*, 570066. https://doi.org/10.1101/2023.12.05.570066.
8. Barraud, P.; Banerjee, S.; Mohamed, W.I.; Jantsch, M.F.; Allain, F.H.T. A Bimodular Nuclear Localization Signal Assembled via an Extended Double-Stranded RNA-Binding Domain Acts as an RNA-Sensing Signal for Transportin 1. *Proc. Natl. Acad. Sci. USA* **2014**, *111*, E1852–E1861. https://doi.org/10.1073/PNAS.1323698111/.
9. Deng, X.; Sun, L.; Zhang, M.; Basavaraj, R.; Wang, J.; Weng, Y.-L.; Gao, Y. Biochemical Profiling and Structural Basis of ADAR1-Mediated RNA Editing. *Mol. Cell* **2025**, *85*, 1381–1394.e6. https://doi.org/10.1016/j.molcel.2025.02.017.
10. Park, S.H.; Doherty, E.E.; Xie, Y.; Padyana, A.K.; Fang, F.; Zhang, Y.; Karki, A.; Lebrilla, C.B.; Siegel, J.B.; Beal, P.A. High-Throughput Mutagenesis Reveals Unique Structural Features of Human ADAR1. *Nat. Commun.* **2020**, *11*, 5130. https://doi.org/10.1038/s41467-020-18862-2.
